
JOURNAL INFORMATION
==============================
NLM Title Abbreviation: J Int AIDS Soc
Journal ID: J Int AIDS Soc
NLM Journal ID: 101478566
Journal ID: JIAS

Journal of the International AIDS Society

EISSN: 1758-2652
Publisher: Wiley

ARTICLE INFORMATION
==============================
PMCID: PMC3501674
DOI: 10.7448/IAS.15.5.18440
Article ID: 18440
Article version: 1
Subjects: AIDS2012 Abstract Supplement

Track C Epidemiology and Prevention Science

Electronic publication date: 2012 Oct 22
Publication date: 2012
Volume: 15
Issue: Suppl 3
Electronic Location ID: 10.7448/IAS.15.5.18440
Copyright: © 2012 Track C Epidemiology and Prevention Science
Copyright year: 2012
License: This is an open-access article distributed under the terms of the Creative Commons Attribution License, which permits unrestricted use, distribution, and reproduction in any medium, provided the original work is properly cited.
License URL: https://creativecommons.org/licenses/by/2.0/

==============================
JOURNAL INFORMATION
==============================
NLM Title Abbreviation: J Int AIDS Soc
Journal ID: J Int AIDS Soc
NLM Journal ID: 101478566
Journal ID: JIAS

Journal of the International AIDS Society

EISSN: 1758-2652
Publisher: Wiley

ARTICLE INFORMATION
==============================
Article ID: 18440-001
Subjects: C1 - Natural history, progression and survival

WEPDB0104: HIV-1 outcompetes HIV-2 in dually infected Senegalese subjects with low CD4 counts

Raugi D.N. 1 2 §
Gottlieb G.S. 2
Sow P.S. 3
Cherne S.L. 4
Toure M. 3
Sall F. 3
Traore F. 3
Sy M.-P. 3
Zheng N.N. 5
N'Doye I. 6
Kiviat N.B. 2 4
Hawes S.E. 1
University of Washington-Dakar HIV-2 Study Group
1 University of Washington School of Public Health, Department of Epidemiology, Seattle, United States
2 University of Washington School of Medicine, Department of Medicine/Allergy & Infectious Diseases, Seattle, United States
3 Clinique des Maladies Infectieuses, CHNU de Fann, Universite Cheikh Anta Diop de Dakar, Dakar, Senegal
4 University of Washington School of Medicine, Department of Pathology, Seattle, United States
5 Fred Hutchinson Cancer Research Center, Vaccine and Infectious Disease Division, Seattle, United States
6 Institut d'Hygiene Sociale, Conseil National de la Lutte contre le SIDA, Dakar, Senegal
§ Presenting author email: raugid@u.washington.edu
Electronic publication date: 2012 Oct 22
Publication date: 2012
Volume: 15
Issue: Suppl 3
Electronic Location ID: 18440-001
Copyright: © 2012 D.N. Raugi et al.
Copyright year: 2012
License: This is an open-access article distributed under the terms of the Creative Commons Attribution License, which permits unrestricted use, distribution, and reproduction in any medium, provided the original work is properly cited.
License URL: http://creativecommons.org/licenses/by/2.0/

JOURNAL INFORMATION
==============================
NLM Title Abbreviation: J Int AIDS Soc
Journal ID: J Int AIDS Soc
NLM Journal ID: 101478566
Journal ID: JIAS

Journal of the International AIDS Society

EISSN: 1758-2652
Publisher: Wiley

ARTICLE INFORMATION
==============================
Article ID: 18440-002
Subjects: C2 - Trends in morbidity and mortality

MOAC0205: Social disparities and mortality in a large metropolitan HIV cohort

Messeri P. 1 §
Chiasson M.A. 2
1 Columbia University Mailman School of Public Health, Sociomedical Sciences, New York, United States
2 Public Health Solutions, New York, United States
§ Presenting author email: pam9@columbia.edu
Electronic publication date: 2012 Oct 22
Publication date: 2012
Volume: 15
Issue: Suppl 3
Electronic Location ID: 18440-002
Copyright: © 2012 P. Messeri et al.
Copyright year: 2012
License: This is an open-access article distributed under the terms of the Creative Commons Attribution License, which permits unrestricted use, distribution, and reproduction in any medium, provided the original work is properly cited.
License URL: http://creativecommons.org/licenses/by/2.0/

JOURNAL INFORMATION
==============================
NLM Title Abbreviation: J Int AIDS Soc
Journal ID: J Int AIDS Soc
NLM Journal ID: 101478566
Journal ID: JIAS

Journal of the International AIDS Society

EISSN: 1758-2652
Publisher: Wiley

ARTICLE INFORMATION
==============================
Article ID: 18440-003
Subjects: C2 - Trends in morbidity and mortality

MOAC0301: Recent trends in and predictors of non-AIDS death in a national cohort of HIV-diagnosed adults

Kall M. 1 §
Smith R. 1
Brown A. 1
Delpech V. 1
1 Health Protection Agency, HIV/STI Department, London, United Kingdom
§ Presenting author email: meaghan.kall@hpa.org.uk
Electronic publication date: 2012 Oct 22
Publication date: 2012
Volume: 15
Issue: Suppl 3
Electronic Location ID: 18440-003
Copyright: © 2012 M. Kall et al.
Copyright year: 2012
License: This is an open-access article distributed under the terms of the Creative Commons Attribution License, which permits unrestricted use, distribution, and reproduction in any medium, provided the original work is properly cited.
License URL: http://creativecommons.org/licenses/by/2.0/

JOURNAL INFORMATION
==============================
NLM Title Abbreviation: J Int AIDS Soc
Journal ID: J Int AIDS Soc
NLM Journal ID: 101478566
Journal ID: JIAS

Journal of the International AIDS Society

EISSN: 1758-2652
Publisher: Wiley

ARTICLE INFORMATION
==============================
Article ID: 18440-004
Subjects: C3 - Modelling HIV epidemics

MOAC0403: Estimation of HIV sexual transmission potential from IDU to general population in two Russian cities

Eritsyan K. 1 2 §
Levina O. 1
Smolskaia T. 3
White E. 4
Heimer R. 4
1 NGO of Social Projects in the Sphere of Population's Well-Being “Stellit”, Saint-Petersburg, Russian Federation
2 Saint-Petersburg State University, Department of Psychology, Saint-Petersburg, Russian Federation
3 Saint Petersburg Pasteur Institute, Saint-Petersburg, Russian Federation
4 Yale School of Public Health and Center for Interdisciplinary Research on AIDSYale University, New-Haven, United States
§ Presenting author email: ksenia@ngostellit.ru
Electronic publication date: 2012 Oct 22
Publication date: 2012
Volume: 15
Issue: Suppl 3
Electronic Location ID: 18440-004
Copyright: © 2012 K. Eritsyan et al.
Copyright year: 2012
License: This is an open-access article distributed under the terms of the Creative Commons Attribution License, which permits unrestricted use, distribution, and reproduction in any medium, provided the original work is properly cited.
License URL: http://creativecommons.org/licenses/by/2.0/

JOURNAL INFORMATION
==============================
NLM Title Abbreviation: J Int AIDS Soc
Journal ID: J Int AIDS Soc
NLM Journal ID: 101478566
Journal ID: JIAS

Journal of the International AIDS Society

EISSN: 1758-2652
Publisher: Wiley

ARTICLE INFORMATION
==============================
Article ID: 18440-005
Subjects: C3 - Modelling HIV epidemics

TUAC0505: Decline in incidence of HIV and hepatitis C virus infection among injecting drug users in Amsterdam: evidence for harm reduction?

de Vos A.S. 1 §
van der Helm J.J. 2
Prins M. 2 3
Kretzschmar M.E.E. 4 5
1 University Medical Center UtrechtJulius Center, Utrecht, Netherlands
2 Public Health Service, Amsterdam, Netherlands
3 Academic Medical Center (AMC), Amsterdam, Netherlands
4 University Medical Center Utrecht, Utrecht, Netherlands
5 Centre for Infectious Disease Control, RIVM, Bilthoven, Netherlands
§ Presenting author email: avos4@umcutrecht.nl
Electronic publication date: 2012 Oct 22
Publication date: 2012
Volume: 15
Issue: Suppl 3
Electronic Location ID: 18440-005
Copyright: © 2012 A.S. de Vos et al.
Copyright year: 2012
License: This is an open-access article distributed under the terms of the Creative Commons Attribution License, which permits unrestricted use, distribution, and reproduction in any medium, provided the original work is properly cited.
License URL: http://creativecommons.org/licenses/by/2.0/

JOURNAL INFORMATION
==============================
NLM Title Abbreviation: J Int AIDS Soc
Journal ID: J Int AIDS Soc
NLM Journal ID: 101478566
Journal ID: JIAS

Journal of the International AIDS Society

EISSN: 1758-2652
Publisher: Wiley

ARTICLE INFORMATION
==============================
Article ID: 18440-006
Subjects: C3 - Modelling HIV epidemics

MOPDC0201: Ageing with HIV in sub-Saharan Africa

Hontelez J.A.C. 1 2 3 §
de Vlas S.J. 1
Baltussen R. 3
Bakker R. 1
Tanser F. 2
Lurie M.N. 4
Bärnighausen T. 2 5
1 Erasmus MC, University Medical Center Rotterdam, Public Health, Rotterdam, Netherlands
2 Africa Centre for Health and Population StudiesUniversity of KwaZulu-Natal, Mtubatuba, South Africa
3 Radboud University Nijmegen Medical Center, Nijmegen International Center for Health Systems Research and Education (NICHE), Nijmegen, Netherlands
4 Brown University Warren Alpert School of Medicine, Providence, United States
5 Harvard School of Public Health, Department of Global Health and Population, Boston, United States
§ Presenting author email: j.hontelez@erasmusmc.nl
Electronic publication date: 2012 Oct 22
Publication date: 2012
Volume: 15
Issue: Suppl 3
Electronic Location ID: 18440-006
Copyright: © 2012 J.A.C. Hontelez et al.
Copyright year: 2012
License: This is an open-access article distributed under the terms of the Creative Commons Attribution License, which permits unrestricted use, distribution, and reproduction in any medium, provided the original work is properly cited.
License URL: http://creativecommons.org/licenses/by/2.0/

JOURNAL INFORMATION
==============================
NLM Title Abbreviation: J Int AIDS Soc
Journal ID: J Int AIDS Soc
NLM Journal ID: 101478566
Journal ID: JIAS

Journal of the International AIDS Society

EISSN: 1758-2652
Publisher: Wiley

ARTICLE INFORMATION
==============================
Article ID: 18440-007
Subjects: C3 - Modelling HIV epidemics

TUPDC0204: The contribution of HIV-discordant couples to HIV transmission in Lilongwe, Malawi

Powers K. 1 §
Ghani A. 2
Miller W. 1
Hoffman I. 1
Pettifor A. 1
Hosseinipour M. 1 3
Kamanga G. 3
Martinson F. 3
Cohen M. 1
1 University of North Carolina at Chapel Hill, Chapel Hill, United States
2 Imperial College London, London, United Kingdom
3 UNC Project Malawi, Lilongwe, Malawi
§ Presenting author email: powersk@email.unc.edu
Electronic publication date: 2012 Oct 22
Publication date: 2012
Volume: 15
Issue: Suppl 3
Electronic Location ID: 18440-007
Copyright: © 2012 K. Powers et al.
Copyright year: 2012
License: This is an open-access article distributed under the terms of the Creative Commons Attribution License, which permits unrestricted use, distribution, and reproduction in any medium, provided the original work is properly cited.
License URL: http://creativecommons.org/licenses/by/2.0/

JOURNAL INFORMATION
==============================
NLM Title Abbreviation: J Int AIDS Soc
Journal ID: J Int AIDS Soc
NLM Journal ID: 101478566
Journal ID: JIAS

Journal of the International AIDS Society

EISSN: 1758-2652
Publisher: Wiley

ARTICLE INFORMATION
==============================
Article ID: 18440-008
Subjects: C3 - Modelling HIV epidemics

WEPDC0106: Population-level benefits from providing effective HIV prevention means to pregnant women in high prevalence settings

Dimitrov D. 1 §
Boily M.-C. 2
Marrazzo J. 3
Brown E. 1
1 Fred Hutchinson Cancer Research Center, Vaccine & Infectious Disease Division, Seattle, United States
2 Imperial College London, Department of Infectious Disease Epidemiology, London, United Kingdom
3 University of Washington, Division of Allergy & Infectious Diseases, Seattle, United States
§ Presenting author email: dobromir@scharp.org
Electronic publication date: 2012 Oct 22
Publication date: 2012
Volume: 15
Issue: Suppl 3
Electronic Location ID: 18440-008
Copyright: © 2012 D. Dimitrov et al.
Copyright year: 2012
License: This is an open-access article distributed under the terms of the Creative Commons Attribution License, which permits unrestricted use, distribution, and reproduction in any medium, provided the original work is properly cited.
License URL: http://creativecommons.org/licenses/by/2.0/

JOURNAL INFORMATION
==============================
NLM Title Abbreviation: J Int AIDS Soc
Journal ID: J Int AIDS Soc
NLM Journal ID: 101478566
Journal ID: JIAS

Journal of the International AIDS Society

EISSN: 1758-2652
Publisher: Wiley

ARTICLE INFORMATION
==============================
Article ID: 18440-009
Subjects: C3 - Modelling HIV epidemics

THPDC0102: Modeling of HIV epidemic among men who have sex with men in Beijing, China

Qian H.-Z. 1 §
Lou J. 2
Ruan Y. 3
Webb G.F. 4
Shao Y. 3
Vermund S. 1
1 Vanderbilt Institute for Global Health, Nashville, United States
2 Shanghai University, Department of Mathematics, Shanghai, China
3 National Center for AIDS/STD Control and Prevention, China CDC, Division of Virology and Immunology, Beijing, China
4 Vanderbilt University, Department of Mathematics, Nashville, United States
§ Presenting author email: han-zhu.qian@vanderbilt.edu
Electronic publication date: 2012 Oct 22
Publication date: 2012
Volume: 15
Issue: Suppl 3
Electronic Location ID: 18440-009
Copyright: © 2012 H.-Z. Qian et al.
Copyright year: 2012
License: This is an open-access article distributed under the terms of the Creative Commons Attribution License, which permits unrestricted use, distribution, and reproduction in any medium, provided the original work is properly cited.
License URL: http://creativecommons.org/licenses/by/2.0/

JOURNAL INFORMATION
==============================
NLM Title Abbreviation: J Int AIDS Soc
Journal ID: J Int AIDS Soc
NLM Journal ID: 101478566
Journal ID: JIAS

Journal of the International AIDS Society

EISSN: 1758-2652
Publisher: Wiley

ARTICLE INFORMATION
==============================
Article ID: 18440-010
Subjects: C4 - Risk factors for acquisition of HIV

MOAC0404: Risk profiles of injecting partnerships: correlates of receptive syringe and cooker sharing among a cohort of young injecting drug users in San Francisco, California

Morris M. 1
Evans J. 1
Yu M. 1
Briceno A. 1
Page K. 1
Hahn J. 2
1 University of California at San Francisco, Epidemiology and Biostatistics, San Francisco, United States
2 University of California at San Francisco, Department of Medicine, San Francisco, United States
§
Electronic publication date: 2012 Oct 22
Publication date: 2012
Volume: 15
Issue: Suppl 3
Electronic Location ID: 18440-010
Copyright: © 2012 M. Morris et al.
Copyright year: 2012
License: This is an open-access article distributed under the terms of the Creative Commons Attribution License, which permits unrestricted use, distribution, and reproduction in any medium, provided the original work is properly cited.
License URL: http://creativecommons.org/licenses/by/2.0/

JOURNAL INFORMATION
==============================
NLM Title Abbreviation: J Int AIDS Soc
Journal ID: J Int AIDS Soc
NLM Journal ID: 101478566
Journal ID: JIAS

Journal of the International AIDS Society

EISSN: 1758-2652
Publisher: Wiley

ARTICLE INFORMATION
==============================
Article ID: 18440-011
Subjects: C4 - Risk factors for acquisition of HIV

WEAC0101: Lost in transition: prevalence and correlates of HIV infection among young men and women surviving abduction and displacement in post-conflict northern Uganda

Patel S. 1 §
Schechter M.T. 1
Muyinda H. 2
Kiwanuka N. 3
Sewankambo N.K. 4
Spittal P.M. 1
1 University of British Columbia, School of Population and Public Health, Vancouver, Canada
2 Makerere University, Child Health and Development Centre, Kampala, Uganda
3 Makerere University, School of Public Health, Kampala, Uganda
4 Makerere College of Health Sciences, Office of the Principal, Kampala, Uganda
§ Presenting author email: spatel55@hotmail.com
Electronic publication date: 2012 Oct 22
Publication date: 2012
Volume: 15
Issue: Suppl 3
Electronic Location ID: 18440-011
Copyright: © 2012 S. Patel et al.
Copyright year: 2012
License: This is an open-access article distributed under the terms of the Creative Commons Attribution License, which permits unrestricted use, distribution, and reproduction in any medium, provided the original work is properly cited.
License URL: http://creativecommons.org/licenses/by/2.0/

JOURNAL INFORMATION
==============================
NLM Title Abbreviation: J Int AIDS Soc
Journal ID: J Int AIDS Soc
NLM Journal ID: 101478566
Journal ID: JIAS

Journal of the International AIDS Society

EISSN: 1758-2652
Publisher: Wiley

ARTICLE INFORMATION
==============================
Article ID: 18440-012
Subjects: C5 - Hormonal contraception and HIV

WEAC0201: Association between STI/RTI infections, altered cervical innate immunity and HIV-1 seroconversion among hormonal contraceptive users

Fichorova R. 1
Morrison C. 2
Doncel G. 3
Chen P.-L. 2
Kwok C. 2
Chipato T. 4
Salata R. 5
Mauck C. 6
1 Brigham and Women's Hospital, Harvard Medical School, Boston, United States
2 Family Health International (FHI 360), Durham, United States
3 CONRAD, Eastern Virginia Medical School, Norfolk, United States
4 University of Zimbabwe, Harare, Zimbabwe
5 Case Western Reserve University, Cleveland, United States
6 CONRAD, Eastern Virginia Medical School, Arlington, United States
Electronic publication date: 2012 Oct 22
Publication date: 2012
Volume: 15
Issue: Suppl 3
Electronic Location ID: 18440-012
Copyright: © 2012 et al.
Copyright year: 2012
License: This is an open-access article distributed under the terms of the Creative Commons Attribution License, which permits unrestricted use, distribution, and reproduction in any medium, provided the original work is properly cited.
License URL: http://creativecommons.org/licenses/by/2.0/

JOURNAL INFORMATION
==============================
NLM Title Abbreviation: J Int AIDS Soc
Journal ID: J Int AIDS Soc
NLM Journal ID: 101478566
Journal ID: JIAS

Journal of the International AIDS Society

EISSN: 1758-2652
Publisher: Wiley

ARTICLE INFORMATION
==============================
Article ID: 18440-013
Subjects: C5 - Hormonal contraception and HIV

WEAC0202: Association of injectable contraception and risk of HIV-1 acquisition in women in HIV-1 serodiscordant partnerships: persistence of effect in multiple sensitivity analyses

Heffron R. 1 §
Donnell D. 2
Rees H. 3
Celum C. 1
Mugo N. 1 4
Were E. 5
de Bruyn G. 3
Nakku-Joloba E. 6
Ngure K. 4
Kiarie J. 1 4
Coombs R. 1
Baeten J. 1
Partners in Prevention HSV/HIV Transmission Study Team
1 University of Washington, Seattle, United States
2 Fred Hutchinson Cancer Research Center, Seattle, United States
3 University of Witswatersrand, Johannesburg, South Africa
4 Kenyatta National Hospital, Nairobi, Kenya
5 Moi University, Eldoret, Kenya
6 Makerere College of Health Sciences, Kampala, Uganda
§ Presenting author email: rheffron@u.washington.edu
Electronic publication date: 2012 Oct 22
Publication date: 2012
Volume: 15
Issue: Suppl 3
Electronic Location ID: 18440-013
Copyright: © 2012 R. Heffron et al.
Copyright year: 2012
License: This is an open-access article distributed under the terms of the Creative Commons Attribution License, which permits unrestricted use, distribution, and reproduction in any medium, provided the original work is properly cited.
License URL: http://creativecommons.org/licenses/by/2.0/

JOURNAL INFORMATION
==============================
NLM Title Abbreviation: J Int AIDS Soc
Journal ID: J Int AIDS Soc
NLM Journal ID: 101478566
Journal ID: JIAS

Journal of the International AIDS Society

EISSN: 1758-2652
Publisher: Wiley

ARTICLE INFORMATION
==============================
Article ID: 18440-014
Subjects: C5 - Hormonal contraception and HIV

WEAC0203: Hormonal contraception and HIV acquisition in women: a systematic review of the epidemiological evidence

Polis C. 1 §
Curtis K. 2
1 USAID, Office of Population and Reproductive Health, Washington, United States
2 Centers for Disease Control and Prevention, Division of Reproductive Health, Atlanta, United States
§ Presenting author email: cpolis@usaid.gov
Electronic publication date: 2012 Oct 22
Publication date: 2012
Volume: 15
Issue: Suppl 3
Electronic Location ID: 18440-014
Copyright: © 2012 C. Polis et al.
Copyright year: 2012
License: This is an open-access article distributed under the terms of the Creative Commons Attribution License, which permits unrestricted use, distribution, and reproduction in any medium, provided the original work is properly cited.
License URL: http://creativecommons.org/licenses/by/2.0/

JOURNAL INFORMATION
==============================
NLM Title Abbreviation: J Int AIDS Soc
Journal ID: J Int AIDS Soc
NLM Journal ID: 101478566
Journal ID: JIAS

Journal of the International AIDS Society

EISSN: 1758-2652
Publisher: Wiley

ARTICLE INFORMATION
==============================
Article ID: 18440-015
Subjects: C5 - Hormonal contraception and HIV

WEAC0204: Hormonal contraception and HIV disease progression: a systematic review of the epidemiological evidence

Phillips S. 1 §
Curtis K. 2
Polis C. 3
1 World Health Organization, Reproductive Health and Research, Geneva, Switzerland
2 Centers for Disease Control & Prevention, Division of Reproductive Health, Atlanta, United States
3 US Agency for International Development, Office of Population and Reproductive Health, Washington, United States
§ Presenting author email: phillipss@who.int
Electronic publication date: 2012 Oct 22
Publication date: 2012
Volume: 15
Issue: Suppl 3
Electronic Location ID: 18440-015
Copyright: © 2012 S. Phillips et al.
Copyright year: 2012
License: This is an open-access article distributed under the terms of the Creative Commons Attribution License, which permits unrestricted use, distribution, and reproduction in any medium, provided the original work is properly cited.
License URL: http://creativecommons.org/licenses/by/2.0/

JOURNAL INFORMATION
==============================
NLM Title Abbreviation: J Int AIDS Soc
Journal ID: J Int AIDS Soc
NLM Journal ID: 101478566
Journal ID: JIAS

Journal of the International AIDS Society

EISSN: 1758-2652
Publisher: Wiley

ARTICLE INFORMATION
==============================
Article ID: 18440-016
Subjects: C6 - Risk factors for infectivity, progression and transmission of HIV

WEAB0202: Racial disparities in antiretroviral therapy use and viral suppression among sexually active HIV-positive men who have sex with men receiving medical care: United States, Medical Monitoring Project, 2009 data collection cycle

Beer L. 1 §
Oster A. 1
Mattson C. 1
Skarbinski J. 1
1 U.S. Centers for Disease Control and Prevention, Atlanta, United States
§ Presenting author email: lbeer@cdc.gov
Electronic publication date: 2012 Oct 22
Publication date: 2012
Volume: 15
Issue: Suppl 3
Electronic Location ID: 18440-016
Copyright: © 2012 L. Beer et al.
Copyright year: 2012
License: This is an open-access article distributed under the terms of the Creative Commons Attribution License, which permits unrestricted use, distribution, and reproduction in any medium, provided the original work is properly cited.
License URL: http://creativecommons.org/licenses/by/2.0/

JOURNAL INFORMATION
==============================
NLM Title Abbreviation: J Int AIDS Soc
Journal ID: J Int AIDS Soc
NLM Journal ID: 101478566
Journal ID: JIAS

Journal of the International AIDS Society

EISSN: 1758-2652
Publisher: Wiley

ARTICLE INFORMATION
==============================
Article ID: 18440-017
Subjects: C6 - Risk factors for infectivity, progression and transmission of HIV

TUAC0101: HIV-1 subtype C is not associated with higher risk of heterosexual transmission: a multinational study among African HIV-1 serodiscordant couples

Kahle E. 1 §
Campbell M. 1
Lingappa J. 1
Donnell D. 2
Celum C. 1
Kapiga S. 3
Ondondo R. 4
Mugo N. 1 5
Mujugira A. 1
Fife K. 6
Mullins J. 1
Baeten J. 1
Partners in Prevention HSV/HIV Transmission Study
1 University of Washington, Seattle, United States
2 Fred Hutchinson Cancer Research Center, Seattle, United States
3 London School of Hygiene and Tropical Medicine, London, United Kingdom
4 Kenya Medical Research Institute, Kisumu, Kenya
5 Kenyatta National Hospital, Nairobi, Kenya
6 Indiana University, Bloomington, United States
§ Presenting author email: ekahle@u.washington.edu
Electronic publication date: 2012 Oct 22
Publication date: 2012
Volume: 15
Issue: Suppl 3
Electronic Location ID: 18440-017
Copyright: © 2012 E. Kahle et al.
Copyright year: 2012
License: This is an open-access article distributed under the terms of the Creative Commons Attribution License, which permits unrestricted use, distribution, and reproduction in any medium, provided the original work is properly cited.
License URL: http://creativecommons.org/licenses/by/2.0/

JOURNAL INFORMATION
==============================
NLM Title Abbreviation: J Int AIDS Soc
Journal ID: J Int AIDS Soc
NLM Journal ID: 101478566
Journal ID: JIAS

Journal of the International AIDS Society

EISSN: 1758-2652
Publisher: Wiley

ARTICLE INFORMATION
==============================
Article ID: 18440-018
Subjects: C6 - Risk factors for infectivity, progression and transmission of HIV

TUAC0104: Impact of antiretroviral therapy on HIV-positive status disclosure in rural South Africa

Bearnot B. 1 2 §
Werner L. 1
Kharsany A. 1
Abdool Karim S. 1 3
Frohlich J. 1
Abdool Karim Q. 1 3
1 CAPRISA, UKZN, Durban, South Africa
2 New York University, New York, United States
3 Columbia University, New York, United States
§ Presenting author email: bibearnot@gmail.com
Electronic publication date: 2012 Oct 22
Publication date: 2012
Volume: 15
Issue: Suppl 3
Electronic Location ID: 18440-018
Copyright: © 2012 B. Bearnot et al.
Copyright year: 2012
License: This is an open-access article distributed under the terms of the Creative Commons Attribution License, which permits unrestricted use, distribution, and reproduction in any medium, provided the original work is properly cited.
License URL: http://creativecommons.org/licenses/by/2.0/

JOURNAL INFORMATION
==============================
NLM Title Abbreviation: J Int AIDS Soc
Journal ID: J Int AIDS Soc
NLM Journal ID: 101478566
Journal ID: JIAS

Journal of the International AIDS Society

EISSN: 1758-2652
Publisher: Wiley

ARTICLE INFORMATION
==============================
Article ID: 18440-019
Subjects: C6 - Risk factors for infectivity, progression and transmission of HIV

TUAC0202: More frequent sexual risk behaviours among HIV-negative sexually transmitted infection clinic patients compared to HIV-positive and HIV-negative HIV testing and counseling center patients in Lilongwe, Malawi

Mapanje C. 1
Rutstein S. 2 3 §
Kamanga G. 1 3
Phiri S. 4
Nsona D. 4
Pettifor A. 5
Rosenberg N. 5
Hoffman I. 2
Miller W. 2 5
1 UNC Project-Malawi, Lilongwe, Malawi
2 University of North Carolina, Department of Medicine, Chapel Hill, United States
3 University of North Carolina, Department of Health Policy and Management, Chapel Hill, United States
4 Lighthouse Trust, Lilongwe, Malawi
5 University of North Carolina, Department of Epidemiology, Chapel Hill, United States
§ Presenting author email: sarah_rutstein@med.unc.edu
Electronic publication date: 2012 Oct 22
Publication date: 2012
Volume: 15
Issue: Suppl 3
Electronic Location ID: 18440-019
Copyright: © 2012 S. Rutstein et al.
Copyright year: 2012
License: This is an open-access article distributed under the terms of the Creative Commons Attribution License, which permits unrestricted use, distribution, and reproduction in any medium, provided the original work is properly cited.
License URL: http://creativecommons.org/licenses/by/2.0/

JOURNAL INFORMATION
==============================
NLM Title Abbreviation: J Int AIDS Soc
Journal ID: J Int AIDS Soc
NLM Journal ID: 101478566
Journal ID: JIAS

Journal of the International AIDS Society

EISSN: 1758-2652
Publisher: Wiley

ARTICLE INFORMATION
==============================
Article ID: 18440-020
Subjects: C6 - Risk factors for infectivity, progression and transmission of HIV

THAC0202: Patients in routine HIV clinical care at-risk for potentially transmitting HIV in the ‘test-and-treat’ era of HIV prevention

Crane H. 1 §
Mimiaga M. 2
Feldman B. 1
Fredericksen R. 1
Mugavero M. 3
Willig J. 3
Safren S. 4
Matthews W. 5
Christopoulos K. 6
Boswell S. 7
Kitahata M. 8
Saag M. 3
Mayer K. 7
Centers for AIDS Research Network of Integrated Clinical Systems (CNICS)
1 University of Washington, Seattle, Medicine, United States
2 The Fenway Institute and Harvard Medical School, Boston, United States
3 University of Alabama at Birmingham School of Medicine, Birmingham, United States
4 Harvard Medical School, Boston, United States
5 University of California-San Diego (UCSD), San Diego, United States
6 University of California-San Francisco, San Francisco, United States
7 Fenway Institute, Boston, United States
8 University of Washington, Seattle, United States
§ Presenting author email: hcrane@uw.edu
Electronic publication date: 2012 Oct 22
Publication date: 2012
Volume: 15
Issue: Suppl 3
Electronic Location ID: 18440-020
Copyright: © 2012 H. Crane et al.
Copyright year: 2012
License: This is an open-access article distributed under the terms of the Creative Commons Attribution License, which permits unrestricted use, distribution, and reproduction in any medium, provided the original work is properly cited.
License URL: http://creativecommons.org/licenses/by/2.0/

JOURNAL INFORMATION
==============================
NLM Title Abbreviation: J Int AIDS Soc
Journal ID: J Int AIDS Soc
NLM Journal ID: 101478566
Journal ID: JIAS

Journal of the International AIDS Society

EISSN: 1758-2652
Publisher: Wiley

ARTICLE INFORMATION
==============================
Article ID: 18440-021
Subjects: C6 - Risk factors for infectivity, progression and transmission of HIV

FRLBC04: BCG vaccination at birth induces non-specific CD4 T cell activation in HIV-exposed South African infants, which may increase HIV transmission through breastfeeding

Hesseling A.K. 1
Passmore J.-A.S. 2
Gamieldien H. 2
Jones C.E. 3
Hanekom W. 4
Sodora D.L. 5
Jaspan H.B. 2 5 §
1 Stellenbosch University, Desmond Tutu TB Centre, Cape Town, South Africa
2 University of Cape Town, Institute of Infectious Diseases and Molecular Medicine, Clinical Lab Sciences, Cape Town, South Africa
3 Imperial College London, Academic Department of Pediatrics, London, United Kingdom
4 University of Cape Town, Institute of Infectious Diseases and Molecular Medicine, South African TB Vaccine Institute, Cape Town, South Africa
5 Seattle Biomedical Research Institute, Seattle, United States
§ Presenting author email: hbjaspan@gmail.com
Electronic publication date: 2012 Oct 22
Publication date: 2012
Volume: 15
Issue: Suppl 3
Electronic Location ID: 18440-021
Copyright: © 2012 H.B. Jaspan et al.
Copyright year: 2012
License: This is an open-access article distributed under the terms of the Creative Commons Attribution License, which permits unrestricted use, distribution, and reproduction in any medium, provided the original work is properly cited.
License URL: http://creativecommons.org/licenses/by/2.0/

JOURNAL INFORMATION
==============================
NLM Title Abbreviation: J Int AIDS Soc
Journal ID: J Int AIDS Soc
NLM Journal ID: 101478566
Journal ID: JIAS

Journal of the International AIDS Society

EISSN: 1758-2652
Publisher: Wiley

ARTICLE INFORMATION
==============================
Article ID: 18440-022
Subjects: C6 - Risk factors for infectivity, progression and transmission of HIV

FRLBX05: Continuum of HIV care: differences in care and treatment by sex and race/ethnicity in the United States

Hall H.I. 1 §
Frazier E.L. 1
Rhodes P. 1
Holtgrave D.R. 2
Furlow-Parmley C. 1
Tang T. 3
Gray K.M. 1
Cohen S.M. 1
Skarbinski J. 1
1 Centers for Disease Control and Prevention, Atlanta, United States
2 Johns Hopkins Bloomberg School of Public Health, Baltimore, United States
3 ICF International, Atlanta, United States
§ Presenting author email: ixh1@cdc.gov
Electronic publication date: 2012 Oct 22
Publication date: 2012
Volume: 15
Issue: Suppl 3
Electronic Location ID: 18440-022
Copyright: © 2012 H.I. Hall et al.
Copyright year: 2012
License: This is an open-access article distributed under the terms of the Creative Commons Attribution License, which permits unrestricted use, distribution, and reproduction in any medium, provided the original work is properly cited.
License URL: http://creativecommons.org/licenses/by/2.0/

JOURNAL INFORMATION
==============================
NLM Title Abbreviation: J Int AIDS Soc
Journal ID: J Int AIDS Soc
NLM Journal ID: 101478566
Journal ID: JIAS

Journal of the International AIDS Society

EISSN: 1758-2652
Publisher: Wiley

ARTICLE INFORMATION
==============================
Article ID: 18440-023
Subjects: C6 - Risk factors for infectivity, progression and transmission of HIV

TUPDC0201: One in ten couples in Mozambique is serodiscordant and most do not know it: results from the 2009 national HIV seroprevalence survey

Fishel J.D. 1
Bradley S.E. 1
Young P.W. 2
Mbofana F. 3
Botao C. 3
1 ICF International, Calverton, United States
2 CDC-Mozambique, Maputo, Mozambique
3 National Institute of Health, Maputo, Mozambique
§
Electronic publication date: 2012 Oct 22
Publication date: 2012
Volume: 15
Issue: Suppl 3
Electronic Location ID: 18440-023
Copyright: © 2012 S.E. Bradley et al.
Copyright year: 2012
License: This is an open-access article distributed under the terms of the Creative Commons Attribution License, which permits unrestricted use, distribution, and reproduction in any medium, provided the original work is properly cited.
License URL: http://creativecommons.org/licenses/by/2.0/

JOURNAL INFORMATION
==============================
NLM Title Abbreviation: J Int AIDS Soc
Journal ID: J Int AIDS Soc
NLM Journal ID: 101478566
Journal ID: JIAS

Journal of the International AIDS Society

EISSN: 1758-2652
Publisher: Wiley

ARTICLE INFORMATION
==============================
Article ID: 18440-024
Subjects: C7 - Epidemiology of HIV in the general population, women, adolescents and children

FRLBX01: HIV prevalence trends after scale-up of antiretroviral treatment: a population-based study in a poor rural community in KwaZulu-Natal

Zaidi J. 1 §
Grapsa E. 1
Tanser F. 1
Newell M.-L. 1 2
Bärnighausen T. 1 3
1 Africa Centre for Health and Population Studies, University of KwaZulu-Natal, Mtubatuba, South Africa
2 MRC Centre for Epidemiology of Child Health, University College London Institute of Child Health, London, United Kingdom
3 Harvard School of Public Health, Global Health and Population, Boston, United States
§ Presenting author email: jaffer_mz@hotmail.com
Electronic publication date: 2012 Oct 22
Publication date: 2012
Volume: 15
Issue: Suppl 3
Electronic Location ID: 18440-024
Copyright: © 2012 J. Zaidi et al.
Copyright year: 2012
License: This is an open-access article distributed under the terms of the Creative Commons Attribution License, which permits unrestricted use, distribution, and reproduction in any medium, provided the original work is properly cited.
License URL: http://creativecommons.org/licenses/by/2.0/

JOURNAL INFORMATION
==============================
NLM Title Abbreviation: J Int AIDS Soc
Journal ID: J Int AIDS Soc
NLM Journal ID: 101478566
Journal ID: JIAS

Journal of the International AIDS Society

EISSN: 1758-2652
Publisher: Wiley

ARTICLE INFORMATION
==============================
Article ID: 18440-025
Subjects: C7 - Epidemiology of HIV in the general population, women, adolescents and children

MOPDC0202: The urban HIV epidemic in eastern and southern Africa: need for better KYE/KYR to inform adequate city responses

Van Renterghem H. 1 §
Colvin M. 2
de Beer I. 3
Gunthorp J. 4
Odiit M. 1
Thomas L. 5
Jackson H. 6
Getahun M. 7
1 UNAIDS, Windhoek, Namibia
2 Maromi Health Research, Cape Town, South Africa
3 PharmAccess Foundation, Windhoek, Namibia
4 Southern African AIDS TRUST, Johannesburg, South Africa
5 Wits University, Johannesburg, South Africa
6 UNAIDS, Johannesburg, South Africa
7 UNDP, Johannesburg, South Africa
§ Presenting author email: vanrenterghemh@unaids.org
Electronic publication date: 2012 Oct 22
Publication date: 2012
Volume: 15
Issue: Suppl 3
Electronic Location ID: 18440-025
Copyright: © 2012 H. Van Renterghem et al.
Copyright year: 2012
License: This is an open-access article distributed under the terms of the Creative Commons Attribution License, which permits unrestricted use, distribution, and reproduction in any medium, provided the original work is properly cited.
License URL: http://creativecommons.org/licenses/by/2.0/

JOURNAL INFORMATION
==============================
NLM Title Abbreviation: J Int AIDS Soc
Journal ID: J Int AIDS Soc
NLM Journal ID: 101478566
Journal ID: JIAS

Journal of the International AIDS Society

EISSN: 1758-2652
Publisher: Wiley

ARTICLE INFORMATION
==============================
Article ID: 18440-026
Subjects: C8 - Epidemiology of HIV in people who use drugs

MOAC0401: Low risk of HIV and STI among drug users in Amsterdam

Grady B.P.X. 1 §
van der Knaap N. 2
Schim van der Loeff M. 1
Heijman T. 1
Speksnijder A. 3
Geskus R. 4
Prins M. 1 5
1 Public Health Service Amsterdam, Infectious Diseases, Research, Amsterdam, Netherlands
2 University of Amsterdam, Amsterdam, Netherlands
3 Public Health Service Amsterdam, Laboratory of Public Health, Amsterdam, Netherlands
4 Public Health Service Amsterdam, Laboratory of Public Health, Amsterdam, Netherlands
5 Academic Medical Center (AMC), Internal Medicine, Infectious Diseases, Tropical Medicine and AIDS, Amsterdam, Netherlands
§ Presenting author email: bgrady@ggd.amsterdam.nl
Electronic publication date: 2012 Oct 22
Publication date: 2012
Volume: 15
Issue: Suppl 3
Electronic Location ID: 18440-026
Copyright: © 2012 B.P.X. Grady et al.
Copyright year: 2012
License: This is an open-access article distributed under the terms of the Creative Commons Attribution License, which permits unrestricted use, distribution, and reproduction in any medium, provided the original work is properly cited.
License URL: http://creativecommons.org/licenses/by/2.0/

JOURNAL INFORMATION
==============================
NLM Title Abbreviation: J Int AIDS Soc
Journal ID: J Int AIDS Soc
NLM Journal ID: 101478566
Journal ID: JIAS

Journal of the International AIDS Society

EISSN: 1758-2652
Publisher: Wiley

ARTICLE INFORMATION
==============================
Article ID: 18440-027
Subjects: C8 - Epidemiology of HIV in people who use drugs

MOAC0405: Methamphetamine use is associated with sexual abuse and HIV sexual risk behaviours among patrons of alcohol serving venues in Cape Town, South Africa

Meade C.S. 1 §
Watt M.H. 2
Sikkema K.J. 3
Deng L.X. 4
Ranby K.W. 4
Skinner D. 5
Pieterse D. 5
Kalichman S.C. 6
1 Duke University School of Medicine, Psychiatry & Behavioral Sciences, Durham, United States
2 Duke Global Health Institute, Durham, United States
3 Duke University, Psychology & Neuroscience, Durham, United States
4 Duke University, Durham, United States
5 Stellenbosch University, Cape Town, South Africa
6 University of Connecticut, Storrs, United States
§ Presenting author email: christina.meade@duke.edu
Electronic publication date: 2012 Oct 22
Publication date: 2012
Volume: 15
Issue: Suppl 3
Electronic Location ID: 18440-027
Copyright: © 2012 C.S. Meade et al.
Copyright year: 2012
License: This is an open-access article distributed under the terms of the Creative Commons Attribution License, which permits unrestricted use, distribution, and reproduction in any medium, provided the original work is properly cited.
License URL: http://creativecommons.org/licenses/by/2.0/

JOURNAL INFORMATION
==============================
NLM Title Abbreviation: J Int AIDS Soc
Journal ID: J Int AIDS Soc
NLM Journal ID: 101478566
Journal ID: JIAS

Journal of the International AIDS Society

EISSN: 1758-2652
Publisher: Wiley

ARTICLE INFORMATION
==============================
Article ID: 18440-028
Subjects: C8 - Epidemiology of HIV in people who use drugs

THAC0305: Concurrent drug use and sexual risk behaviour among men who have sex with men (MSM) in Peru

Peinado J. 1
Lama J.R. 1 §
Gonzales P. 1
Cabello R. 2
Sal y Rosas G. 1
Sanchez J. 1
1 Asociacion Civil Impacta Salud y Educacion, Lima, Peru
2 Asociacion Via Libre, Lima, Peru
§ Presenting author email: jrlama@impactaperu.org
Electronic publication date: 2012 Oct 22
Publication date: 2012
Volume: 15
Issue: Suppl 3
Electronic Location ID: 18440-028
Copyright: © 2012 J.R. Lama et al.
Copyright year: 2012
License: This is an open-access article distributed under the terms of the Creative Commons Attribution License, which permits unrestricted use, distribution, and reproduction in any medium, provided the original work is properly cited.
License URL: http://creativecommons.org/licenses/by/2.0/

JOURNAL INFORMATION
==============================
NLM Title Abbreviation: J Int AIDS Soc
Journal ID: J Int AIDS Soc
NLM Journal ID: 101478566
Journal ID: JIAS

Journal of the International AIDS Society

EISSN: 1758-2652
Publisher: Wiley

ARTICLE INFORMATION
==============================
Article ID: 18440-029
Subjects: C8 - Epidemiology of HIV in people who use drugs

THAC0403: Predictors of dropping out of methadone maintenance treatment in China: a six-year cohort study

Cao X. 1 §
Wu Z. 1
Rou K. 1
Pang L. 1
1 National Center for AIDS/STD Control and Prevention, Chinese Center for Disease Control and Prevention, Division of Health Eduction and Behavioral Intervention, Beijing, China
§ Presenting author email: xiaobincao@hotmail.com
Electronic publication date: 2012 Oct 22
Publication date: 2012
Volume: 15
Issue: Suppl 3
Electronic Location ID: 18440-029
Copyright: © 2012 X. Cao et al.
Copyright year: 2012
License: This is an open-access article distributed under the terms of the Creative Commons Attribution License, which permits unrestricted use, distribution, and reproduction in any medium, provided the original work is properly cited.
License URL: http://creativecommons.org/licenses/by/2.0/

JOURNAL INFORMATION
==============================
NLM Title Abbreviation: J Int AIDS Soc
Journal ID: J Int AIDS Soc
NLM Journal ID: 101478566
Journal ID: JIAS

Journal of the International AIDS Society

EISSN: 1758-2652
Publisher: Wiley

ARTICLE INFORMATION
==============================
Article ID: 18440-030
Subjects: C9 - Epidemiology of HIV in male and female sex workers

THAC0501: High and disproportionate burden of HIV among female sex workers in low- and middle-income countries: a systematic review and meta-analysis

Baral S. 1
Beyrer C. 1
Muessig K. 2
Poteat T. 1
Wirtz A. 1
Decker M. 3
Sherman S. 4
Kerrigan D. 5 §
1 Center for Public Health and Human Rights, JHSPH, Epidemiology, Baltimore, United States
2 Johns Hopkins School of Public Health, International Health, Baltimore, United States
3 Johns Hopkins Bloomberg School of Public Health, Population, Family, and Reproductive Health, Baltimore, United States
4 Johns Hopkins Bloomberg School of Public Health, Epidemiology, Baltimore, United States
5 Johns Hopkins Bloomberg School of Public Health, Health, Behavior, and Society, Baltimore, United States
§
Electronic publication date: 2012 Oct 22
Publication date: 2012
Volume: 15
Issue: Suppl 3
Electronic Location ID: 18440-030
Copyright: © 2012 S. Baral et al.
Copyright year: 2012
License: This is an open-access article distributed under the terms of the Creative Commons Attribution License, which permits unrestricted use, distribution, and reproduction in any medium, provided the original work is properly cited.
License URL: http://creativecommons.org/licenses/by/2.0/

JOURNAL INFORMATION
==============================
NLM Title Abbreviation: J Int AIDS Soc
Journal ID: J Int AIDS Soc
NLM Journal ID: 101478566
Journal ID: JIAS

Journal of the International AIDS Society

EISSN: 1758-2652
Publisher: Wiley

ARTICLE INFORMATION
==============================
Article ID: 18440-031
Subjects: C9 - Epidemiology of HIV in male and female sex workers

THPDE0201: Hyper-endemic HIV seroprevalence among young female sex workers in post-conflict, northern Uganda: a call for social and structural HIV interventions

Shannon K. 1 §
Akello M. 2
Muldoon K. 3
Simo A. 3
Muzaaya G. 2
1 British Columbia Centre for Excellence in HIV/AIDS University of British ColumbiaSchool of Population and Public Health, Faculty of Medicine, Vancouver, Canada
2 The AIDS Support Organisation-TASO Uganda, Gulu, Uganda
3 BC Centre for Excellence in HIV/AIDSUniversity of British Columbia, Vancouver, Canada
§ Presenting author email: kshannon@cfenet.ubc.ca
Electronic publication date: 2012 Oct 22
Publication date: 2012
Volume: 15
Issue: Suppl 3
Electronic Location ID: 18440-031
Copyright: © 2012 K. Shannon et al.
Copyright year: 2012
License: This is an open-access article distributed under the terms of the Creative Commons Attribution License, which permits unrestricted use, distribution, and reproduction in any medium, provided the original work is properly cited.
License URL: http://creativecommons.org/licenses/by/2.0/

JOURNAL INFORMATION
==============================
NLM Title Abbreviation: J Int AIDS Soc
Journal ID: J Int AIDS Soc
NLM Journal ID: 101478566
Journal ID: JIAS

Journal of the International AIDS Society

EISSN: 1758-2652
Publisher: Wiley

ARTICLE INFORMATION
==============================
Article ID: 18440-032
Subjects: C10 - Epidemiology of HIV in men having sex with men (MSM)

MOAC0101: Equal behaviors, unequal risks: the role of partner transmission potential in racial HIV disparities among men who have sex with men (MSM) in the US

Rosenberg E. 1 §
Kelley C. 2
O'Hara B. 1
Frew P. 2
Peterson J. 3
Sanchez T. 1
del Rio C. 4
Sullivan P. 1
1 Emory University Rollins School of Public Health, Epidemiology, Atlanta, United States
2 Emory University School of Medicine, Infectious Diseases, Atlanta, United States
3 Georgia State University, Psychology, Atlanta, United States
4 Emory University Rollins School of Public Health, Global Health, Atlanta, United States
§ Presenting author email: esrose2@emory.edu
Electronic publication date: 2012 Oct 22
Publication date: 2012
Volume: 15
Issue: Suppl 3
Electronic Location ID: 18440-032
Copyright: © 2012 E. Rosenberg et al.
Copyright year: 2012
License: This is an open-access article distributed under the terms of the Creative Commons Attribution License, which permits unrestricted use, distribution, and reproduction in any medium, provided the original work is properly cited.
License URL: http://creativecommons.org/licenses/by/2.0/

JOURNAL INFORMATION
==============================
NLM Title Abbreviation: J Int AIDS Soc
Journal ID: J Int AIDS Soc
NLM Journal ID: 101478566
Journal ID: JIAS

Journal of the International AIDS Society

EISSN: 1758-2652
Publisher: Wiley

ARTICLE INFORMATION
==============================
Article ID: 18440-033
Subjects: C10 - Epidemiology of HIV in men having sex with men (MSM)

MOAC0103: Foreign location of birth and time since immigration are associated with HIV status among Latino MSM in the United States

Oster A. 1 §
Russell K. 1
Wiegand R. 1
Le B. 1
Valverde E. 1
Forrest D. 2
Cribbin M. 1
Paz-Bailey G. 1
NHBS Study Group
1 Centers for Disease Control and Prevention, Division of HIV/AIDS Prevention, Atlanta, United States
2 University of Miami, Miami, United States
§ Presenting author email: aoster@cdc.gov
Electronic publication date: 2012 Oct 22
Publication date: 2012
Volume: 15
Issue: Suppl 3
Electronic Location ID: 18440-033
Copyright: © 2012 A. Oster et al.
Copyright year: 2012
License: This is an open-access article distributed under the terms of the Creative Commons Attribution License, which permits unrestricted use, distribution, and reproduction in any medium, provided the original work is properly cited.
License URL: http://creativecommons.org/licenses/by/2.0/

JOURNAL INFORMATION
==============================
NLM Title Abbreviation: J Int AIDS Soc
Journal ID: J Int AIDS Soc
NLM Journal ID: 101478566
Journal ID: JIAS

Journal of the International AIDS Society

EISSN: 1758-2652
Publisher: Wiley

ARTICLE INFORMATION
==============================
Article ID: 18440-034
Subjects: C10 - Epidemiology of HIV in men having sex with men (MSM)

MOAC0104: Trends in HIV prevalence and HIV testing among young MSM: five United States cities, 1994–2008

Oster A. 1 §
Johnson C. 1
Le B. 1
Finlayson T. 1
Balaji A. 1
Lansky A. 1
Mermin J. 1
Valleroy L. 1
MacKellar D. 1
Behel S. 1
Paz-Bailey G. 1
YMS and NHBS Study Groups 1
1 Centers for Disease Control and Prevention, Division of HIV/AIDS Prevention, Atlanta, United States
§ Presenting author email: aoster@cdc.gov
Electronic publication date: 2012 Oct 22
Publication date: 2012
Volume: 15
Issue: Suppl 3
Electronic Location ID: 18440-034
Copyright: © 2012 A. Oster et al.
Copyright year: 2012
License: This is an open-access article distributed under the terms of the Creative Commons Attribution License, which permits unrestricted use, distribution, and reproduction in any medium, provided the original work is properly cited.
License URL: http://creativecommons.org/licenses/by/2.0/

JOURNAL INFORMATION
==============================
NLM Title Abbreviation: J Int AIDS Soc
Journal ID: J Int AIDS Soc
NLM Journal ID: 101478566
Journal ID: JIAS

Journal of the International AIDS Society

EISSN: 1758-2652
Publisher: Wiley

ARTICLE INFORMATION
==============================
Article ID: 18440-035
Subjects: C10 - Epidemiology of HIV in men having sex with men (MSM)

MOAC0105: An evolving concentrated epidemic: comparison of socioeconomic, behavioural and biological factors among newly diagnosed, previously diagnosed and HIV-negative black men who have sex with men in six US cities (HPTN 061)

Mayer K. 1 §
Wang L. 2
Koblin B. 3
Mao C. 2
Mannheimer S. 4
Magnus M. 5
del Rio C. 6
Buchbinder S. 7
Wilton L. 8
Cummings V. 9
Watson C. 5
Griffith S. 10
Wheeler D. 11
HPTN 061 Protocol Team
1 The Fenway Institute/Harvard Medical School, Boston, United States
2 Statistical Center for HIV/AIDS Research & Prevention (SCHARP), Seattle, United States
3 New York Blood Center, New York, United States
4 Harlem Hospital/Columbia University, Department of Medicine/Mailman School of Public Health, New York, United States
5 The George Washington University, Department of Epidemiology and Biostatistics, Washington, United States
6 Emory University Rollins School of Public Health, Department of Epidemiology, Atlanta, United States
7 San Francisco Department of Health, HIV Research Division, San Francisco, United States
8 Binghamton University, Department of Human Development, Binghamton, United States
9 Johns Hopkins University School of Medicine, Pathology Department, Baltimore, United States
10 FHI 360, Durham, United States
11 HPTN 061 Protocol Team, New York, United States
§ Presenting author email: kmayer@fenwayhealth.org
Electronic publication date: 2012 Oct 22
Publication date: 2012
Volume: 15
Issue: Suppl 3
Electronic Location ID: 18440-035
Copyright: © 2012 K. Mayer et al.
Copyright year: 2012
License: This is an open-access article distributed under the terms of the Creative Commons Attribution License, which permits unrestricted use, distribution, and reproduction in any medium, provided the original work is properly cited.
License URL: http://creativecommons.org/licenses/by/2.0/

JOURNAL INFORMATION
==============================
NLM Title Abbreviation: J Int AIDS Soc
Journal ID: J Int AIDS Soc
NLM Journal ID: 101478566
Journal ID: JIAS

Journal of the International AIDS Society

EISSN: 1758-2652
Publisher: Wiley

ARTICLE INFORMATION
==============================
Article ID: 18440-036
Subjects: C10 - Epidemiology of HIV in men having sex with men (MSM)

MOAC0106: Correlates of HIV incidence among black men who have sex with men in 6 U.S. cities (HPTN 061)

Koblin B. 1 §
Mayer K. 2
Eshleman S. 3
Wang L. 4
Shoptaw S. 5
del Rio C. 6
Buchbinder S. 7
Magnus M. 8
Mannheimer S. 9 10
Liu T.-Y. 4
Cummings V. 3
Piwowar-Manning E. 3
Fields S. 11
Griffith S. 12
Elharrar V. 13
Wheeler D. 14
HPTN 061 Study Team
1 New York Blood Center, Laboratory of Infectious Disease Prevention, New York, United States
2 Fenway Health and Beth Israel Deaconess Hospital, Boston, United States
3 Johns Hopkins University School of Medicine, Department of Pathology, Baltimore, United States
4 Fred Hutchinson Cancer Research Center, Vaccine and Infectious Disease Division, Seattle, United States
5 University of California, Department of Family Medicine, Los Angeles, United States
6 Emory University Rollins School of Public Health, Department of Global Health, Atlanta, United States
7 San Francisco Department of Public Health, San Francisco, United States
8 George Washington University, School of Public Health and Health Services, Department of Epidemiology and Biostatistics, Washington DC, United States
9 Harlem Hospital/ Columbia University, Department of Medicine, New York, United States
10 Columbia University Mailman School of Public Health, Department of Epidemiology, New York, United States
11 Florida International University-College of Nursing and Health Sciences, Miami
12 FHI 360, Research Triangle Park, United States
13 NIAID, National Institutes of HealthClinical Prevention Research Branch, Bethesda, United States
14 Loyola University ChicagoGraduate School of Social Work, Chicago, United States
§ Presenting author email: bkoblin@nybloodcenter.org
Electronic publication date: 2012 Oct 22
Publication date: 2012
Volume: 15
Issue: Suppl 3
Electronic Location ID: 18440-036
Copyright: © 2012 B. Koblin et al.
Copyright year: 2012
License: This is an open-access article distributed under the terms of the Creative Commons Attribution License, which permits unrestricted use, distribution, and reproduction in any medium, provided the original work is properly cited.
License URL: http://creativecommons.org/licenses/by/2.0/

JOURNAL INFORMATION
==============================
NLM Title Abbreviation: J Int AIDS Soc
Journal ID: J Int AIDS Soc
NLM Journal ID: 101478566
Journal ID: JIAS

Journal of the International AIDS Society

EISSN: 1758-2652
Publisher: Wiley

ARTICLE INFORMATION
==============================
Article ID: 18440-037
Subjects: C10 - Epidemiology of HIV in men having sex with men (MSM)

THAC0302: Men who have sex with men and the HIV epidemic in Morocco: results from a respondent-driven sampling study

Mellouk O. 1 2 §
Alami K. 3
El Rhilani H. 3
Rafif N. 1
Abadie A. 1
Ouarsas L. 4
Bennani A. 5
El Omari B. 6
Oumzil I. 7
Johnston L. 8
1 ALCS, Marrakech, Morocco
2 ITPC North Africa, Marrakech, Morocco
3 UNAIDS Morocco, Rabat, Morocco
4 ALCS, Agadir, Morocco
5 PNLS, Rabat, Morocco
6 Fonds Mondial, Rabat, Morocco
7 INH, Rabat, Morocco
8 UNAIDS, Amsterdam, Netherlands
§ Presenting author email: o.mellouk@gmail.com
Electronic publication date: 2012 Oct 22
Publication date: 2012
Volume: 15
Issue: Suppl 3
Electronic Location ID: 18440-037
Copyright: © 2012 O. Mellouk et al.
Copyright year: 2012
License: This is an open-access article distributed under the terms of the Creative Commons Attribution License, which permits unrestricted use, distribution, and reproduction in any medium, provided the original work is properly cited.
License URL: http://creativecommons.org/licenses/by/2.0/

JOURNAL INFORMATION
==============================
NLM Title Abbreviation: J Int AIDS Soc
Journal ID: J Int AIDS Soc
NLM Journal ID: 101478566
Journal ID: JIAS

Journal of the International AIDS Society

EISSN: 1758-2652
Publisher: Wiley

ARTICLE INFORMATION
==============================
Article ID: 18440-038
Subjects: C10 - Epidemiology of HIV in men having sex with men (MSM)

THAC0303: The recent impact of MSM on the prevalence of HIV-1 infection among young men in Thailand

Rangsin R. 1 §
Kana K. 2
Chuenchitra T. 2
Sunantarod A. 3
Meesiri S. 2
Areekul W. 1
Nelson K.E. 4
1 Phramongkutklao College of Medicine, Military and Community Medicine, Bangkok, Thailand
2 Armed Forces Research Institute of Medical Sciences, Bangkok, Thailand
3 Royal Thai Army Institute of Pathology, Bangkok, Thailand
4 Johns Hopkins Bloomberg School Public Health, Epidemiology, Baltimore, United States
§ Presenting author email: r_rangsin@yahoo.com
Electronic publication date: 2012 Oct 22
Publication date: 2012
Volume: 15
Issue: Suppl 3
Electronic Location ID: 18440-038
Copyright: © 2012 R. Rangsin et al.
Copyright year: 2012
License: This is an open-access article distributed under the terms of the Creative Commons Attribution License, which permits unrestricted use, distribution, and reproduction in any medium, provided the original work is properly cited.
License URL: http://creativecommons.org/licenses/by/2.0/

JOURNAL INFORMATION
==============================
NLM Title Abbreviation: J Int AIDS Soc
Journal ID: J Int AIDS Soc
NLM Journal ID: 101478566
Journal ID: JIAS

Journal of the International AIDS Society

EISSN: 1758-2652
Publisher: Wiley

ARTICLE INFORMATION
==============================
Article ID: 18440-039
Subjects: C10 - Epidemiology of HIV in men having sex with men (MSM)

THAC0304: Need for innovative intervention strategies to reduce HIV transmission among men who have sex with men in Andhra Pradesh, India: following a large-scale HIV prevention intervention

Khobragade S. 1
Goswami P. 2 §
Ramakrishnan L. 2
George B. 2
Adhikary R. 3
Ramanathan S. 2
Mathew S. 2
Hari Kumar R. 4
Kodali V. 4
1 FHI 360 India, Mumbai, India
2 FHI 360 India, Delhi, India
3 FHI 360 Washington DC, Washington, United States
4 National Institute of Nutrition (NIN), Hyderabad, India
§ Presenting author email: pgoswami@fhiindia.org
Electronic publication date: 2012 Oct 22
Publication date: 2012
Volume: 15
Issue: Suppl 3
Electronic Location ID: 18440-039
Copyright: © 2012 P. Goswami et al.
Copyright year: 2012
License: This is an open-access article distributed under the terms of the Creative Commons Attribution License, which permits unrestricted use, distribution, and reproduction in any medium, provided the original work is properly cited.
License URL: http://creativecommons.org/licenses/by/2.0/

JOURNAL INFORMATION
==============================
NLM Title Abbreviation: J Int AIDS Soc
Journal ID: J Int AIDS Soc
NLM Journal ID: 101478566
Journal ID: JIAS

Journal of the International AIDS Society

EISSN: 1758-2652
Publisher: Wiley

ARTICLE INFORMATION
==============================
Article ID: 18440-040
Subjects: C10 - Epidemiology of HIV in men having sex with men (MSM)

FRLBX03: HIV prevalence, sexual risks and HIV knowledge among men who have sex with men (MSM) in Malawi: understanding risks among a stigmatized population and opportunities for interventions

Wirtz A. 1 §
Trapence G. 2
Jumbe V. 3
Umar E. 3
Ketende S. 1
Kamba D. 2
Berry M. 1 4
Stromdahl S. 1 5
Beyrer C. 1
Baral S. 1
1 Johns Hopkins Bloomberg School of Public Health, Epidemiology, Baltimore, United States
2 Center for the Development of People, Blantyre, Malawi
3 Malawi College of Medicine, Blantyre, Malawi
4 Johns Hopkins Bloomberg School of Public Health, International Health, Baltimore, United States
5 Karolinska Institutet, Public Health Sciences, Stockholm, Sweden
§ Presenting author email: awirtz@jhsph.edu
Electronic publication date: 2012 Oct 22
Publication date: 2012
Volume: 15
Issue: Suppl 3
Electronic Location ID: 18440-040
Copyright: © 2012 A. Wirtz et al.
Copyright year: 2012
License: This is an open-access article distributed under the terms of the Creative Commons Attribution License, which permits unrestricted use, distribution, and reproduction in any medium, provided the original work is properly cited.
License URL: http://creativecommons.org/licenses/by/2.0/

JOURNAL INFORMATION
==============================
NLM Title Abbreviation: J Int AIDS Soc
Journal ID: J Int AIDS Soc
NLM Journal ID: 101478566
Journal ID: JIAS

Journal of the International AIDS Society

EISSN: 1758-2652
Publisher: Wiley

ARTICLE INFORMATION
==============================
Article ID: 18440-041
Subjects: C10 - Epidemiology of HIV in men having sex with men (MSM)

TUPDC0301: Willingness to take daily pre-exposure prophylaxis (PrEP) among MSM in two HIV epicenters in the United States

Metsch L. 1 §
Kuo I. 2
Lalota M. 3
Phillips Ii G. 2
Cardenas G. 1
Castel A. 2
Forrest D. 1
West T. 2
Pereyra M. 1
Jia Y. 2
Kolber M. 4
Opoku J. 2
Magnus M. 2
1 University of Miami Miller School of Medicine, Department of Epidemiology and Public Health, Miami, United States
2 George Washington University Medical Center, Division of Infectious Diseases, Washington, United States
3 Florida Department of Health, Tallahassee, United States
4 University of Miami Miller School of Medicine, Medicine, Miami, United States
§ Presenting author email: lmetsch@med.miami.edu
Electronic publication date: 2012 Oct 22
Publication date: 2012
Volume: 15
Issue: Suppl 3
Electronic Location ID: 18440-041
Copyright: © 2012 L. Metsch et al.
Copyright year: 2012
License: This is an open-access article distributed under the terms of the Creative Commons Attribution License, which permits unrestricted use, distribution, and reproduction in any medium, provided the original work is properly cited.
License URL: http://creativecommons.org/licenses/by/2.0/

JOURNAL INFORMATION
==============================
NLM Title Abbreviation: J Int AIDS Soc
Journal ID: J Int AIDS Soc
NLM Journal ID: 101478566
Journal ID: JIAS

Journal of the International AIDS Society

EISSN: 1758-2652
Publisher: Wiley

ARTICLE INFORMATION
==============================
Article ID: 18440-042
Subjects: C10 - Epidemiology of HIV in men having sex with men (MSM)

THPDC0104: The MSM population in a conservative environment: Egypt

Elkamhawi S. 1 §
Abaza O. 1
Elghamrawy E. 1
Abou Elmagd S. 2
Ramy H. 2
Atallah S. 3
Elkot N. 4
Kamal M. 5
Mawazini F. 6
Helmy R. 1
Soliman C. 1
1 FHI 360, Cairo, Egypt
2 Waay NGO, Cairo, Egypt
3 Befrienders NGO, Cairo, Egypt
4 Hayat NGO, Cairo, Egypt
5 Ford Foundation, Cairo, Egypt
6 Drosos Foundation, Cairo, Egypt
§ Presenting author email: selkamhawi@fhi-egypt.org
Electronic publication date: 2012 Oct 22
Publication date: 2012
Volume: 15
Issue: Suppl 3
Electronic Location ID: 18440-042
Copyright: © 2012 S. Elkamhawi et al.
Copyright year: 2012
License: This is an open-access article distributed under the terms of the Creative Commons Attribution License, which permits unrestricted use, distribution, and reproduction in any medium, provided the original work is properly cited.
License URL: http://creativecommons.org/licenses/by/2.0/

JOURNAL INFORMATION
==============================
NLM Title Abbreviation: J Int AIDS Soc
Journal ID: J Int AIDS Soc
NLM Journal ID: 101478566
Journal ID: JIAS

Journal of the International AIDS Society

EISSN: 1758-2652
Publisher: Wiley

ARTICLE INFORMATION
==============================
Article ID: 18440-043
Subjects: C11 - Epidemiology of HIV in migrants

MOPDC0203: From assessment to action: HIV in migrant populations in Europe

Pharris A. 1 §
Noori T. 1
Likatavicius G. 1
van de Laar M. 1
1 European Centre for Disease Prevention and Control (ECDC), Programme for HIV/AIDS, STI and Blood-Borne Infections, Stockholm, Sweden
§ Presenting author email: anastasia.pharris@ecdc.europa.eu
Electronic publication date: 2012 Oct 22
Publication date: 2012
Volume: 15
Issue: Suppl 3
Electronic Location ID: 18440-043
Copyright: © 2012 A. Pharris et al.
Copyright year: 2012
License: This is an open-access article distributed under the terms of the Creative Commons Attribution License, which permits unrestricted use, distribution, and reproduction in any medium, provided the original work is properly cited.
License URL: http://creativecommons.org/licenses/by/2.0/

JOURNAL INFORMATION
==============================
NLM Title Abbreviation: J Int AIDS Soc
Journal ID: J Int AIDS Soc
NLM Journal ID: 101478566
Journal ID: JIAS

Journal of the International AIDS Society

EISSN: 1758-2652
Publisher: Wiley

ARTICLE INFORMATION
==============================
Article ID: 18440-044
Subjects: C12 - Epidemiology of HIV in other populations including transgender

THPDC0202: Global burden of HIV infection among transgender persons: a systematic review and meta-analysis

Baral S. 1 §
Poteat T. 1
Wirtz A. 1
Stromdahl S. 1
Beyrer C. 1
1 Center for Public Health and Human Rights, JHSPH, Epidemiology, Baltimore, United States
§ Presenting author email: sbaral@jhsph.edu
Electronic publication date: 2012 Oct 22
Publication date: 2012
Volume: 15
Issue: Suppl 3
Electronic Location ID: 18440-044
Copyright: © 2012 S. Baral et al.
Copyright year: 2012
License: This is an open-access article distributed under the terms of the Creative Commons Attribution License, which permits unrestricted use, distribution, and reproduction in any medium, provided the original work is properly cited.
License URL: http://creativecommons.org/licenses/by/2.0/

JOURNAL INFORMATION
==============================
NLM Title Abbreviation: J Int AIDS Soc
Journal ID: J Int AIDS Soc
NLM Journal ID: 101478566
Journal ID: JIAS

Journal of the International AIDS Society

EISSN: 1758-2652
Publisher: Wiley

ARTICLE INFORMATION
==============================
Article ID: 18440-045
Subjects: C14 - Epidemiology of sexually transmitted infections (STI) and HIV

TUAC0201: Evaluation of presumptive treatment recommendation for asymptomatic anorectal gonorrhoea and chlamydia infections in at-risk MSM in Kenya

Okuku H.S. 1
Wahome E. 1
Duncan S. 1
Thiongo’ A. 1
Mwambi J. 1
Shafi J. 2
Smith A.D. 3
Graham S.M. 1 4
Sanders E.J. 1 5 §
1 Centre for Geographic Medicine Research-Coast, Kenya Medical Research Institute (KEMRI), Kilifi, Kenya
2 University of Nairobi, Mombasa, Kenya
3 Department of Public Health, University of Oxford, Headington, Oxford, United Kingdom
4 University of Washington, Seattle, United States
5 Nuffield Department of Medicine, University of Oxford, Headington, Oxford, United Kingdom
§ Presenting author email: esanders@kilifi.kemri-wellcome.org
Electronic publication date: 2012 Oct 22
Publication date: 2012
Volume: 15
Issue: Suppl 3
Electronic Location ID: 18440-045
Copyright: © 2012 E.J. Sanders et al.
Copyright year: 2012
License: This is an open-access article distributed under the terms of the Creative Commons Attribution License, which permits unrestricted use, distribution, and reproduction in any medium, provided the original work is properly cited.
License URL: http://creativecommons.org/licenses/by/2.0/

JOURNAL INFORMATION
==============================
NLM Title Abbreviation: J Int AIDS Soc
Journal ID: J Int AIDS Soc
NLM Journal ID: 101478566
Journal ID: JIAS

Journal of the International AIDS Society

EISSN: 1758-2652
Publisher: Wiley

ARTICLE INFORMATION
==============================
Article ID: 18440-046
Subjects: C14 - Epidemiology of sexually transmitted infections (STI) and HIV

TUPDC0103: Gonorrhea infections diagnosed among persons living with HIV: cross matching surveillance registries to identify potential opportunities for integrated partner services-New York City, Washington D.C., Miami/Dade County and Arizona

Taylor M. 1 2
Schillinger J. 1 3
Pathela P. 3
Skinner J. 2
Newman D. 1
Braunstein S. 3
Shepard C. 3
Brewer T. 1 4
Ahmed T. 5
Griffin A. 5
Furness B. 5 §
1 Centers for Disease Control and Prevention, Division of STD Prevention, Atlanta, United States
2 Arizona Department of Health Services, STD Program, Phoenix, United States
3 New York City, Department of Health and Mental Hygiene, New York City, United States
4 Miami/Dade County Department of Health, Miami, United States
5 HIV/AIDS, Hepatitis, STD and TB Administration, District of Columbia, Department of Health, Washington, United States
§
Electronic publication date: 2012 Oct 22
Publication date: 2012
Volume: 15
Issue: Suppl 3
Electronic Location ID: 18440-046
Copyright: © 2012 M. Taylor et al.
Copyright year: 2012
License: This is an open-access article distributed under the terms of the Creative Commons Attribution License, which permits unrestricted use, distribution, and reproduction in any medium, provided the original work is properly cited.
License URL: http://creativecommons.org/licenses/by/2.0/

JOURNAL INFORMATION
==============================
NLM Title Abbreviation: J Int AIDS Soc
Journal ID: J Int AIDS Soc
NLM Journal ID: 101478566
Journal ID: JIAS

Journal of the International AIDS Society

EISSN: 1758-2652
Publisher: Wiley

ARTICLE INFORMATION
==============================
Article ID: 18440-047
Subjects: C14 - Epidemiology of sexually transmitted infections (STI) and HIV

TUPDC0105: Sexually transmitted infection incidence as a biomarker for high risk sexual behaviour in individuals diagnosed with acute HIV after entering care

Cope A. 1
Crooks A. 2
Kuruc J. 2
Sugarbaker A. 2
Hightow-Weidman L. 2
Eron J. 2
Gay C. 2 §
1 University of North Carolina, Epidemiology, Chapel Hill, United States
2 University of North Carolina, Medicine, Chapel Hill, United States
§ Presenting author email: acbarry@unc.edu
Electronic publication date: 2012 Oct 22
Publication date: 2012
Volume: 15
Issue: Suppl 3
Electronic Location ID: 18440-047
Copyright: © 2012 A. Cope et al.
Copyright year: 2012
License: This is an open-access article distributed under the terms of the Creative Commons Attribution License, which permits unrestricted use, distribution, and reproduction in any medium, provided the original work is properly cited.
License URL: http://creativecommons.org/licenses/by/2.0/

JOURNAL INFORMATION
==============================
NLM Title Abbreviation: J Int AIDS Soc
Journal ID: J Int AIDS Soc
NLM Journal ID: 101478566
Journal ID: JIAS

Journal of the International AIDS Society

EISSN: 1758-2652
Publisher: Wiley

ARTICLE INFORMATION
==============================
Article ID: 18440-048
Subjects: C15 - Epidemiology of viral hepatitis and HIV co-infection

TUAC0501: Use of rapid testing to prospectively identify HCV co-infection in populations with high HIV prevalence

Lee S. 1
Roehler M. 1
Guillon G. 1
Schiff E. 2
Delgado-Borrego A. 2
Friel T. 3
La Marca A. 4
Dickson R. 5
Sulkowski M. 6
Borders J. 7
Allen G. 8
Kurtz L. 1
Kardos K. 1
Gregg R. 1
1 OraSure Technologies, Inc., Bethlehem, United States
2 University of Miami, Miller School of Medicine, Miami, United States
3 Lehigh Valley Hospital, Department of Medicine Research, Allentown, United States
4 Therafirst Medical Center, Ft. Lauderdale, United States
5 Dartmouth Hitchcock Medical Center, Lebanon, United States
6 Johns Hopkins Medical Institutes, Baltimore, United States
7 Central Kentucky Research Associates, Inc., Lexington, United States
8 New England Center for Clinical Research of Massachusetts, LLC, New Bedford, United States
§
Electronic publication date: 2012 Oct 22
Publication date: 2012
Volume: 15
Issue: Suppl 3
Electronic Location ID: 18440-048
Copyright: © 2012 S. Lee et al.
Copyright year: 2012
License: This is an open-access article distributed under the terms of the Creative Commons Attribution License, which permits unrestricted use, distribution, and reproduction in any medium, provided the original work is properly cited.
License URL: http://creativecommons.org/licenses/by/2.0/

JOURNAL INFORMATION
==============================
NLM Title Abbreviation: J Int AIDS Soc
Journal ID: J Int AIDS Soc
NLM Journal ID: 101478566
Journal ID: JIAS

Journal of the International AIDS Society

EISSN: 1758-2652
Publisher: Wiley

ARTICLE INFORMATION
==============================
Article ID: 18440-049
Subjects: C15 - Epidemiology of viral hepatitis and HIV co-infection

TUAC0502: Seroprevalence of HBV and HCV among children in the Kilimanjaro region of Tanzania

Muro F.J. 1
Fiorillo S.P. 2
Odhiambo C. 1
Cunningham C.K. 3
Buchanan A. 1 3 4 §
1 Kilimanjaro Christian Medical Centre, Moshi, United Republic of Tanzania
2 University of Colorado, Denver, Division of Infectious Diseases, Aurora, United States
3 Duke University Medical Center, Division of Infectious Diseases, Department of Pediatrics, Durham, United States
4 Duke Global Health Institute, Duke University, Durham, United Republic of Tanzania
§ Presenting author email: ann.buchanan@duke.edu
Electronic publication date: 2012 Oct 22
Publication date: 2012
Volume: 15
Issue: Suppl 3
Electronic Location ID: 18440-049
Copyright: © 2012 A. Buchanan et al.
Copyright year: 2012
License: This is an open-access article distributed under the terms of the Creative Commons Attribution License, which permits unrestricted use, distribution, and reproduction in any medium, provided the original work is properly cited.
License URL: http://creativecommons.org/licenses/by/2.0/

JOURNAL INFORMATION
==============================
NLM Title Abbreviation: J Int AIDS Soc
Journal ID: J Int AIDS Soc
NLM Journal ID: 101478566
Journal ID: JIAS

Journal of the International AIDS Society

EISSN: 1758-2652
Publisher: Wiley

ARTICLE INFORMATION
==============================
Article ID: 18440-050
Subjects: C15 - Epidemiology of viral hepatitis and HIV co-infection

TUAC0503: Prevalence, incidence and determinants of HCV infections among HIV-positive MSM attending a STI clinic, 1995–2010

Urbanus A.T. 1 §
van de Laar T. 2
Geskus R. 3
Vanhommerig J. 4
van Rooijen M. 4
Schinkel J. 5
Heijman T. 4
Coutinho R. 6 7
Prins M. 4 8
1 Public Health Service, Cluster of Infectious Diseases, Amsterdam, Netherlands
2 VUmc, Amsterdam, Netherlands
3 Academic Medical Center (AMC), Department of Clinical Epidemiology, Biostatistics and Bioinformatics, Amsterdam, Netherlands
4 Public Health Service Amsterdam, Amsterdam, Netherlands
5 Academic Medical Center (AMC), Department of Clinical Virology, Amsterdam, Netherlands
6 National Institute for Public Health and the Environment (RIVM), Center for Infectious Disease Control, Bilthoven, Netherlands
7 Julius Center for Health Science and Primary Care, Utrecht, Netherlands
8 Academic Medical Center (AMC), Division of Infectious Diseases, Tropical Medicine and AIDS (CINIMA), Amstedam, Netherlands
§ Presenting author email: aurbanus@ggd.amsterdam.nl
Electronic publication date: 2012 Oct 22
Publication date: 2012
Volume: 15
Issue: Suppl 3
Electronic Location ID: 18440-050
Copyright: © 2012 A.T. Urbanus et al.
Copyright year: 2012
License: This is an open-access article distributed under the terms of the Creative Commons Attribution License, which permits unrestricted use, distribution, and reproduction in any medium, provided the original work is properly cited.
License URL: http://creativecommons.org/licenses/by/2.0/

JOURNAL INFORMATION
==============================
NLM Title Abbreviation: J Int AIDS Soc
Journal ID: J Int AIDS Soc
NLM Journal ID: 101478566
Journal ID: JIAS

Journal of the International AIDS Society

EISSN: 1758-2652
Publisher: Wiley

ARTICLE INFORMATION
==============================
Article ID: 18440-051
Subjects: C15 - Epidemiology of viral hepatitis and HIV co-infection

TUAC0504: HCV genotype and HBV co-infection associate with HCV clearance in HIV-positive subjects

Dong Y. 1 §
Qiu C. 1
Xia X. 2
Wang J. 1
Zhang H. 3
Wang Y. 3
Zhang X. 1 4
Xu J. 1 4
1 Fudan University, Shanghai Public Health Clinical Center and Institutes of Biomedical Sciences, Shanghai, China
2 Faculty of Life Science and Technology, Kunming University of Science and Technology, Kunming, China
3 Dali Municipal Centers for Disease Control and Prevention, Dali, China
4 China CDC, State Key Laboratory of Infectious Disease Prevention and Control, Beijing, China
§ Presenting author email: dongyuan5177@gmail.com
Electronic publication date: 2012 Oct 22
Publication date: 2012
Volume: 15
Issue: Suppl 3
Electronic Location ID: 18440-051
Copyright: © 2012 Y. Dong et al.
Copyright year: 2012
License: This is an open-access article distributed under the terms of the Creative Commons Attribution License, which permits unrestricted use, distribution, and reproduction in any medium, provided the original work is properly cited.
License URL: http://creativecommons.org/licenses/by/2.0/

JOURNAL INFORMATION
==============================
NLM Title Abbreviation: J Int AIDS Soc
Journal ID: J Int AIDS Soc
NLM Journal ID: 101478566
Journal ID: JIAS

Journal of the International AIDS Society

EISSN: 1758-2652
Publisher: Wiley

ARTICLE INFORMATION
==============================
Article ID: 18440-052
Subjects: C16 - Epidemiology of other diseases and HIV

MOAC0202: Impact of syndemics on people living with HIV in San Francisco

Chu P.L. 1 §
Santos G.-M. 2
Vu A. 3
Nieves-Rivera I. 1
Colfax G.N. 2
Grinsdale J. 4
Huang S. 5
Philip S. 6
Scheer S. 3
Aragon T. 1
1 San Francisco Department of Public Health, Population Health and Prevention, San Francisco, United States
2 San Francisco Department of Public Health, HIV Prevention Section, San Francisco, United States
3 San Francisco Department of Public Health, HIV Epidemiology Section, San Francisco, United States
4 San Francisco Department of Public Health, Tuberculosis Control, San Francisco, United States
5 San Francisco Department of Public Health, Communicable Disease Control and Prevention, San Francisco, United States
6 San Francisco Department of Public Health, STD Prevention and Control Section, San Francisco, United States
§ Presenting author email: priscilla.chu@sfdph.org
Electronic publication date: 2012 Oct 22
Publication date: 2012
Volume: 15
Issue: Suppl 3
Electronic Location ID: 18440-052
Copyright: © 2012 P.L. Chu et al.
Copyright year: 2012
License: This is an open-access article distributed under the terms of the Creative Commons Attribution License, which permits unrestricted use, distribution, and reproduction in any medium, provided the original work is properly cited.
License URL: http://creativecommons.org/licenses/by/2.0/

JOURNAL INFORMATION
==============================
NLM Title Abbreviation: J Int AIDS Soc
Journal ID: J Int AIDS Soc
NLM Journal ID: 101478566
Journal ID: JIAS

Journal of the International AIDS Society

EISSN: 1758-2652
Publisher: Wiley

ARTICLE INFORMATION
==============================
Article ID: 18440-053
Subjects: C17 - Molecular epidemiology

MOAC0204: Using phylogenetic analysis to identify HIV transmission channels among persons newly diagnosed with HIV-1 infection in Los Angeles County, 2009–2010

Sey K. 1 §
Ma Y. 1
Song N. 1
1 HIV Epidemiology Program, Los Angeles County Department of Public Health, Los Angeles, United States
§ Presenting author email: esey@ph.lacounty.gov
Electronic publication date: 2012 Oct 22
Publication date: 2012
Volume: 15
Issue: Suppl 3
Electronic Location ID: 18440-053
Copyright: © 2012 K. Sey et al.
Copyright year: 2012
License: This is an open-access article distributed under the terms of the Creative Commons Attribution License, which permits unrestricted use, distribution, and reproduction in any medium, provided the original work is properly cited.
License URL: http://creativecommons.org/licenses/by/2.0/

JOURNAL INFORMATION
==============================
NLM Title Abbreviation: J Int AIDS Soc
Journal ID: J Int AIDS Soc
NLM Journal ID: 101478566
Journal ID: JIAS

Journal of the International AIDS Society

EISSN: 1758-2652
Publisher: Wiley

ARTICLE INFORMATION
==============================
Article ID: 18440-054
Subjects: C17 - Molecular epidemiology

MOPDC0204: High prevalence of HIV-1 intersubtypes multiple infections in the metropolitan region of Sao Paulo, Brazil

Nunes E.R.M. 1
Maricato J.T. 2
Zukurov J.P. 1
Janini L.M.R. 2
1 Federal University Sao Paulo, Medicine, Sao Paulo, Brazil
2 Federal University Sao Paulo, Microbiology, Immunology and Parasitology, Sao Paulo, Brazil
Electronic publication date: 2012 Oct 22
Publication date: 2012
Volume: 15
Issue: Suppl 3
Electronic Location ID: 18440-054
Copyright: © 2012 et al.
Copyright year: 2012
License: This is an open-access article distributed under the terms of the Creative Commons Attribution License, which permits unrestricted use, distribution, and reproduction in any medium, provided the original work is properly cited.
License URL: http://creativecommons.org/licenses/by/2.0/

JOURNAL INFORMATION
==============================
NLM Title Abbreviation: J Int AIDS Soc
Journal ID: J Int AIDS Soc
NLM Journal ID: 101478566
Journal ID: JIAS

Journal of the International AIDS Society

EISSN: 1758-2652
Publisher: Wiley

ARTICLE INFORMATION
==============================
Article ID: 18440-055
Subjects: C22 - Capacity building for HIV prevention research

THAC0505: Capacity building of law enforcement officers to handle sex workers In Sri Lanka

Vidanapathirana H.M.J.P. 1 §
Sangeeth M. 2
1 National HIV/AIDS Prevention Project, Ministry of Health, Nugegoda, Sri Lanka
2 Posittive Women Net Work, Community Based Organization, Colombo, Sri Lanka
§ Presenting author email: kavigaya@yahoo.com
Electronic publication date: 2012 Oct 22
Publication date: 2012
Volume: 15
Issue: Suppl 3
Electronic Location ID: 18440-055
Copyright: © 2012 H.M.J.P. Vidanapathirana et al.
Copyright year: 2012
License: This is an open-access article distributed under the terms of the Creative Commons Attribution License, which permits unrestricted use, distribution, and reproduction in any medium, provided the original work is properly cited.
License URL: http://creativecommons.org/licenses/by/2.0/

JOURNAL INFORMATION
==============================
NLM Title Abbreviation: J Int AIDS Soc
Journal ID: J Int AIDS Soc
NLM Journal ID: 101478566
Journal ID: JIAS

Journal of the International AIDS Society

EISSN: 1758-2652
Publisher: Wiley

ARTICLE INFORMATION
==============================
Article ID: 18440-056
Subjects: C23 - Measuring and modeling the HIV epidemic

MOPDC0305: Patient migration significantly impacts estimates of engagement in HIV care and attainment of an undetectable HIV-RNA level in a cohort of newly HIV-diagnosed individuals

Rowan S. 1 2 §
Johnson S. 1
Thrun M. 1 2
Daniloff E. 3
Reirden D. 4
Burman W. 1 2
Connick E. 1
Gardner E. 1 2
1 University of Colorado Denver, Division of Infectious Diseases, Denver, United States
2 Denver Public Health, Denver, United States
3 Colorado Department of Public Health and Environment, Denver, United States
4 The Children's Hospital, Aurora, United States
§ Presenting author email: sarah.rowan@ucdenver.edu
Electronic publication date: 2012 Oct 22
Publication date: 2012
Volume: 15
Issue: Suppl 3
Electronic Location ID: 18440-056
Copyright: © 2012 S. Rowan et al.
Copyright year: 2012
License: This is an open-access article distributed under the terms of the Creative Commons Attribution License, which permits unrestricted use, distribution, and reproduction in any medium, provided the original work is properly cited.
License URL: http://creativecommons.org/licenses/by/2.0/

JOURNAL INFORMATION
==============================
NLM Title Abbreviation: J Int AIDS Soc
Journal ID: J Int AIDS Soc
NLM Journal ID: 101478566
Journal ID: JIAS

Journal of the International AIDS Society

EISSN: 1758-2652
Publisher: Wiley

ARTICLE INFORMATION
==============================
Article ID: 18440-057
Subjects: C23 - Measuring and modeling the HIV epidemic

THPDC0101: Sexual mixing patterns between men who have sex with men in southern India: implications for modelling the HIV epidemic and predicting the impact of targeted oral pre-exposure prophylaxis

Mitchell K.M. 1 §
Foss A.M. 1
Prudden H.J. 1
Pickles M. 1 2
Williams J.R. 2
Johnson H.C. 1
Ramesh B.M. 3 4
Washington R. 3 5
Isac S. 3
Rajaram S. 6
Phillips A.E. 2
Bradley J. 6 7
Alary M. 6 7
Moses S. 4
Lowndes C.M. 7 8
Watts C.H. 1
Boily M.-C. 2
Vickerman P. 1 9
1 London School of Hygiene and Tropical Medicine, London, United Kingdom
2 Imperial College London, London, United Kingdom
3 Karnataka Health Promotion Trust, Bangalore, India
4 University of Manitoba, Winnipeg, Canada
5 St. John's Research Institute, Bangalore, India
6 CHARME-India Project, Bangalore, India
7 Centre Hospitalier Affilié Universitaire de Québec, Québec, Canada
8 Health Protection Agency, London, United Kingdom
9 University of Bristol, Bristol, United Kingdom
§ Presenting author email: kate.mitchell@lshtm.ac.uk
Electronic publication date: 2012 Oct 22
Publication date: 2012
Volume: 15
Issue: Suppl 3
Electronic Location ID: 18440-057
Copyright: © 2012 K.M. Mitchell et al.
Copyright year: 2012
License: This is an open-access article distributed under the terms of the Creative Commons Attribution License, which permits unrestricted use, distribution, and reproduction in any medium, provided the original work is properly cited.
License URL: http://creativecommons.org/licenses/by/2.0/

JOURNAL INFORMATION
==============================
NLM Title Abbreviation: J Int AIDS Soc
Journal ID: J Int AIDS Soc
NLM Journal ID: 101478566
Journal ID: JIAS

Journal of the International AIDS Society

EISSN: 1758-2652
Publisher: Wiley

ARTICLE INFORMATION
==============================
Article ID: 18440-058
Subjects: C24 - Measuring and modeling the effect and impact of HIV prevention interventions

THAC0502: Modeling the impacts of a comprehensive community empowerment-based, HIV prevention intervention for female sex workers in generalized and concentrated epidemics: infections averted among sex workers and adults

Wirtz A. 1 §
Pretorius C. 2
Sherman S. 1
Baral S. 1
Decker M. 3
Sweat M. 4
Beyrer C. 1
Kerrigan D. 5
1 Johns Hopkins Bloomberg School of Public Health, Epidemiology, Baltimore, United States
2 Futures Institute, New Haven, United States
3 Johns Hopkins Bloomberg School of Public HealthDepartment of Population, Family and Reproductive Health, Baltimore, United States
4 Medical University of South Carolina, Charleston, United States
5 Johns Hopkins Bloomberg School of Public Health, International Health, Baltimore, United States
§ Presenting author email: awirtz@jhsph.edu
Electronic publication date: 2012 Oct 22
Publication date: 2012
Volume: 15
Issue: Suppl 3
Electronic Location ID: 18440-058
Copyright: © 2012 A. Wirtz et al.
Copyright year: 2012
License: This is an open-access article distributed under the terms of the Creative Commons Attribution License, which permits unrestricted use, distribution, and reproduction in any medium, provided the original work is properly cited.
License URL: http://creativecommons.org/licenses/by/2.0/

JOURNAL INFORMATION
==============================
NLM Title Abbreviation: J Int AIDS Soc
Journal ID: J Int AIDS Soc
NLM Journal ID: 101478566
Journal ID: JIAS

Journal of the International AIDS Society

EISSN: 1758-2652
Publisher: Wiley

ARTICLE INFORMATION
==============================
Article ID: 18440-059
Subjects: C24 - Measuring and modeling the effect and impact of HIV prevention interventions

THAC0504: Impact of behaviour change communication targeting the bridging population of clients of female sex workers in India

Suryawanshi D. 1
Swain P. 2
Kumari R. 3
Bhatnagar T. 4 §
Zhou W. 5
Bharat S. 1
Bhattacharya M. 6
Pandey A. 3
Collumbien M. 5
1 Tata Institute of Social Sciences (TISS), School of Health System Studies (SHSS), Mumbai, India
2 National Institute of Health and Family Welfare, Department of Statistics and Demography, New Delhi, India
3 National Institute of Medical Statistics, New Delhi, India
4 National Institute of Epidemiology-ICMR, Chennai, India
5 London School of Hygiene and Tropical Medicine, Department of Social and Environmental Health Research, London, United Kingdom
6 National Institute of Health and Family Welfare, New Delhi, India
§ Presenting author email: drtarunb@gmail.com
Electronic publication date: 2012 Oct 22
Publication date: 2012
Volume: 15
Issue: Suppl 3
Electronic Location ID: 18440-059
Copyright: © 2012 T. Bhatnagar et al.
Copyright year: 2012
License: This is an open-access article distributed under the terms of the Creative Commons Attribution License, which permits unrestricted use, distribution, and reproduction in any medium, provided the original work is properly cited.
License URL: http://creativecommons.org/licenses/by/2.0/

JOURNAL INFORMATION
==============================
NLM Title Abbreviation: J Int AIDS Soc
Journal ID: J Int AIDS Soc
NLM Journal ID: 101478566
Journal ID: JIAS

Journal of the International AIDS Society

EISSN: 1758-2652
Publisher: Wiley

ARTICLE INFORMATION
==============================
Article ID: 18440-060
Subjects: C24 - Measuring and modeling the effect and impact of HIV prevention interventions

FRLBC05: Combination interventions for the prevention of HIV among injection drug users: a complex systems dynamics model

Marshall B. 1 §
Paczkowski M. 2
Tempalski B. 3
Pouget E. 3
Friedman S. 3
Galea S. 2
1 Brown University, Department of Epidemiology, Providence, United States
2 Columbia University, New York, Department of Epidemiology, United States
3 National Development and Research Institues. Inc., Institute for AIDS Research, New York, United States
§ Presenting author email: brandon_marshall@brown.edu
Electronic publication date: 2012 Oct 22
Publication date: 2012
Volume: 15
Issue: Suppl 3
Electronic Location ID: 18440-060
Copyright: © 2012 B. Marshall et al.
Copyright year: 2012
License: This is an open-access article distributed under the terms of the Creative Commons Attribution License, which permits unrestricted use, distribution, and reproduction in any medium, provided the original work is properly cited.
License URL: http://creativecommons.org/licenses/by/2.0/

JOURNAL INFORMATION
==============================
NLM Title Abbreviation: J Int AIDS Soc
Journal ID: J Int AIDS Soc
NLM Journal ID: 101478566
Journal ID: JIAS

Journal of the International AIDS Society

EISSN: 1758-2652
Publisher: Wiley

ARTICLE INFORMATION
==============================
Article ID: 18440-061
Subjects: C24 - Measuring and modeling the effect and impact of HIV prevention interventions

MOPDC0102: Modeling the impact of focused strategies on the cost and effectiveness of TLC-Plus (or ‘Test and Treat’) in New York City

Kessler J. 1 §
Myers J. 2
Nucifora K. 1
Mensah N. 2
Kowalski A. 1
Sweeney M. 2
Toohey C. 1
Shepard C. 2
Cutler B. 2
Braithwaite S. 1
1 NYU School of Medicine, Division of Population Health, New York, United States
2 Department of Health and Mental Hygiene, Bureau of HIV/AIDS Prevention and Control, New York, United States
§ Presenting author email: jason.kessler@nyumc.org
Electronic publication date: 2012 Oct 22
Publication date: 2012
Volume: 15
Issue: Suppl 3
Electronic Location ID: 18440-061
Copyright: © 2012 J. Kessler et al.
Copyright year: 2012
License: This is an open-access article distributed under the terms of the Creative Commons Attribution License, which permits unrestricted use, distribution, and reproduction in any medium, provided the original work is properly cited.
License URL: http://creativecommons.org/licenses/by/2.0/

JOURNAL INFORMATION
==============================
NLM Title Abbreviation: J Int AIDS Soc
Journal ID: J Int AIDS Soc
NLM Journal ID: 101478566
Journal ID: JIAS

Journal of the International AIDS Society

EISSN: 1758-2652
Publisher: Wiley

ARTICLE INFORMATION
==============================
Article ID: 18440-062
Subjects: C24 - Measuring and modeling the effect and impact of HIV prevention interventions

TUPDC0302: Perceptions and attitudes about PrEP among seronegative partners and the potential of sexual disinhibition associated with the use of PrEP

Tripathi A. 1
Whiteside O. 2
Scanlon C. 2
Duffus W. 2 §
1 Epidemiology and Biostatistics, University of South Carolina, Columbia, United States
2 South Carolina Department of Health and Environmental Control, Bureau of Disease Control, Columbia, United States
§ Presenting author email: duffuswa@dhec.sc.gov
Electronic publication date: 2012 Oct 22
Publication date: 2012
Volume: 15
Issue: Suppl 3
Electronic Location ID: 18440-062
Copyright: © 2012 W. Duffus et al.
Copyright year: 2012
License: This is an open-access article distributed under the terms of the Creative Commons Attribution License, which permits unrestricted use, distribution, and reproduction in any medium, provided the original work is properly cited.
License URL: http://creativecommons.org/licenses/by/2.0/

JOURNAL INFORMATION
==============================
NLM Title Abbreviation: J Int AIDS Soc
Journal ID: J Int AIDS Soc
NLM Journal ID: 101478566
Journal ID: JIAS

Journal of the International AIDS Society

EISSN: 1758-2652
Publisher: Wiley

ARTICLE INFORMATION
==============================
Article ID: 18440-063
Subjects: C24 - Measuring and modeling the effect and impact of HIV prevention interventions

WEPDC0206: A complex systems approach to evaluate HIV prevention in metropolitan areas: preliminary implications for combination intervention strategies

Marshall B. 1 2 §
Paczkowski M. 2
Seemann L. 3
Tempalski B. 4
Pouget E. 4
Galea S. 2
Friedman S. 4
1 Brown University, Department of Epidemiology, Providence, United States
2 Columbia University, Department of Epidemiology, New York, United States
3 University of Houston, Department of Physics, Houston, United States
4 National Development and Research Institutes, Inc., Institute for AIDS Research, New York, United States
§ Presenting author email: brandon_marshall@brown.edu
Electronic publication date: 2012 Oct 22
Publication date: 2012
Volume: 15
Issue: Suppl 3
Electronic Location ID: 18440-063
Copyright: © 2012 B. Marshall et al.
Copyright year: 2012
License: This is an open-access article distributed under the terms of the Creative Commons Attribution License, which permits unrestricted use, distribution, and reproduction in any medium, provided the original work is properly cited.
License URL: http://creativecommons.org/licenses/by/2.0/

JOURNAL INFORMATION
==============================
NLM Title Abbreviation: J Int AIDS Soc
Journal ID: J Int AIDS Soc
NLM Journal ID: 101478566
Journal ID: JIAS

Journal of the International AIDS Society

EISSN: 1758-2652
Publisher: Wiley

ARTICLE INFORMATION
==============================
Article ID: 18440-064
Subjects: C24 - Measuring and modeling the effect and impact of HIV prevention interventions

MOPDC0207: Gentrification and its effects on HIV/AIDS rates in D.C.

Ahmed T. 1 2 §
Griffin A. 3
West T. 1
Pappas G. 1
1 District of Columbia Department of Health, HIV/AIDS, Hepatitis, STD and TB Administration, Washington, United States
2 George Washington School of Public Health and Health Services, Epidemiology and Biostatistics, Washington, United States
3 District of Columbia Department of Health, HAHSTA, Washington, United States
§ Presenting author email: tashrik@gmail.com
Electronic publication date: 2012 Oct 22
Publication date: 2012
Volume: 15
Issue: Suppl 3
Electronic Location ID: 18440-064
Copyright: © 2012 T. Ahmed et al.
Copyright year: 2012
License: This is an open-access article distributed under the terms of the Creative Commons Attribution License, which permits unrestricted use, distribution, and reproduction in any medium, provided the original work is properly cited.
License URL: http://creativecommons.org/licenses/by/2.0/

JOURNAL INFORMATION
==============================
NLM Title Abbreviation: J Int AIDS Soc
Journal ID: J Int AIDS Soc
NLM Journal ID: 101478566
Journal ID: JIAS

Journal of the International AIDS Society

EISSN: 1758-2652
Publisher: Wiley

ARTICLE INFORMATION
==============================
Article ID: 18440-065
Subjects: C25 - New HIV testing and diagnostic strategies

TUAC0304: Dentist's willingness to offer oral HIV rapid testing: results from a nationally representative survey

Metsch L.R. 1 §
Pollack H.A. 2
Abel S. 3
Kunzel C. 4
Greenberg B. 5
Messinger S. 1
Pereyra M. 1
1 University of Miami Miller School of Medicine, Department of Epidemiology and Public Health, Miami, United States
2 University of Chicago, School of Social Service Administration, Chicago, United States
3 Nova Southeastern University, College of Dental Medicine, Ft. Lauderdale, United States
4 Columbia Mailman School of Public Health, Sociomedical Sciences, New York, United States
5 New Jersey Medical School, UMDNJ, New Jersey Dental School, Newark, United States
§ Presenting author email: lmetsch@med.miami.edu
Electronic publication date: 2012 Oct 22
Publication date: 2012
Volume: 15
Issue: Suppl 3
Electronic Location ID: 18440-065
Copyright: © 2012 L.R. Metsch et al.
Copyright year: 2012
License: This is an open-access article distributed under the terms of the Creative Commons Attribution License, which permits unrestricted use, distribution, and reproduction in any medium, provided the original work is properly cited.
License URL: http://creativecommons.org/licenses/by/2.0/

JOURNAL INFORMATION
==============================
NLM Title Abbreviation: J Int AIDS Soc
Journal ID: J Int AIDS Soc
NLM Journal ID: 101478566
Journal ID: JIAS

Journal of the International AIDS Society

EISSN: 1758-2652
Publisher: Wiley

ARTICLE INFORMATION
==============================
Article ID: 18440-066
Subjects: C25 - New HIV testing and diagnostic strategies

THAC0102: More HIV positive infants and mothers identified through HIV testing in immunization clinics

Schouten E.J. 1 §
Sinunu M. 2
Wadonda N. 3
Kajawo E. 1
Eliya M. 3
Chimbwandira F.M. 3
Kellerman S.E. 4
1 Management Sciences for Health (MSH), Lilongwe, Malawi
2 Boston University School of Public Health, Boston, United States
3 Ministry of Health, Lilongwe, Malawi
4 Management Sciences for Health (MSH), Centre for Health Services, Arlington, United States
§ Presenting author email: eschouten@msh.org
Electronic publication date: 2012 Oct 22
Publication date: 2012
Volume: 15
Issue: Suppl 3
Electronic Location ID: 18440-066
Copyright: © 2012 E.J. Schouten et al.
Copyright year: 2012
License: This is an open-access article distributed under the terms of the Creative Commons Attribution License, which permits unrestricted use, distribution, and reproduction in any medium, provided the original work is properly cited.
License URL: http://creativecommons.org/licenses/by/2.0/

JOURNAL INFORMATION
==============================
NLM Title Abbreviation: J Int AIDS Soc
Journal ID: J Int AIDS Soc
NLM Journal ID: 101478566
Journal ID: JIAS

Journal of the International AIDS Society

EISSN: 1758-2652
Publisher: Wiley

ARTICLE INFORMATION
==============================
Article ID: 18440-067
Subjects: C25 - New HIV testing and diagnostic strategies

TUPDC0101: Tracing sexual contacts of HIV-positive individuals in Taizhou, eastern China

Lin H. 1 §
He N. 1
Ding Y. 1
Zhu W. 2
Detels R. 3
1 Department of Epidemiology, School of Public Health, Fudan University and The Key Laboratory of Public Health Safety of Ministry of Education, Shanghai, China
2 Department of Epidemiology, School of Public Health, Fudan University and The Key Laboratory of Public Health Safety of Ministry of Education, Los Angeles, United States
3 Department of Epidemiology, School of Public Health, University of California, Los Angeles, United States
§ Presenting author email: linhaijiang@hotmail.com
Electronic publication date: 2012 Oct 22
Publication date: 2012
Volume: 15
Issue: Suppl 3
Electronic Location ID: 18440-067
Copyright: © 2012 H. Lin et al.
Copyright year: 2012
License: This is an open-access article distributed under the terms of the Creative Commons Attribution License, which permits unrestricted use, distribution, and reproduction in any medium, provided the original work is properly cited.
License URL: http://creativecommons.org/licenses/by/2.0/

JOURNAL INFORMATION
==============================
NLM Title Abbreviation: J Int AIDS Soc
Journal ID: J Int AIDS Soc
NLM Journal ID: 101478566
Journal ID: JIAS

Journal of the International AIDS Society

EISSN: 1758-2652
Publisher: Wiley

ARTICLE INFORMATION
==============================
Article ID: 18440-068
Subjects: C25 - New HIV testing and diagnostic strategies

THPDC0103: Faster and integral HIV diagnosis among MSM in the HIV/AIDS program of Mexico City (HIVPMC): necessary but not sufficient

Juárez-Figueroa L. 1 §
Gonzalez-Rodríguez A. 2
Casillas J. 3
Rodríguez-Nolasco E. 2
Iracheta P. 2
1 HIV Laboratory, HIV Program of Mexico City-Condesa Clinic, Mexico D.F., Mexico
2 HIV Program of Mexico City-Condesa Clinic, Mexico, Mexico
3 Condesa Clinic, Medical Directorate, Mexico, Mexico
§ Presenting author email: luisjuarez@insp.mx
Electronic publication date: 2012 Oct 22
Publication date: 2012
Volume: 15
Issue: Suppl 3
Electronic Location ID: 18440-068
Copyright: © 2012 L. Juárez-Figueroa et al.
Copyright year: 2012
License: This is an open-access article distributed under the terms of the Creative Commons Attribution License, which permits unrestricted use, distribution, and reproduction in any medium, provided the original work is properly cited.
License URL: http://creativecommons.org/licenses/by/2.0/

JOURNAL INFORMATION
==============================
NLM Title Abbreviation: J Int AIDS Soc
Journal ID: J Int AIDS Soc
NLM Journal ID: 101478566
Journal ID: JIAS

Journal of the International AIDS Society

EISSN: 1758-2652
Publisher: Wiley

ARTICLE INFORMATION
==============================
Article ID: 18440-069
Subjects: C26 - Methods for detecting recent HIV infections

MOAC0203: HIV incidence determination in clade B epidemics: a multi-assay approach

Laeyendecker O. 1 2 §
Brookmeyer R. 3
Cousins M. 4
Mullis C. 2
Konikoff J. 3
Donnell D. 5
Celum C. 6
Buchbinder S. 7
Seage G. 8
Kirk G. 9
Mehta S. 9
Astemborski J. 9
Jacobson L. 9
Margolick J. 10
Brown J. 11
Quinn T. 1 2
Eshleman S. 4
1 NIAID, LIR, Baltimore, United States
2 Johns Hopkins University Baltimore Marland, Medicine, Baltimore, United States
3 School of Public Health, University of California at Los Angeles, Department of Biostatistics, Los Angeles, United States
4 Department of Pathology, Johns Hopkins Univ. School of Medicine, Baltimore, United States
5 Fred Hutchinson Cancer Research Center, Vaccine and Infectious Disease Division, Seattle, United States
6 Departments of Global Health and Medicine, University of Washington, Seattle, United States
7 San Francisco Dept. of Health, San Francisco, United States
8 Harvard School of Public Health, Dept. of Epidemiology, Boston, United States
9 Johns Hopkins Bloomberg School of Public Health, Dept. of Epidemiology, Baltimore, United States
10 Johns Hopkins Bloomberg School of Public Health, Dept. of Molecular Microbiology and Immunology, Baltimore, United States
11 School of Public Health, University of California at Los Angeles, Dept of Epidemiology, Los Angeles, United States
§ Presenting author email: olaeyen1@jhmi.edu
Electronic publication date: 2012 Oct 22
Publication date: 2012
Volume: 15
Issue: Suppl 3
Electronic Location ID: 18440-069
Copyright: © 2012 O. Laeyendecker et al.
Copyright year: 2012
License: This is an open-access article distributed under the terms of the Creative Commons Attribution License, which permits unrestricted use, distribution, and reproduction in any medium, provided the original work is properly cited.
License URL: http://creativecommons.org/licenses/by/2.0/

JOURNAL INFORMATION
==============================
NLM Title Abbreviation: J Int AIDS Soc
Journal ID: J Int AIDS Soc
NLM Journal ID: 101478566
Journal ID: JIAS

Journal of the International AIDS Society

EISSN: 1758-2652
Publisher: Wiley

ARTICLE INFORMATION
==============================
Article ID: 18440-070
Subjects: C29 - Determining HIV incidence

FRLBX02: Estimating national HIV incidence from directly observed seroconversions in the Swaziland HIV Incidence Measurement Survey (SHIMS) longitudinal cohort

Reed J.B. 1 §
Justman J. 2
Bicego G. 3
Donnell D. 4
Bock N. 1
Ginindza H. 5
Koler A. 2
Philip N. 2
Makhanya M. 3
Mlambo C. 6
Parekh B.S. 1
Duong Y.T. 1
Ellenberger D.L. 1
Sexton C. 1
Nkambule R. 5
1 U.S. Centers for Disease Control and Prevention, Center for Global Health, Division of Global HIV/AIDS, Atlanta, United States
2 ICAP at the Mailman School of Public Health at Columbia University, New York, United States
3 US Centers for Disease Control and Prevention, Swaziland, Mbabane, Swaziland
4 Fred Hutchinson Cancer Research Center, Seattle, United States
5 Ministry of Health-Swaziland, Mbabane, Swaziland
6 Columbia University, ICAP, Swaziland, Mbabane, Swaziland
§ Presenting author email: eso7@cdc.gov
Electronic publication date: 2012 Oct 22
Publication date: 2012
Volume: 15
Issue: Suppl 3
Electronic Location ID: 18440-070
Copyright: © 2012 J.B. Reed et al.
Copyright year: 2012
License: This is an open-access article distributed under the terms of the Creative Commons Attribution License, which permits unrestricted use, distribution, and reproduction in any medium, provided the original work is properly cited.
License URL: http://creativecommons.org/licenses/by/2.0/

JOURNAL INFORMATION
==============================
NLM Title Abbreviation: J Int AIDS Soc
Journal ID: J Int AIDS Soc
NLM Journal ID: 101478566
Journal ID: JIAS

Journal of the International AIDS Society

EISSN: 1758-2652
Publisher: Wiley

ARTICLE INFORMATION
==============================
Article ID: 18440-071
Subjects: C29 - Determining HIV incidence

MOPDC0206: Incorporating incidence surveillance as part of national HIV surveillance in the United Kingdom: closer tracking of transmission

Aghaizu A. 1
Kall M. 1
Smith R. 1
Tosswill J. 2
Murphy G. 2
Delpech V. 1 §
1 Health Protection Agency, HIV/STI Department, London, United Kingdom
2 Health Protection Agency, Virus Reference Department, London, United Kingdom
§ Presenting author email: valerie.delpech@hpa.org.uk
Electronic publication date: 2012 Oct 22
Publication date: 2012
Volume: 15
Issue: Suppl 3
Electronic Location ID: 18440-071
Copyright: © 2012 V. Delpech et al.
Copyright year: 2012
License: This is an open-access article distributed under the terms of the Creative Commons Attribution License, which permits unrestricted use, distribution, and reproduction in any medium, provided the original work is properly cited.
License URL: http://creativecommons.org/licenses/by/2.0/

JOURNAL INFORMATION
==============================
NLM Title Abbreviation: J Int AIDS Soc
Journal ID: J Int AIDS Soc
NLM Journal ID: 101478566
Journal ID: JIAS

Journal of the International AIDS Society

EISSN: 1758-2652
Publisher: Wiley

ARTICLE INFORMATION
==============================
Article ID: 18440-072
Subjects: C30 - Measuring and modeling the impact of treatment for prevention

THAC0203: The prospects of elimination of HIV with test and treat strategy

Kretzschmar M. 1 2 §
Schim van der Loeff M.F. 3
De Angelis D. 4
Coutinho R.A. 1 2
1 RIVM, Centre for Infectious Disease Control, De Bilt, Netherlands
2 University Medical Center UtrechtJulius Centre for Health Sciences and Primary Care, Utrecht, Netherlands
3 Public Health Service Amsterdam, Division of Infectious Diseases, Amsterdam, Netherlands
4 MRC Biostatistics Unit, Cambridge, United Kingdom
§ Presenting author email: mirjam.kretzschmar@rivm.nl
Electronic publication date: 2012 Oct 22
Publication date: 2012
Volume: 15
Issue: Suppl 3
Electronic Location ID: 18440-072
Copyright: © 2012 M. Kretzschmar et al.
Copyright year: 2012
License: This is an open-access article distributed under the terms of the Creative Commons Attribution License, which permits unrestricted use, distribution, and reproduction in any medium, provided the original work is properly cited.
License URL: http://creativecommons.org/licenses/by/2.0/

JOURNAL INFORMATION
==============================
NLM Title Abbreviation: J Int AIDS Soc
Journal ID: J Int AIDS Soc
NLM Journal ID: 101478566
Journal ID: JIAS

Journal of the International AIDS Society

EISSN: 1758-2652
Publisher: Wiley

ARTICLE INFORMATION
==============================
Article ID: 18440-073
Subjects: C30 - Measuring and modeling the impact of treatment for prevention

THAC0204: Treatment as prevention for HIV in South Africa: different models show consistency in occurrence, but difference in timing of elimination and the overall impact of the intervention

Hontelez J.A.C. 1 2 3 §
Lurie M.N. 4
Bärnighausen T. 3 5
Bakker R. 1
Baltussen R. 2
Tanser F. 3
Hallett T.B. 6
Newell M.-L. 3
de Vlas S.J. 1
1 Erasmus MC, University Medical Center Rotterdam, Public Health, Rotterdam, Netherlands
2 Radboud University Nijmegen Medical CenterNijmegen International Center for Health Systems Research and Education (NICHE), Nijmegen, Netherlands
3 Africa Centre for Health and Population Studies, University of KwaZulu-Natal, Mtubatuba, South Africa
4 Brown University Warren Alpert School of Medicine, Providence, United States
5 Department of Global Health and Population, Harvard School of Public Health, Boston, United States
6 Imperial College London, London, United Kingdom
§ Presenting author email: j.hontelez@erasmusmc.nl
Electronic publication date: 2012 Oct 22
Publication date: 2012
Volume: 15
Issue: Suppl 3
Electronic Location ID: 18440-073
Copyright: © 2012 J.A.C. Hontelez et al.
Copyright year: 2012
License: This is an open-access article distributed under the terms of the Creative Commons Attribution License, which permits unrestricted use, distribution, and reproduction in any medium, provided the original work is properly cited.
License URL: http://creativecommons.org/licenses/by/2.0/

JOURNAL INFORMATION
==============================
NLM Title Abbreviation: J Int AIDS Soc
Journal ID: J Int AIDS Soc
NLM Journal ID: 101478566
Journal ID: JIAS

Journal of the International AIDS Society

EISSN: 1758-2652
Publisher: Wiley

ARTICLE INFORMATION
==============================
Article ID: 18440-074
Subjects: C30 - Measuring and modeling the impact of treatment for prevention

FRLBC01: The cost-effectiveness of treatment as prevention: analysis of the HPTN 052 trial

Walensky R.P. 1 2 3
Ross E.L. 1
Kumarasamy N. 4
Wood R. 5
Noubary F. 1
Paltiel A.D. 6
Nakamura Y.M. 1
Godbole S. 7
Hosseinipour M. 8
Hakim J.G. 9
Kumwenda J. 10
Makhema J. 11
Akelo V. 12
Panchia R. 13
Sanne I. 14
Weinstein M.C. 15
Losina E. 3 16 17
Mayer K.H. 18
Grinsztejn B. 19
Pilotto J. 20
Chariyalertsak S. 21
Santos B. 22
Chen Y.Q. 23
Wang L. 23
Li X. 23
McCauley M. 24
Gamble T. 25
Piwowar-Manning E. 26
Cottle L. 27
Hoffman I. 28
Eron J. 28
Gallant J. 26
Swindells S. 29
Taha T. 30
Nielsen-Saines K. 31
Celentano D. 30
Essex M. 15
Elharrar V. 32
Burns D. 32
Seage G.R. 15
Cohen M.S. 28
Freedberg K.A. 1 2 16 §
1 Massachusetts General Hospital, Boston, United States
2 Harvard Medical School, Boston, United States
3 Brigham & Women's Hospital, Boston, United States
4 Y. R. Gaitonade Center for AIDS Research and Education, Chennai, India
5 University of Cape Town and Desmond Tutu HIV centre, Cape Town, South Africa
6 Yale University School of Medicine, New Haven, United States
7 National AIDS Research Institute (NARI), Pune, India
8 UNC Project Malawi and University of North Carolina at Chapel Hill, Lilongwe, Malawi
9 Department of Medicine, University of Zimbabwe, Harare, Zimbabwe
10 College of Medicine Johns Hopkins Project, Blantyre, Malawi
11 Botswana Harvard AIDS Institute, Gaborone, Botswana
12 KEMRI-CDC Research and Public Health Collaboration, Kisumu, Kenya
13 Perinatal HIV Research Unit (PHRU)University of the Witwatersrand, Johannesburg, South Africa
14 University of WitswatersrandDepartment of Medicine, Johannesburg, South Africa
15 Harvard School of Public Health, Boston, United States
16 Boston University School of Public Health, Boston, United States
17 Harvard Center for AIDS Research, Boston, United States
18 Fenway Institute, Boston, United States
19 Instituto de Pesquisa Clinica Evandro Chagas, Fiocruz, Rio de Janeiro, Brazil
20 Hospital Geral de Nova Iguacu and Laboratorio de AIDS e Imunologia Molecular-IOC/Fiocruz, Rio de Janeiro, Brazil
21 Research Institute for Health SciencesChiang Mai University, Chiang Mai, Thailand
22 Hospital Nossa Senhora da Conceição, Porto Alegre, Brazil
23 Vaccine and Infectious Disease Division, Fred Hutchinson Cancer Research Center, Seattle, United States
24 FHI 360, Washington, United States
25 FHI 360, Durham, United States
26 Johns Hopkins School of Medicine, Baltimore, United States
27 Statistical Center for HIV/AIDS Research & Prevention (SCHARP), Seattle, United States
28 University of North Carolina at Chapel Hill, School of Medicine, Chapel Hill, United States
29 University of Nebraska Medical Center, Omaha, United States
30 Johns Hopkins Bloomberg School of Public Health, Baltimore, United States
31 Division of Infectious Diseases, David Geffen School of Medicine, University of California, Los Angeles, United States
32 National Institute of Allergy and Infectious DiseasesNational Institutes of Health, Bethesda, United States
§ Presenting author email: kfreedberg@partners.org
Electronic publication date: 2012 Oct 22
Publication date: 2012
Volume: 15
Issue: Suppl 3
Electronic Location ID: 18440-074
Copyright: © 2012 K.A. Freedberg et al.
Copyright year: 2012
License: This is an open-access article distributed under the terms of the Creative Commons Attribution License, which permits unrestricted use, distribution, and reproduction in any medium, provided the original work is properly cited.
License URL: http://creativecommons.org/licenses/by/2.0/

JOURNAL INFORMATION
==============================
NLM Title Abbreviation: J Int AIDS Soc
Journal ID: J Int AIDS Soc
NLM Journal ID: 101478566
Journal ID: JIAS

Journal of the International AIDS Society

EISSN: 1758-2652
Publisher: Wiley

ARTICLE INFORMATION
==============================
Article ID: 18440-075
Subjects: C30 - Measuring and modeling the impact of treatment for prevention

MOPDC0101: The effects of different ART eligibility strategies on HIV-related morality and incidence

Stover J. 1 §
Pretorius C. 1
1 Futures Institute, Glastonbury, United States
§ Presenting author email: jstover@futuresinstitute.org
Electronic publication date: 2012 Oct 22
Publication date: 2012
Volume: 15
Issue: Suppl 3
Electronic Location ID: 18440-075
Copyright: © 2012 J. Stover et al.
Copyright year: 2012
License: This is an open-access article distributed under the terms of the Creative Commons Attribution License, which permits unrestricted use, distribution, and reproduction in any medium, provided the original work is properly cited.
License URL: http://creativecommons.org/licenses/by/2.0/

JOURNAL INFORMATION
==============================
NLM Title Abbreviation: J Int AIDS Soc
Journal ID: J Int AIDS Soc
NLM Journal ID: 101478566
Journal ID: JIAS

Journal of the International AIDS Society

EISSN: 1758-2652
Publisher: Wiley

ARTICLE INFORMATION
==============================
Article ID: 18440-076
Subjects: C30 - Measuring and modeling the impact of treatment for prevention

MOPDC0103: Modelling potential preventive impact of expanded antiretroviral therapy, early antiretroviral therapy for serodiscordant couples and harm reduction interventions in a concentrated epidemic in Viet Nam

Kato M. 1 §
Granich R. 2
Tran Vu H. 3
Nadol P. 4
Sabin K. 1
Suthar A. 2
Williams B. 5
1 World Health OrganizationViet Nam Country Office, Hanoi, Viet Nam
2 HIV Department, World Health Organization, Geneva, Switzerland
3 Partners in Health Research, Hanoi, Viet Nam
4 Center for Disease Control and PreventionViet Nam Country Office, Hanoi, Viet Nam
5 South African Centre for Epidemiological Modelling and Analysis (SACEMA), Geneva, Switzerland
§ Presenting author email: katom@wpro.who.int
Electronic publication date: 2012 Oct 22
Publication date: 2012
Volume: 15
Issue: Suppl 3
Electronic Location ID: 18440-076
Copyright: © 2012 M. Kato et al.
Copyright year: 2012
License: This is an open-access article distributed under the terms of the Creative Commons Attribution License, which permits unrestricted use, distribution, and reproduction in any medium, provided the original work is properly cited.
License URL: http://creativecommons.org/licenses/by/2.0/

JOURNAL INFORMATION
==============================
NLM Title Abbreviation: J Int AIDS Soc
Journal ID: J Int AIDS Soc
NLM Journal ID: 101478566
Journal ID: JIAS

Journal of the International AIDS Society

EISSN: 1758-2652
Publisher: Wiley

ARTICLE INFORMATION
==============================
Article ID: 18440-077
Subjects: C30 - Measuring and modeling the impact of treatment for prevention

MOPDC0106: Sustained treatment as prevention: continued decreases in unprotected sex and increases in virological suppression after HAART initiation among participants in HPTN 052

Mayer K. 1 §
Wang L. 2
Hoffman I. 3
McCauley M. 4
Li X. 2
Safren S. 5
Gamble T. 4
Talley J. 4
Cottle L. 6
Piwowar-Manning E. 7
Akelo V. 8
Badal-Faesen S. 9
Chotirosniramit N. 10
Fernandes N.M. 11
Kumarasamy N. 12
Sahay S. 13
Makhema J. 14
Panchia B. 15
Pilotto J.H.d.S. 16
Santos B.R. 17
Cohen M.S. 18
1 Fenway Community Health, Boston, United States
2 Vaccine and Infectious Disease Division, Fred Hutchinson Cancer Research Center, Seattle, United States
3 Department of Medicine, University of North Carolina-Chapel Hill, Chapel Hill, United States
4 FHI 360, Washington, United States
5 Harvard Medical School/Massachusetts General Hospital and Fenway Health, Boston, United States
6 Statistical Center for HIV/AIDS Research & Prevention (SCHARP), Seattle, United States
7 Pathology Department, Johns Hopkins University School of Medicine, Baltimore, United States
8 Kenya Medical Research Institute Center for Global Health Research, Kisumu, Kenya
9 University of the WitwatersrandClinical HIV Research Unit, Johannesburg, South Africa
10 Chiang Mai UniversityResearch Institute for Health Sciences, Chiang Mai, Thailand
11 Instituto de Pesquisa Clinica Evandro Chagas, Rio de Janeiro, Brazil
12 YRG CARE Medical Centre, VHS, Chennai, India
13 National AIDS Research Institute (ICMR), Pune, India
14 Botswana Harvard AIDS Institute, Gabarone, Botswana
15 University of the Witwatersrand, Perinatal HIV Research Unit, Johannesburg, South Africa
16 Hospital Geral de Nova Iguacu and Laboratorio de AIDS e Imunologia Molecular-IOC/Fiocruz, Rio de Janeiro, Brazil
17 Hospital Nossa Senhora da Conceição, Porto Alegre, Brazil
18 Department of Medicine, University of North Carolina School of Medicine, Chapel Hill, United States
§ Presenting author email: kmayer@fenwayhealth.org
Electronic publication date: 2012 Oct 22
Publication date: 2012
Volume: 15
Issue: Suppl 3
Electronic Location ID: 18440-077
Copyright: © 2012 K. Mayer et al.
Copyright year: 2012
License: This is an open-access article distributed under the terms of the Creative Commons Attribution License, which permits unrestricted use, distribution, and reproduction in any medium, provided the original work is properly cited.
License URL: http://creativecommons.org/licenses/by/2.0/

JOURNAL INFORMATION
==============================
NLM Title Abbreviation: J Int AIDS Soc
Journal ID: J Int AIDS Soc
NLM Journal ID: 101478566
Journal ID: JIAS

Journal of the International AIDS Society

EISSN: 1758-2652
Publisher: Wiley

ARTICLE INFORMATION
==============================
Article ID: 18440-078
Subjects: C35 - Behavioural surveillance

MOPDC0205: Sexual behaviour trends by gender in a rural South African population-based cohort during the era of scaled-up access to VCT and ART, 2005–2010

McGrath N. 1 2 §
Eaton J.W. 3
Tanser F. 1
Bärnighausen T. 1 4
Newell M.-L. 1 5
1 Africa Centre for Health and Population StudiesUniversity of KwaZulu-Natal, Mtubatuba, South Africa
2 London School of Hygiene and Tropical MedicineInfectious Disease Epidemiology, London, United Kingdom
3 Imperial CollegeInfectious Disease Epidemiology, London, United Kingdom
4 Harvard School of Public HealthGlobal Health and Population, Boston, United States
5 Institute of Child HealthUniversity College London (UCL), Centre for Paediatric Epidemiology and Biostatistics, London, United Kingdom
§ Presenting author email: nuala.mcgrath@lshtm.ac.uk
Electronic publication date: 2012 Oct 22
Publication date: 2012
Volume: 15
Issue: Suppl 3
Electronic Location ID: 18440-078
Copyright: © 2012 N. McGrath et al.
Copyright year: 2012
License: This is an open-access article distributed under the terms of the Creative Commons Attribution License, which permits unrestricted use, distribution, and reproduction in any medium, provided the original work is properly cited.
License URL: http://creativecommons.org/licenses/by/2.0/

JOURNAL INFORMATION
==============================
NLM Title Abbreviation: J Int AIDS Soc
Journal ID: J Int AIDS Soc
NLM Journal ID: 101478566
Journal ID: JIAS

Journal of the International AIDS Society

EISSN: 1758-2652
Publisher: Wiley

ARTICLE INFORMATION
==============================
Article ID: 18440-079
Subjects: C36 - Surveillance of HIV drug resistance

MOAC0303: The World Health Organization's HIV drug resistance early warning indicators: results from 50 countries 2004–2009

Bennett D.E. 1 §
Jordan M.R. 2 3
Bertagnolio S. 2
Hong S.Y. 3
Ravasi G. 4
McMahon J.H. 3 5
Kelley K.F. 6
1 US Centers for Disease Control & Prevention, CGH DGHA, Atlanta, United States
2 Department of HIV/AIDS, World Health Organization, Geneva, Switzerland
3 Tufts University School of Medicine, Boston, United States
4 Pan American Health Organization, Brasilia, Brazil
5 Department of Infectious Disease, Alfred Hospital, Melbourne, Australia
6 U.S.President's Emergency Plan for AIDS Relief, New Delhi, India
§ Presenting author email: dib1@cdc.gov
Electronic publication date: 2012 Oct 22
Publication date: 2012
Volume: 15
Issue: Suppl 3
Electronic Location ID: 18440-079
Copyright: © 2012 D.E. Bennett et al.
Copyright year: 2012
License: This is an open-access article distributed under the terms of the Creative Commons Attribution License, which permits unrestricted use, distribution, and reproduction in any medium, provided the original work is properly cited.
License URL: http://creativecommons.org/licenses/by/2.0/

JOURNAL INFORMATION
==============================
NLM Title Abbreviation: J Int AIDS Soc
Journal ID: J Int AIDS Soc
NLM Journal ID: 101478566
Journal ID: JIAS

Journal of the International AIDS Society

EISSN: 1758-2652
Publisher: Wiley

ARTICLE INFORMATION
==============================
Article ID: 18440-080
Subjects: C40 - Monitoring and evaluation of HIV prevention, care and treatment

MOAC0302: Effects of patient tracing on estimates of lost to follow-up, mortality and retention in antiretroviral therapy programs in low- and middle-income countries: a systematic review

McMahon J. 1 2
Elliott J. 1 3 4
Hong S. 2 5
Jordan M. 2 5
1 Alfred Hospital, Infectious Diseases Unit, Melbourne, Australia
2 Department of Public Health and Community Medicine, Tufts University School of Medicine, Boston, United States
3 Department of Medicine, Monash University, Melbourne, Australia
4 Burnet InstituteCentre for Population Health, Melbourne, Australia
5 Department of Geographic Medicine and Infectious Diseases, Tufts Medical Center, Boston, Australia
Electronic publication date: 2012 Oct 22
Publication date: 2012
Volume: 15
Issue: Suppl 3
Electronic Location ID: 18440-080
Copyright: © 2012 et al.
Copyright year: 2012
License: This is an open-access article distributed under the terms of the Creative Commons Attribution License, which permits unrestricted use, distribution, and reproduction in any medium, provided the original work is properly cited.
License URL: http://creativecommons.org/licenses/by/2.0/

JOURNAL INFORMATION
==============================
NLM Title Abbreviation: J Int AIDS Soc
Journal ID: J Int AIDS Soc
NLM Journal ID: 101478566
Journal ID: JIAS

Journal of the International AIDS Society

EISSN: 1758-2652
Publisher: Wiley

ARTICLE INFORMATION
==============================
Article ID: 18440-081
Subjects: C40 - Monitoring and evaluation of HIV prevention, care and treatment

MOAC0304: CD4 criteria improves the sensitivity of a clinical algorithm developed to identify viral failure in HIV-positive patients on first-line antiretroviral therapy

Evans D.H. 1
Maskew M. 1
Fox M. 2 §
McNamara L. 1
MacPhail P. 3 4
Mathews C. 5
Sanne I. 3 4
1 Department of Medicine, Faculty of Health Sciences, University of the Witwatersrand (WITS), Health Economics and Epidemiology Research Office, Johannesburg, South Africa
2 Boston University, Center for Global Health and Development, Boston, United States
3 Department of Medicine, Faculty of Health Sciences, University of the Witwatersrand (WITS), Clinical HIV Research Unit, Johannnesburg, South Africa
4 Right to Care, Johannnesburg, South Africa
5 Department of Medicine, University of California, San Diego, United States
§ Presenting author email: mfox@bu.edu
Electronic publication date: 2012 Oct 22
Publication date: 2012
Volume: 15
Issue: Suppl 3
Electronic Location ID: 18440-081
Copyright: © 2012 M. Fox et al.
Copyright year: 2012
License: This is an open-access article distributed under the terms of the Creative Commons Attribution License, which permits unrestricted use, distribution, and reproduction in any medium, provided the original work is properly cited.
License URL: http://creativecommons.org/licenses/by/2.0/

JOURNAL INFORMATION
==============================
NLM Title Abbreviation: J Int AIDS Soc
Journal ID: J Int AIDS Soc
NLM Journal ID: 101478566
Journal ID: JIAS

Journal of the International AIDS Society

EISSN: 1758-2652
Publisher: Wiley

ARTICLE INFORMATION
==============================
Article ID: 18440-082
Subjects: C40 - Monitoring and evaluation of HIV prevention, care and treatment

MOAC0305: Retention and risk factors for attrition among adults in antiretroviral treatment (ART) programs in Tanzania, Uganda and Zambia

Koole O. 1 §
Tsui S. 2
Wabwire-Mangen F. 3
Kwesigabo G. 4
Menten J. 1
Mulenga M. 5
Auld A. 6
Agolory S. 6
Mukadi Y.D. 7
Colebunders R. 1
Bangsberg D.R. 8
Van Praag E. 9
Torpey K. 10
Williams S. 6
Kaplan J. 6
Zee A. 6
Denison J. 11
1 Institute of Tropical Medicine, Clinical Sciences, Antwerp, Belgium
2 FHI 360, Behavioral and Social Sciences, Durham, United States
3 Infectious Diseases Institute, Makerere University College of Health Sciences, Kampala, Uganda
4 Muhimbili University of Health and Allied Sciences, Dar es Salaam, United Republic of Tanzania
5 Tropical Diseases Research Centre, Ndola, Zambia
6 Centers for Disease Control and Prevention, Division of Global AIDS, Atlanta, United States
7 Formerly FHI 360, Washington, United States
8 Massachusetts General Hospital, Boston, United States
9 FHI 360, Dar es Salaam, United Republic of Tanzania
10 FHI 360, Lagos, Nigeria
11 FHI 360, Behavioral and Social Sciences, Washington, United States
§ Presenting author email: okoole@itg.be
Electronic publication date: 2012 Oct 22
Publication date: 2012
Volume: 15
Issue: Suppl 3
Electronic Location ID: 18440-082
Copyright: © 2012 O. Koole et al.
Copyright year: 2012
License: This is an open-access article distributed under the terms of the Creative Commons Attribution License, which permits unrestricted use, distribution, and reproduction in any medium, provided the original work is properly cited.
License URL: http://creativecommons.org/licenses/by/2.0/

JOURNAL INFORMATION
==============================
NLM Title Abbreviation: J Int AIDS Soc
Journal ID: J Int AIDS Soc
NLM Journal ID: 101478566
Journal ID: JIAS

Journal of the International AIDS Society

EISSN: 1758-2652
Publisher: Wiley

ARTICLE INFORMATION
==============================
Article ID: 18440-083
Subjects: C40 - Monitoring and evaluation of HIV prevention, care and treatment

FRLBC02: HIV clinical and program outcomes among older patients with HIV enrolled in HIV care and initiating ART in sub-Saharan Africa

Lamb M.R. 1 §
Eduardo E. 2
Kandula S. 1
Howard A. 1 2
El Sadr W. 1 2 3
Mugisha V. 4
Kimanga D. 5
Kilama B. 6
Elul B. 1 2
1 Mailman School of Public Health, Columbia University, ICAP, New York, United States
2 Mailman School of Public Health, Columbia University, Epidemiology, New York, United States
3 Columbia University College of Physicians & Surgeons, New York, United States
4 ICAP-Rwanda, Kigali, Rwanda
5 National AIDS and STI Control Programme, Nairobi, Kenya
6 National AIDS Control Programme, Dar es Salaam, United Republic of Tanzania
§ Presenting author email: mrl2013@columbia.edu
Electronic publication date: 2012 Oct 22
Publication date: 2012
Volume: 15
Issue: Suppl 3
Electronic Location ID: 18440-083
Copyright: © 2012 M.R. Lamb et al.
Copyright year: 2012
License: This is an open-access article distributed under the terms of the Creative Commons Attribution License, which permits unrestricted use, distribution, and reproduction in any medium, provided the original work is properly cited.
License URL: http://creativecommons.org/licenses/by/2.0/

JOURNAL INFORMATION
==============================
NLM Title Abbreviation: J Int AIDS Soc
Journal ID: J Int AIDS Soc
NLM Journal ID: 101478566
Journal ID: JIAS

Journal of the International AIDS Society

EISSN: 1758-2652
Publisher: Wiley

ARTICLE INFORMATION
==============================
Article ID: 18440-084
Subjects: C40 - Monitoring and evaluation of HIV prevention, care and treatment

MOPDC0301: The United Kingdom's National Health Service (NHS) provides excellent access to high quality HIV care: results from a national cohort

Delpech V. 1 §
Yin Z. 1
Kall M. 1
Brown A. 1
1 Heatlh Protection Agency, London, United Kingdom
§ Presenting author email: valerie.delpech@hpa.org.uk
Electronic publication date: 2012 Oct 22
Publication date: 2012
Volume: 15
Issue: Suppl 3
Electronic Location ID: 18440-084
Copyright: © 2012 V. Delpech et al.
Copyright year: 2012
License: This is an open-access article distributed under the terms of the Creative Commons Attribution License, which permits unrestricted use, distribution, and reproduction in any medium, provided the original work is properly cited.
License URL: http://creativecommons.org/licenses/by/2.0/

JOURNAL INFORMATION
==============================
NLM Title Abbreviation: J Int AIDS Soc
Journal ID: J Int AIDS Soc
NLM Journal ID: 101478566
Journal ID: JIAS

Journal of the International AIDS Society

EISSN: 1758-2652
Publisher: Wiley

ARTICLE INFORMATION
==============================
Article ID: 18440-085
Subjects: C40 - Monitoring and evaluation of HIV prevention, care and treatment

MOPDC0303: Linkage, retention, ART use and viral suppression in four large cities in the United States

Benbow N. 1 §
Scheer S. 2
Wohl A. 3
Brady K. 4
Gagner A. 1
Hughes A. 2
Tejero J. 3
Eberhart M. 4
Hu V. 3
Sayles J. 3
Townsell S. 1
1 Chicago Department of Public Health, Chicago, United States
2 San Francisco Department of Public Health, San Francisco, United States
3 Los Angeles County Department of Public Health, Los Angeles, United States
4 Philadelphia Department of Public Health, Philadelphia, United States
§ Presenting author email: n.benbow@gmail.com
Electronic publication date: 2012 Oct 22
Publication date: 2012
Volume: 15
Issue: Suppl 3
Electronic Location ID: 18440-085
Copyright: © 2012 N. Benbow et al.
Copyright year: 2012
License: This is an open-access article distributed under the terms of the Creative Commons Attribution License, which permits unrestricted use, distribution, and reproduction in any medium, provided the original work is properly cited.
License URL: http://creativecommons.org/licenses/by/2.0/

JOURNAL INFORMATION
==============================
NLM Title Abbreviation: J Int AIDS Soc
Journal ID: J Int AIDS Soc
NLM Journal ID: 101478566
Journal ID: JIAS

Journal of the International AIDS Society

EISSN: 1758-2652
Publisher: Wiley

ARTICLE INFORMATION
==============================
Article ID: 18440-086
Subjects: C40 - Monitoring and evaluation of HIV prevention, care and treatment

MOPDC0304: Factors associated with achieving viral suppression among newly diagnosed HIV/AIDS cases in the Washington, D.C.

Willis S. 1 §
Castel A. 1
Griffin A. 2
West T. 2
Shaikh I. 2
Pappas G. 2
1 The George Washington University, Department of Epidemiology and Biostatistics, Washington, United States
2 District of Columbia Department of Health, HIV/AIDS, Hepatitis, STD and TB Administration, Washington, United States
§ Presenting author email: sarah.willis2@dc.gov
Electronic publication date: 2012 Oct 22
Publication date: 2012
Volume: 15
Issue: Suppl 3
Electronic Location ID: 18440-086
Copyright: © 2012 S. Willis et al.
Copyright year: 2012
License: This is an open-access article distributed under the terms of the Creative Commons Attribution License, which permits unrestricted use, distribution, and reproduction in any medium, provided the original work is properly cited.
License URL: http://creativecommons.org/licenses/by/2.0/

JOURNAL INFORMATION
==============================
NLM Title Abbreviation: J Int AIDS Soc
Journal ID: J Int AIDS Soc
NLM Journal ID: 101478566
Journal ID: JIAS

Journal of the International AIDS Society

EISSN: 1758-2652
Publisher: Wiley

ARTICLE INFORMATION
==============================
Article ID: 18440-087
Subjects: C40 - Monitoring and evaluation of HIV prevention, care and treatment

WEPDC0202: Evaluation of a behaviour change communication programme to reduce concurrent sexual partnerships in Botswana

Halldorsdottir I. 1
Taruberekera N. 2
Capo-Chichi V. 3
Firestone R. 4 §
Vu L. 4
Sherard D. 5
Harrison R. 1
1 PSI/Botswana, Gaborone, Botswana
2 PSI, Johannesburg, South Africa
3 PSI, Research & Metrics, Coutonou, Benin
4 PSI, Research & Metrics, Washington, United States
5 PSI, Sexual, Reproductive Health and TB Department, Washington, United States
§ Presenting author email: iris.halldorsdottir@psi.co.bw
Electronic publication date: 2012 Oct 22
Publication date: 2012
Volume: 15
Issue: Suppl 3
Electronic Location ID: 18440-087
Copyright: © 2012 R. Firestone et al.
Copyright year: 2012
License: This is an open-access article distributed under the terms of the Creative Commons Attribution License, which permits unrestricted use, distribution, and reproduction in any medium, provided the original work is properly cited.
License URL: http://creativecommons.org/licenses/by/2.0/

JOURNAL INFORMATION
==============================
NLM Title Abbreviation: J Int AIDS Soc
Journal ID: J Int AIDS Soc
NLM Journal ID: 101478566
Journal ID: JIAS

Journal of the International AIDS Society

EISSN: 1758-2652
Publisher: Wiley

ARTICLE INFORMATION
==============================
Article ID: 18440-088
Subjects: C41 - Monitoring and evaluation (other)

TUAC0205: Evaluating the use of HIV surveillance data for initiating partner services in Houston, Texas, U.S.

Chan S. 1 §
Yang B. 1
Wolverton M. 1
Arafat R. 1
1 City of Houston, Department of Health and Human Services, Houston, United States
§ Presenting author email: shirley.chan@houstontx.gov
Electronic publication date: 2012 Oct 22
Publication date: 2012
Volume: 15
Issue: Suppl 3
Electronic Location ID: 18440-088
Copyright: © 2012 S. Chan et al.
Copyright year: 2012
License: This is an open-access article distributed under the terms of the Creative Commons Attribution License, which permits unrestricted use, distribution, and reproduction in any medium, provided the original work is properly cited.
License URL: http://creativecommons.org/licenses/by/2.0/

JOURNAL INFORMATION
==============================
NLM Title Abbreviation: J Int AIDS Soc
Journal ID: J Int AIDS Soc
NLM Journal ID: 101478566
Journal ID: JIAS

Journal of the International AIDS Society

EISSN: 1758-2652
Publisher: Wiley

ARTICLE INFORMATION
==============================
Article ID: 18440-089
Subjects: C41 - Monitoring and evaluation (other)

MOPDC0302: Trends in antiretroviral therapy use, HIV RNA plasma viral load and CD4 T-lymphocyte counts at death among HIV-positive persons in care in the United States, 2000–2008

Althoff K.N. 1 §
Buchacz K. 2
Hall I. 2
Zhang J. 1
Hanna D.B. 1
Rebeiro P. 1
Gange S.J. 1
Moore R.D. 1
Kitahata M. 3
Gebo K.A. 1
Martin J. 4
Justice A.C. 5
Horberg M. 6
Hogg R.S. 7
Sterling T.R. 8
Cescon A. 7
Klein M.B. 9
Thorne J. 1
Crane H. 3
Mugavero M.J. 10
Napravnik S. 11
Kirk G.D. 1
Jacobson L.P. 1
Rodriguez B. 12
Brooks J.T. 2
North American AIDS Cohort Collaboration on Research and Design
1 Johns Hopkins University, Baltimore, United States
2 Centers for Disease Control and Prevention (CDC), Atlanta, United States
3 University of Washington, Seattle, United States
4 University of California at San Francisco, San Francisco, United States
5 Yale University and the VA Connecticut Healthcare System, New Haven, United States
6 Mid-Atlantic Permanente Research Institute, Rockville, United States
7 BC Centre for Excellence in HIV/AIDS and Simon Fraser University, Vancouver, Canada
8 Vanderbilt University, Nashville, United States
9 McGill University, Montréal, Canada
10 University of Alabama, Birmingham, United States
11 University of North Carolina, Chapel Hill, United States
12 Case Western Reserve University, Cleveland, United States
§ Presenting author email: kalthoff@jhsph.edu
Electronic publication date: 2012 Oct 22
Publication date: 2012
Volume: 15
Issue: Suppl 3
Electronic Location ID: 18440-089
Copyright: © 2012 K.N. Althoff et al.
Copyright year: 2012
License: This is an open-access article distributed under the terms of the Creative Commons Attribution License, which permits unrestricted use, distribution, and reproduction in any medium, provided the original work is properly cited.
License URL: http://creativecommons.org/licenses/by/2.0/

JOURNAL INFORMATION
==============================
NLM Title Abbreviation: J Int AIDS Soc
Journal ID: J Int AIDS Soc
NLM Journal ID: 101478566
Journal ID: JIAS

Journal of the International AIDS Society

EISSN: 1758-2652
Publisher: Wiley

ARTICLE INFORMATION
==============================
Article ID: 18440-090
Subjects: C42 - Evaluation of health systems' response to HIV

TUAC0204: Is HIV mother-to-child transmission and congenital syphilis elimination by 2015 a reality in the English Caribbean?

Gebre Y. 1 §
Jack N. 2
Edwards P. 2
Del Riego A. 2
Caffe S. 3
1 Pan American Health Organization/World Health Organization, Family & Community Health, Port of Spain, Trinidad and Tobago
2 Pan American Health Organization, HIV Caribbean Office, Port of Spain, Trinidad and Tobago
3 Pan American Health Organization, HIV/STI Project, HIV/AIDS, Washington, United States
§ Presenting author email: gebrey@gmail.com
Electronic publication date: 2012 Oct 22
Publication date: 2012
Volume: 15
Issue: Suppl 3
Electronic Location ID: 18440-090
Copyright: © 2012 Y. Gebre et al.
Copyright year: 2012
License: This is an open-access article distributed under the terms of the Creative Commons Attribution License, which permits unrestricted use, distribution, and reproduction in any medium, provided the original work is properly cited.
License URL: http://creativecommons.org/licenses/by/2.0/

JOURNAL INFORMATION
==============================
NLM Title Abbreviation: J Int AIDS Soc
Journal ID: J Int AIDS Soc
NLM Journal ID: 101478566
Journal ID: JIAS

Journal of the International AIDS Society

EISSN: 1758-2652
Publisher: Wiley

ARTICLE INFORMATION
==============================
Article ID: 18440-091
Subjects: C42 - Evaluation of health systems' response to HIV

TUPDC0104: Assessing quality of services provided by community-preferred private providers for sexually transmitted infections in a targeted HIV prevention program in Maharashtra, India

Koul R. 1 §
Vyas D. 1
1 Pathfinder International/India, Pune, India
§ Presenting author email: rkoul@pathfinder.org
Electronic publication date: 2012 Oct 22
Publication date: 2012
Volume: 15
Issue: Suppl 3
Electronic Location ID: 18440-091
Copyright: © 2012 R. Koul et al.
Copyright year: 2012
License: This is an open-access article distributed under the terms of the Creative Commons Attribution License, which permits unrestricted use, distribution, and reproduction in any medium, provided the original work is properly cited.
License URL: http://creativecommons.org/licenses/by/2.0/

JOURNAL INFORMATION
==============================
NLM Title Abbreviation: J Int AIDS Soc
Journal ID: J Int AIDS Soc
NLM Journal ID: 101478566
Journal ID: JIAS

Journal of the International AIDS Society

EISSN: 1758-2652
Publisher: Wiley

ARTICLE INFORMATION
==============================
Article ID: 18440-092
Subjects: C43 - Surveillance and monitoring of HIV/hepatitis C (HCV) co-infection

MOAC0402: A critical need to scale-up of HIV prevention and harm reduction services for people who inject drugs in Tanzaniaresults from a HIV and hepatitis C prevalence study in Dar es Salaam, 2011

Stoové M. 1 2 §
Bowring A. 1
Luhmann N. 3
Debaulieu C. 4
Stéphanie D. 3
Pont S. 4
Assouab F. 3
Abdalla T. 3
van Gemert C. 1
Dietze P. 1
1 Burnet Institute, Centre for Population Health, Melbourne, Australia
2 Monash University, Epidemiology and Preventive Medicine, Melbourne, Australia
3 Médecins du Monde, Paris, France
4 Médecins du Monde, Dar es Salaam, United Republic of Tanzania
§ Presenting author email: stoove@burnet.edu.au
Electronic publication date: 2012 Oct 22
Publication date: 2012
Volume: 15
Issue: Suppl 3
Electronic Location ID: 18440-092
Copyright: © 2012 M. Stoové et al.
Copyright year: 2012
License: This is an open-access article distributed under the terms of the Creative Commons Attribution License, which permits unrestricted use, distribution, and reproduction in any medium, provided the original work is properly cited.
License URL: http://creativecommons.org/licenses/by/2.0/

JOURNAL INFORMATION
==============================
NLM Title Abbreviation: J Int AIDS Soc
Journal ID: J Int AIDS Soc
NLM Journal ID: 101478566
Journal ID: JIAS

Journal of the International AIDS Society

EISSN: 1758-2652
Publisher: Wiley

ARTICLE INFORMATION
==============================
Article ID: 18440-093
Subjects: C45 - Prevention of mother-to-child transmission

THAC0103: Will your partner be attending? Involving men in the prevention of mother-to-child transmission of HIV in antenatal care clinics in Iringa, Tanzania

Kikumbih N. 1
Nielsen-Bobbit J. 2
Mbandi A. 1
Motta W. 1
Killian R. 1
Mwanga F. 1
1 EngenderHealth, ACQUIRE Tanzania Project, Dar es SalaamUnited Republic of Tanzania
2 EngenderHealth, Dar es Salaam, United Republic of Tanzania
Electronic publication date: 2012 Oct 22
Publication date: 2012
Volume: 15
Issue: Suppl 3
Electronic Location ID: 18440-093
Copyright: © 2012 et al.
Copyright year: 2012
License: This is an open-access article distributed under the terms of the Creative Commons Attribution License, which permits unrestricted use, distribution, and reproduction in any medium, provided the original work is properly cited.
License URL: http://creativecommons.org/licenses/by/2.0/

JOURNAL INFORMATION
==============================
NLM Title Abbreviation: J Int AIDS Soc
Journal ID: J Int AIDS Soc
NLM Journal ID: 101478566
Journal ID: JIAS

Journal of the International AIDS Society

EISSN: 1758-2652
Publisher: Wiley

ARTICLE INFORMATION
==============================
Article ID: 18440-094
Subjects: C45 - Prevention of mother-to-child transmission

THAC0104: HIV seroconversion during pregnancy and mother-to-child HIV transmission: data from the Enhanced Perinatal Surveillance Project, United States, 2005–2010

Singh S. 1 §
Lampe M.A. 1
Surendera Babu A. 2
Rao S. 1
Borkowf C.B. 1
Nesheim S.R. 1
1 Centers for Disease Control and Prevention, Division of HIV/AIDS Prevention, Atlanta, United States
2 ICF International, Atlanta, United States
§ Presenting author email: fyv7@cdc.gov
Electronic publication date: 2012 Oct 22
Publication date: 2012
Volume: 15
Issue: Suppl 3
Electronic Location ID: 18440-094
Copyright: © 2012 S. Singh et al.
Copyright year: 2012
License: This is an open-access article distributed under the terms of the Creative Commons Attribution License, which permits unrestricted use, distribution, and reproduction in any medium, provided the original work is properly cited.
License URL: http://creativecommons.org/licenses/by/2.0/

JOURNAL INFORMATION
==============================
NLM Title Abbreviation: J Int AIDS Soc
Journal ID: J Int AIDS Soc
NLM Journal ID: 101478566
Journal ID: JIAS

Journal of the International AIDS Society

EISSN: 1758-2652
Publisher: Wiley

ARTICLE INFORMATION
==============================
Article ID: 18440-095
Subjects: C45 - Prevention of mother-to-child transmission

THAC0105: CD4 assessment among newly diagnosed HIV-positive pregnant women in India's national prevention of mother-to-child transmission (PMTCT) programmeimplications for a 'test and treat' approach

Mohammed S.K. 1 §
Gupta R.S. 2
Rao R. 2
Joseph V. 2
Srikantiah P. 3
1 Reproductive and Child Health Division, Ministry of Health and Family Welfare, Government of India, New Delhi, India
2 National AIDS Control Organisation, Department of AIDS Control, Ministry of Health and Family Welfare, Government of India, New Delhi, India
3 University of California, San Francisco, HIV/AIDS Division, San Francisco General Hospital, San Francisco, United States
§ Presenting author email: sureshmohammed@gmail.com
Electronic publication date: 2012 Oct 22
Publication date: 2012
Volume: 15
Issue: Suppl 3
Electronic Location ID: 18440-095
Copyright: © 2012 S.K. Mohammed et al.
Copyright year: 2012
License: This is an open-access article distributed under the terms of the Creative Commons Attribution License, which permits unrestricted use, distribution, and reproduction in any medium, provided the original work is properly cited.
License URL: http://creativecommons.org/licenses/by/2.0/

JOURNAL INFORMATION
==============================
NLM Title Abbreviation: J Int AIDS Soc
Journal ID: J Int AIDS Soc
NLM Journal ID: 101478566
Journal ID: JIAS

Journal of the International AIDS Society

EISSN: 1758-2652
Publisher: Wiley

ARTICLE INFORMATION
==============================
Article ID: 18440-096
Subjects: C45 - Prevention of mother-to-child transmission

TUPDC0205: Social and structural barriers to uptake of PMTCT in Nyanza province, Kenyaa community-based survey

Kohler P. 1 §
Okanda J. 2
Kinuthia J. 3
Olilo G. 2
Odhiambo F. 2
Mills L. 4
Kayla L. 4
John-Stewart G. 5
1 University of Washington, Global Health and Nursing, Seattle, United States
2 Kenya Medical Research Institute (KEMRI)/ Centers for Disease Control and Prevention (CDC) Research and Public Health Collaboration, Kisumu, Kenya
3 University of Nairobi, Kenyatta National Hospital, Obstetrics and Gynaecology, Nairobi, Kenya
4 Center for Disease Control and Prevention, Kisumu, Kenya
5 University of Washington, Global Health, Medicine, Epidemiology and Pediatrics, Seattle, United States
§ Presenting author email: pkohler2@uw.edu
Electronic publication date: 2012 Oct 22
Publication date: 2012
Volume: 15
Issue: Suppl 3
Electronic Location ID: 18440-096
Copyright: © 2012 P. Kohler et al.
Copyright year: 2012
License: This is an open-access article distributed under the terms of the Creative Commons Attribution License, which permits unrestricted use, distribution, and reproduction in any medium, provided the original work is properly cited.
License URL: http://creativecommons.org/licenses/by/2.0/

JOURNAL INFORMATION
==============================
NLM Title Abbreviation: J Int AIDS Soc
Journal ID: J Int AIDS Soc
NLM Journal ID: 101478566
Journal ID: JIAS

Journal of the International AIDS Society

EISSN: 1758-2652
Publisher: Wiley

ARTICLE INFORMATION
==============================
Article ID: 18440-097
Subjects: C45 - Prevention of mother-to-child transmission

TUPDC0206: Couple counseling increases antiretroviral (ARV) uptake by pregnant women: a case report in Zambia Defense Force health facilities

Kanjipite W. 1
Nikisi J. 2
Chilila M. 3 §
Shasulwe H. 3
Banda J. 3
Mulilo J. 4
1 Jhpiego, Monitoring and Evaluation, Lusaka, Zambia
2 Jhpiego, Technical Department, Lusaka, Zambia
3 Jhpiego, HIV/AIDS, Lusaka, Zambia
4 Zambia Defence Forces, Zambia National Service, Lusaka, Zambia
§ Presenting author email: mchilila@jhpiego.net
Electronic publication date: 2012 Oct 22
Publication date: 2012
Volume: 15
Issue: Suppl 3
Electronic Location ID: 18440-097
Copyright: © 2012 M. Chilila et al.
Copyright year: 2012
License: This is an open-access article distributed under the terms of the Creative Commons Attribution License, which permits unrestricted use, distribution, and reproduction in any medium, provided the original work is properly cited.
License URL: http://creativecommons.org/licenses/by/2.0/

JOURNAL INFORMATION
==============================
NLM Title Abbreviation: J Int AIDS Soc
Journal ID: J Int AIDS Soc
NLM Journal ID: 101478566
Journal ID: JIAS

Journal of the International AIDS Society

EISSN: 1758-2652
Publisher: Wiley

ARTICLE INFORMATION
==============================
Article ID: 18440-098
Subjects: C46 - Male and female condoms and other physical barriers

WEPDC0201: Efficacy of a randomized intervention trial promoting female condom use among female South African tertiary students

Smit J.A. 1 §
Hoffman S. 2
Mabude Z. 1
Beksinska M. 1
Stein Z.A. 2 3
Ngoloyi C. 1
Kelvin E.A. 2 4
Masvawure T.B. 2
Pienaar J. 1
Leu C.-S. 2 5
Exner T. 2
Mantell J.E. 2
1 MatCH [Maternal, Adolescent and Child Health], Durban, South Africa
2 HIV Center for Clinical and Behavioral Studies, New York State Psychiatric Institute and Columbia University, Psychiatry, New York, United States
3 Columbia University, G.H. Sergievsky Center, New York, United States
4 City University of New York School of Public Health at Hunter College, Epidemiology, New York, United States
5 Mailman School of Public Health, Columbia University, Biostatistics, New York, United States
§ Presenting author email: jsmit@match.org.za
Electronic publication date: 2012 Oct 22
Publication date: 2012
Volume: 15
Issue: Suppl 3
Electronic Location ID: 18440-098
Copyright: © 2012 J.A. Smit et al.
Copyright year: 2012
License: This is an open-access article distributed under the terms of the Creative Commons Attribution License, which permits unrestricted use, distribution, and reproduction in any medium, provided the original work is properly cited.
License URL: http://creativecommons.org/licenses/by/2.0/

JOURNAL INFORMATION
==============================
NLM Title Abbreviation: J Int AIDS Soc
Journal ID: J Int AIDS Soc
NLM Journal ID: 101478566
Journal ID: JIAS

Journal of the International AIDS Society

EISSN: 1758-2652
Publisher: Wiley

ARTICLE INFORMATION
==============================
Article ID: 18440-099
Subjects: C47 - Harm reduction, including reduction of unsafe injecting and other approaches

THAC0503: Cost-effectiveness of combined sexual and injection risk reduction interventions among female sex workers who inject drugs in two very distinct Mexican-U.S. border cities

Burgos J.L. 1 2 §
Patterson T.L. 3
Graff-Zivin J.S. 4
Kahn J.G. 5
Rangel M.G. 6
Ojeda V.D. 1
Lozada M.R. 7
Strathdee S.A. 1
1 University of California San Diego School of Medicine, Division of Global Public Health, La Jolla, United States
2 Universidad Autonoma de Baja California, Facultad de Medicina, Tijuana, Mexico
3 University of California-San Diego (UCSD), Psychiatry, La Jolla, United States
4 University of California San Diego, International Relations and Pacific Studies, La Jolla, United States
5 University of California San Francisco School of Medicine, San Francisco, United States
6 El Colegio de la Frontera Norte, Estudios de Poblacion, Tijuana, Mexico
7 ISESALUD, Programa Estatal de Prevencion y Control del VIH e ITS, Tijuana, Mexico
§ Presenting author email: jlburgos@ucsd.edu
Electronic publication date: 2012 Oct 22
Publication date: 2012
Volume: 15
Issue: Suppl 3
Electronic Location ID: 18440-099
Copyright: © 2012 J.L. Burgos et al.
Copyright year: 2012
License: This is an open-access article distributed under the terms of the Creative Commons Attribution License, which permits unrestricted use, distribution, and reproduction in any medium, provided the original work is properly cited.
License URL: http://creativecommons.org/licenses/by/2.0/

JOURNAL INFORMATION
==============================
NLM Title Abbreviation: J Int AIDS Soc
Journal ID: J Int AIDS Soc
NLM Journal ID: 101478566
Journal ID: JIAS

Journal of the International AIDS Society

EISSN: 1758-2652
Publisher: Wiley

ARTICLE INFORMATION
==============================
Article ID: 18440-100
Subjects: C49 - Male circumcision

TUAC0401: What women think about male circumcisionperceptions of female partners of recently circumcised men in Nyanza province, Kenya

Okeyo Adipo T. 1 §
Westercamp N. 2
Agot K. 3
Jaoko W. 4
Bailey R. 2
1 Nyanza Reproductive Health Society, Kisumu, Kenya
2 University of Illinois at Chicago, School of Public Health, Chicago, United States
3 Impact Research and Development Organisation, Kisumu, Kenya
4 University of Nairobi, Nairobi, Kenya
§ Presenting author email: timadipo@yahoo.com
Electronic publication date: 2012 Oct 22
Publication date: 2012
Volume: 15
Issue: Suppl 3
Electronic Location ID: 18440-100
Copyright: © 2012 T. Okeyo Adipo et al.
Copyright year: 2012
License: This is an open-access article distributed under the terms of the Creative Commons Attribution License, which permits unrestricted use, distribution, and reproduction in any medium, provided the original work is properly cited.
License URL: http://creativecommons.org/licenses/by/2.0/

JOURNAL INFORMATION
==============================
NLM Title Abbreviation: J Int AIDS Soc
Journal ID: J Int AIDS Soc
NLM Journal ID: 101478566
Journal ID: JIAS

Journal of the International AIDS Society

EISSN: 1758-2652
Publisher: Wiley

ARTICLE INFORMATION
==============================
Article ID: 18440-101
Subjects: C49 - Male circumcision

TUAC0402: The efficacy of medical male circumcision against HIV acquisition at 66 months post-procedure in Kisumu, Kenya

Mehta S. 1
Li H. 1
Moses S. 2
Agot K. 3
Maclean I. 2
Hedeker D. 1
Bailey R. 1 §
1 University of Illinois at Chicago School of Public Health, Epidemiology & Biostatistics, Chicago, United States
2 University of Manitoba, Winnipeg, Canada
3 UNIM Project, Kisumu, Kenya
§ Presenting author email: rcbailey@uic.edu
Electronic publication date: 2012 Oct 22
Publication date: 2012
Volume: 15
Issue: Suppl 3
Electronic Location ID: 18440-101
Copyright: © 2012 R. Bailey et al.
Copyright year: 2012
License: This is an open-access article distributed under the terms of the Creative Commons Attribution License, which permits unrestricted use, distribution, and reproduction in any medium, provided the original work is properly cited.
License URL: http://creativecommons.org/licenses/by/2.0/

JOURNAL INFORMATION
==============================
NLM Title Abbreviation: J Int AIDS Soc
Journal ID: J Int AIDS Soc
NLM Journal ID: 101478566
Journal ID: JIAS

Journal of the International AIDS Society

EISSN: 1758-2652
Publisher: Wiley

ARTICLE INFORMATION
==============================
Article ID: 18440-102
Subjects: C49 - Male circumcision

TUAC0403: Decrease of HIV prevalence over time among the male population of Orange Farm, South Africa, following a circumcision roll-out (ANRS-12126)

Auvert B. 1 §
Taljaard D. 2
Sitta R. 3
Reach D. 2
Lissouba P. 3
Bouscaillou J. 3
Singh B. 4
Shabangu D. 2
Nhlapo C. 5
Otchere- Darko J. 2
Mashigo T. 2
Taljaard R. 6
Phatedi G. 2
Tsepe M. 2
Chakela M. 2
Mkhwanazi A. 2
Ntshangase P. 2
Peytavin G. 7
Billy S. 5
Puren A. 4
Lewis D. 4
1 University of Versailles, Versailles, France
2 CHAPS, Johannesburg, South Africa
3 Inserm U1018-CESP, Villejuif, France
4 NICD-NHLS, Johannesburg, South Africa
5 SFH, Johannesburg, South Africa
6 Progressus, Johannesburg, South Africa
7 Hopital Bichat-Claude-Bernard, Paris, France
§ Presenting author email: bertran.auvert@uvsq.fr
Electronic publication date: 2012 Oct 22
Publication date: 2012
Volume: 15
Issue: Suppl 3
Electronic Location ID: 18440-102
Copyright: © 2012 B. Auvert et al.
Copyright year: 2012
License: This is an open-access article distributed under the terms of the Creative Commons Attribution License, which permits unrestricted use, distribution, and reproduction in any medium, provided the original work is properly cited.
License URL: http://creativecommons.org/licenses/by/2.0/

JOURNAL INFORMATION
==============================
NLM Title Abbreviation: J Int AIDS Soc
Journal ID: J Int AIDS Soc
NLM Journal ID: 101478566
Journal ID: JIAS

Journal of the International AIDS Society

EISSN: 1758-2652
Publisher: Wiley

ARTICLE INFORMATION
==============================
Article ID: 18440-103
Subjects: C49 - Male circumcision

TUAC0404: Randomized controlled trial of the Shang Ring versus conventional surgical techniques for adult male circumcision in Kenya and Zambia

Sokal D.C. 1
Awori Q. 2 §
Barone M. 3
Simba R. 4
Bowa K. 5
Zulz R. 5
Cherutich P. 6
Muraguri N. 6
Wekesa J.M. 7
Moguche J. 2
Kasonde P. 8
Lee R. 9
Masson P. 9
Perchal P. 3
Combes S. 10
Hart C. 10
Goldstein M. 2
Li P. 2
1 FHI 360, Clinical Sciences, Durham, United States
2 EngenderHealth, Kisumu, Kenya
3 EngenderHealth, New York, United States
4 Homa Bay District Hosptial, Homa Bay, Kenya
5 University Teaching Hospital, Lusaka, Zambia
6 National AIDS and STD Control Program, Nairobi, Kenya
7 Ministry of Medical Services, Nairobi, Kenya
8 FHI 360, Lusaka, Zambia
9 Weill Cornell Medical College, New York City, United States
10 FHI 360, Durham, United States
§ Presenting author email: qawori@engenderhealth.org
Electronic publication date: 2012 Oct 22
Publication date: 2012
Volume: 15
Issue: Suppl 3
Electronic Location ID: 18440-103
Copyright: © 2012 Q. Awori et al.
Copyright year: 2012
License: This is an open-access article distributed under the terms of the Creative Commons Attribution License, which permits unrestricted use, distribution, and reproduction in any medium, provided the original work is properly cited.
License URL: http://creativecommons.org/licenses/by/2.0/

JOURNAL INFORMATION
==============================
NLM Title Abbreviation: J Int AIDS Soc
Journal ID: J Int AIDS Soc
NLM Journal ID: 101478566
Journal ID: JIAS

Journal of the International AIDS Society

EISSN: 1758-2652
Publisher: Wiley

ARTICLE INFORMATION
==============================
Article ID: 18440-104
Subjects: C49 - Male circumcision

TUAC0405: One arm, open label, prospective, cohort field study to assess the safety and efficacy of the PrePex device for scale-up of non-surgical circumcision when performed by nurses in resource-limited settings for HIV prevention

Vincent M. 1 §
Kaplan S.A. 2
Bitega J.P. 3
Ngeruka M.L. 3
Karema C. 4
Binagwaho A. 4
1 Ministry of Health/TRAC Plus, HIV/AIDS and Sexually transmitted Infections Department, Kigali, Rwanda
2 Weill Cornell Medical College, New York, United States
3 Kanombe Military Hospital, Kigali, Rwanda
4 Ministry of Health, Kigali, Rwanda
§ Presenting author email: mutabazivincent@yahoo.com
Electronic publication date: 2012 Oct 22
Publication date: 2012
Volume: 15
Issue: Suppl 3
Electronic Location ID: 18440-104
Copyright: © 2012 M. Vincent et al.
Copyright year: 2012
License: This is an open-access article distributed under the terms of the Creative Commons Attribution License, which permits unrestricted use, distribution, and reproduction in any medium, provided the original work is properly cited.
License URL: http://creativecommons.org/licenses/by/2.0/

JOURNAL INFORMATION
==============================
NLM Title Abbreviation: J Int AIDS Soc
Journal ID: J Int AIDS Soc
NLM Journal ID: 101478566
Journal ID: JIAS

Journal of the International AIDS Society

EISSN: 1758-2652
Publisher: Wiley

ARTICLE INFORMATION
==============================
Article ID: 18440-105
Subjects: C49 - Male circumcision

MOPDE0104: Service delivery trends in Kenya's voluntary medical male circumcision scale-up from 2008–2011

Mwandi Z. 1 §
Ochieng A. 2
Grund J. 3
Mwalili S. 1
Kimanga D. 2
Otieno G. 4
Ohaga S. 5
Oyaro P. 6
Mwangi C. 7
Knight N. 1
Chesang K. 1
Bock N. 3
1 CDC Kenya, Nairobi, Kenya
2 National AIDS and STD Control Program, Ministry of Health, Nairobi, Kenya
3 Centers for Disease Control and Prevention, Atlanta, United States
4 Nyanza Reproductive Health Society, Kisumu, Kenya
5 Impact Research and Development Organisation, Kisumu, Kenya
6 Kenya Medical Research Institute, FACES Program, Nairobi, Kenya
7 Eastern Deanery AIDS Relief Program, Kisumu, Kenya
§ Presenting author email: zmwandi@ke.cdc.gov
Electronic publication date: 2012 Oct 22
Publication date: 2012
Volume: 15
Issue: Suppl 3
Electronic Location ID: 18440-105
Copyright: © 2012 Z. Mwandi et al.
Copyright year: 2012
License: This is an open-access article distributed under the terms of the Creative Commons Attribution License, which permits unrestricted use, distribution, and reproduction in any medium, provided the original work is properly cited.
License URL: http://creativecommons.org/licenses/by/2.0/

JOURNAL INFORMATION
==============================
NLM Title Abbreviation: J Int AIDS Soc
Journal ID: J Int AIDS Soc
NLM Journal ID: 101478566
Journal ID: JIAS

Journal of the International AIDS Society

EISSN: 1758-2652
Publisher: Wiley

ARTICLE INFORMATION
==============================
Article ID: 18440-106
Subjects: C49 - Male circumcision

MOPDE0105: Male circumcision in Swaziland: demographics, behaviours and HIV prevalence

Reed J.B. 1 §
Mirira M. 2
Grund J. 1
Nqeketo A. 3
Ginindza H. 3
Donnell D. 4
Nkambule R. 3
Bicego G. 5
Ryan C. 6
Justman J. 7
1 U.S. Centers for Disease Control and Prevention, Center for Global Health, Division of HIV/AIDS, Atlanta, United States
2 USAID Swaziland, Mbabane, Swaziland
3 Ministry of Health - Swaziland, Mbabane, Swaziland
4 Fred Hutchinson Cancer Research Center, Seattle, United States
5 U.S. Centers for Disease Control and Prevention Swaziland, Mbabane, Swaziland
6 Office of the Global AIDS Coordinator, Washington, United States
7 ICAP at the Mailman School of Public Health at Columbia University, New York, United States
§ Presenting author email: eso7@cdc.gov
Electronic publication date: 2012 Oct 22
Publication date: 2012
Volume: 15
Issue: Suppl 3
Electronic Location ID: 18440-106
Copyright: © 2012 J.B. Reed et al.
Copyright year: 2012
License: This is an open-access article distributed under the terms of the Creative Commons Attribution License, which permits unrestricted use, distribution, and reproduction in any medium, provided the original work is properly cited.
License URL: http://creativecommons.org/licenses/by/2.0/

JOURNAL INFORMATION
==============================
NLM Title Abbreviation: J Int AIDS Soc
Journal ID: J Int AIDS Soc
NLM Journal ID: 101478566
Journal ID: JIAS

Journal of the International AIDS Society

EISSN: 1758-2652
Publisher: Wiley

ARTICLE INFORMATION
==============================
Article ID: 18440-107
Subjects: C50 - Sexually transmitted infection (STI) prevention and control

TUAC0203: Increased routine screening for syphilis and falling syphilis incidence in HIV positive and HIV negative men who have sex with men: implications for syphilis and HIV prevention

Stoové M. 1 2 §
El-Hayek C. 1
Fairley C. 3
Goller J. 1
Leslie D. 4
Roth N. 5
Tee B. 6
Denham I. 3
Chen M. 3
Hellard M. 1 2 7
1 Centre for Population Health, Burnet Institute, Melbourne, Australia
2 Epidemiology and Preventive Medicine, Monash University, Melbourne, Australia
3 Melbourne Sexual Health Centre, Melbourne, Australia
4 Victorian Infectious Diseases Reference Laboratory, Melbourne, Australia
5 Prahran Market Clinic, Melbourne, Australia
6 The Centre Clinic, Victorian AIDS Council, Melbourne, Australia
7 Nossal Institute, University of Melbourne, Melbourne, Australia
§ Presenting author email: stoove@burnet.edu.au
Electronic publication date: 2012 Oct 22
Publication date: 2012
Volume: 15
Issue: Suppl 3
Electronic Location ID: 18440-107
Copyright: © 2012 M. Stoové et al.
Copyright year: 2012
License: This is an open-access article distributed under the terms of the Creative Commons Attribution License, which permits unrestricted use, distribution, and reproduction in any medium, provided the original work is properly cited.
License URL: http://creativecommons.org/licenses/by/2.0/

JOURNAL INFORMATION
==============================
NLM Title Abbreviation: J Int AIDS Soc
Journal ID: J Int AIDS Soc
NLM Journal ID: 101478566
Journal ID: JIAS

Journal of the International AIDS Society

EISSN: 1758-2652
Publisher: Wiley

ARTICLE INFORMATION
==============================
Article ID: 18440-108
Subjects: C50 - Sexually transmitted infection (STI) prevention and control

TUPDC0102: Partner notification outcomes for MSM and heterosexuals with STI/HIV: challenges at different stages

van Aar F. 1
van Weert Y. 1
Spijker R. 2
Götz H. 3
Op de Coul E. 1 §
1 National Institute for Public Health and the Environment, Center for Infectious Diseases Control, Bilthoven, Netherlands
2 STI AIDS Netherlands, Amsterdam, Netherlands
3 Rotterdam Rijnmond Public Health Service, Rotterdam, Netherlands
§ Presenting author email: eline.op.de.coul@rivm.nl
Electronic publication date: 2012 Oct 22
Publication date: 2012
Volume: 15
Issue: Suppl 3
Electronic Location ID: 18440-108
Copyright: © 2012 E. Op de Coul et al.
Copyright year: 2012
License: This is an open-access article distributed under the terms of the Creative Commons Attribution License, which permits unrestricted use, distribution, and reproduction in any medium, provided the original work is properly cited.
License URL: http://creativecommons.org/licenses/by/2.0/

JOURNAL INFORMATION
==============================
NLM Title Abbreviation: J Int AIDS Soc
Journal ID: J Int AIDS Soc
NLM Journal ID: 101478566
Journal ID: JIAS

Journal of the International AIDS Society

EISSN: 1758-2652
Publisher: Wiley

ARTICLE INFORMATION
==============================
Article ID: 18440-109
Subjects: C50 - Sexually transmitted infection (STI) prevention and control

TUPDC0106: Sexually transmitted disease diagnoses associated with exchange sex among Hispanic migrant men who have sex with men in the United States

Valverde E. 1
Dinenno E. 1 §
Oster A. 1
Chavez P. 1
Thomas P. 1
Schulden J. 2
Heffelfinger J. 1
1 Centers for Disease Control and Prevention, Division of HIV/AIDS, Atlanta, United States
2 National Institute on Drug Abuse (NIDA) National Institutes of Health (NIH), Bethesda, United States
§ Presenting author email: eid8@cdc.gov
Electronic publication date: 2012 Oct 22
Publication date: 2012
Volume: 15
Issue: Suppl 3
Electronic Location ID: 18440-109
Copyright: © 2012 E. Dinenno et al.
Copyright year: 2012
License: This is an open-access article distributed under the terms of the Creative Commons Attribution License, which permits unrestricted use, distribution, and reproduction in any medium, provided the original work is properly cited.
License URL: http://creativecommons.org/licenses/by/2.0/

JOURNAL INFORMATION
==============================
NLM Title Abbreviation: J Int AIDS Soc
Journal ID: J Int AIDS Soc
NLM Journal ID: 101478566
Journal ID: JIAS

Journal of the International AIDS Society

EISSN: 1758-2652
Publisher: Wiley

ARTICLE INFORMATION
==============================
Article ID: 18440-110
Subjects: C51 - Pre-exposure and post-exposure prophylaxis

TUAC0102: PrEP has high efficacy for HIV-1 prevention among higher-risk HIV-1 serodiscordant couples: a subgroup analysis from the Partners PrEP Study

Kahle E. 1 §
Donnell D. 2
James H. 3
Thomas K. 3
John-Stewart G. 3
Nakku-Joloba E. 4
Bukusi E. 5
Lingappa J. 3
Celum C. 3
Baeten J. 3
Partners PrEP Study
1 University of Washington, Epidemiology, Seattle, United States
2 Fred Hutchinson Cancer Research Center, Seattle, United States
3 University of Washington, Seattle, United States
4 Makerere University, Kampala, Uganda
5 Kenya Medical Research Institute, Nairobi, Kenya
§ Presenting author email: ekahle@u.washington.edu
Electronic publication date: 2012 Oct 22
Publication date: 2012
Volume: 15
Issue: Suppl 3
Electronic Location ID: 18440-110
Copyright: © 2012 E. Kahle et al.
Copyright year: 2012
License: This is an open-access article distributed under the terms of the Creative Commons Attribution License, which permits unrestricted use, distribution, and reproduction in any medium, provided the original work is properly cited.
License URL: http://creativecommons.org/licenses/by/2.0/

JOURNAL INFORMATION
==============================
NLM Title Abbreviation: J Int AIDS Soc
Journal ID: J Int AIDS Soc
NLM Journal ID: 101478566
Journal ID: JIAS

Journal of the International AIDS Society

EISSN: 1758-2652
Publisher: Wiley

ARTICLE INFORMATION
==============================
Article ID: 18440-111
Subjects: C51 - Pre-exposure and post-exposure prophylaxis

TUAC0302: Anticipated risk compensation with pre-exposure prophylaxis use among North American men who have sex with men using an internet social network

Krakower D. 1 §
Mimiaga M. 2
Rosenberger J. 3
Novak D. 4
Mitty J. 5 6
White J. 6
Mayer K. 5 6
1 Beth Israel Deaconess Medical Center, Division of Infectious Diseases, Boston, United States
2 Fenway Health/Harvard School of Public Health/Harvard Medical School, Boston, United States
3 George Mason University, Fairfax, United States
4 Online Buddies, Inc., Cambridge, United States
5 Beth Israel Deaconess Medical Center/ Harvard Medical School, Boston, United States
6 Fenway Health, Boston, United States
§ Presenting author email: dkakowe@bidmc.harvard.edu
Electronic publication date: 2012 Oct 22
Publication date: 2012
Volume: 15
Issue: Suppl 3
Electronic Location ID: 18440-111
Copyright: © 2012 D. Krakower et al.
Copyright year: 2012
License: This is an open-access article distributed under the terms of the Creative Commons Attribution License, which permits unrestricted use, distribution, and reproduction in any medium, provided the original work is properly cited.
License URL: http://creativecommons.org/licenses/by/2.0/

JOURNAL INFORMATION
==============================
NLM Title Abbreviation: J Int AIDS Soc
Journal ID: J Int AIDS Soc
NLM Journal ID: 101478566
Journal ID: JIAS

Journal of the International AIDS Society

EISSN: 1758-2652
Publisher: Wiley

ARTICLE INFORMATION
==============================
Article ID: 18440-112
Subjects: C51 - Pre-exposure and post-exposure prophylaxis

TUAC0303: Acceptability of HIV pre-exposure prophylaxis (PrEP) with Truvada among men who have sex with men (MSM) and male-to-female transgender persons (TG) in northern Thailand

Yang D. 1 2 §
Chariyalertsak C. 3
Wongthanee A. 2
Kawichai S. 1 2
Yotruean K. 3
Guadamuz T. 4 5
Suwanvanichkij V. 1
Beyrer C. 1
Chariyalertsak S. 2
1 Johns Hopkins Bloomberg School of Public Health, Baltimore, United States
2 Research Institute for Health Sciences, Chiang Mai University, Chiang Mai, Thailand
3 Chiang Mai Provincial Health Office, Thailand MOPH, Chiang Mai, Thailand
4 Center for Health Policy Studies, Mahidol University, Bangkok, Thailand
5 University of Pittsburgh Graduate School of Public Health, Pittsburgh, United States
§ Presenting author email: dyang20@uic.edu
Electronic publication date: 2012 Oct 22
Publication date: 2012
Volume: 15
Issue: Suppl 3
Electronic Location ID: 18440-112
Copyright: © 2012 D. Yang et al.
Copyright year: 2012
License: This is an open-access article distributed under the terms of the Creative Commons Attribution License, which permits unrestricted use, distribution, and reproduction in any medium, provided the original work is properly cited.
License URL: http://creativecommons.org/licenses/by/2.0/

JOURNAL INFORMATION
==============================
NLM Title Abbreviation: J Int AIDS Soc
Journal ID: J Int AIDS Soc
NLM Journal ID: 101478566
Journal ID: JIAS

Journal of the International AIDS Society

EISSN: 1758-2652
Publisher: Wiley

ARTICLE INFORMATION
==============================
Article ID: 18440-113
Subjects: C51 - Pre-exposure and post-exposure prophylaxis

FRLBC03: Method of hormonal contraception is associated with lower tenofovir concentration in healthy women (MTN-001): implications for pre-exposure prophylaxis

Coleman J. 1 §
Chaturvedula A. 2
Hendrix C. 3
MTN-001 Protocol Team
1 Johns Hopkins University School of Medicine, Gynecology & Obstetrics, Baltimore, United States
2 College of Pharmacy and Health Sciences at Mercer University, Atlanta, United States
3 Johns Hopkins University School of Medicine, Medicine, Baltimore, United States
§ Presenting author . Email: jcolem38@jhmi.edu
Electronic publication date: 2012 Oct 22
Publication date: 2012
Volume: 15
Issue: Suppl 3
Electronic Location ID: 18440-113
Copyright: © 2012 J. Coleman et al.
Copyright year: 2012
License: This is an open-access article distributed under the terms of the Creative Commons Attribution License, which permits unrestricted use, distribution, and reproduction in any medium, provided the original work is properly cited.
License URL: http://creativecommons.org/licenses/by/2.0/

JOURNAL INFORMATION
==============================
NLM Title Abbreviation: J Int AIDS Soc
Journal ID: J Int AIDS Soc
NLM Journal ID: 101478566
Journal ID: JIAS

Journal of the International AIDS Society

EISSN: 1758-2652
Publisher: Wiley

ARTICLE INFORMATION
==============================
Article ID: 18440-114
Subjects: C51 - Pre-exposure and post-exposure prophylaxis

FRLBX04: Pre-exposure prophylaxis (PrEP) will have a limited impact on the prevalence of HIV-1 drug resistance in sub-Saharan Africa: comparison of mathematical models

van de Vijver D. 1
Nichols B. 1 §
Abbas U. 2
Cambiano V. 3
Eaton J. 4
Glaubius R. 5
Lythgoe K. 4
Mellors J. 6
Phillips A. 3
Sigaloff K. 7 8
Hallett T. 4
1 Erasmus Medical Centre, Virology, Rotterdam, Netherlands
2 Cleveland Clinic Foundation, Infectious Diseases, Cleveland, United States
3 Infection and Population Health, University College London, London, United Kingdom
4 Infectious Disease Epidemiology, Imperial College, London, United Kingdom
5 Cleveland Clinic Foundation, Infection and Population Health, Cleveland, United States
6 Infectious Diseases, University of Pittsburgh School of Medicine, Pittsburgh, United States
7 PharmAccess Foundation, Amsterdam, Netherlands
8 Amsterdam Institute for Global Health and Development (AIGHD), Amsterdam, Netherlands
§ Presenting author email: b.nichols@erasmusmc.nl
Electronic publication date: 2012 Oct 22
Publication date: 2012
Volume: 15
Issue: Suppl 3
Electronic Location ID: 18440-114
Copyright: © 2012 B. Nichols et al.
Copyright year: 2012
License: This is an open-access article distributed under the terms of the Creative Commons Attribution License, which permits unrestricted use, distribution, and reproduction in any medium, provided the original work is properly cited.
License URL: http://creativecommons.org/licenses/by/2.0/

JOURNAL INFORMATION
==============================
NLM Title Abbreviation: J Int AIDS Soc
Journal ID: J Int AIDS Soc
NLM Journal ID: 101478566
Journal ID: JIAS

Journal of the International AIDS Society

EISSN: 1758-2652
Publisher: Wiley

ARTICLE INFORMATION
==============================
Article ID: 18440-115
Subjects: C51 - Pre-exposure and post-exposure prophylaxis

TUPDC0303: Acceptability of oral intermittent pre-exposure prophylaxis as a biomedical HIV prevention strategyresults from the South African ADAPT (HPTN 067) Preparatory Study

Mark D. 1
Amico K.R. 2 3
Wallace M. 1
Roux S. 1
Grant R. 4 5
Wood R. 1
Bekker L.-G. 1
1 Desmond Tutu HIV Centre, Institute of Infectious Disease and Molecular Medicine and Department of Medicine, University of Cape Town, Cape Town, South Africa
2 Applied Health Research, Connecticut, United States
3 Center for Health Intervention and Prevention, University of Connecticut, Connecticut, United States
4 Gladstone Institute of Virology and Immunology, San Francisco, United States
5 University of California, San Francisco, United States
Electronic publication date: 2012 Oct 22
Publication date: 2012
Volume: 15
Issue: Suppl 3
Electronic Location ID: 18440-115
Copyright: © 2012 et al.
Copyright year: 2012
License: This is an open-access article distributed under the terms of the Creative Commons Attribution License, which permits unrestricted use, distribution, and reproduction in any medium, provided the original work is properly cited.
License URL: http://creativecommons.org/licenses/by/2.0/

JOURNAL INFORMATION
==============================
NLM Title Abbreviation: J Int AIDS Soc
Journal ID: J Int AIDS Soc
NLM Journal ID: 101478566
Journal ID: JIAS

Journal of the International AIDS Society

EISSN: 1758-2652
Publisher: Wiley

ARTICLE INFORMATION
==============================
Article ID: 18440-116
Subjects: C51 - Pre-exposure and post-exposure prophylaxis

TUPDC0305: Experience of a NYC hospital with non-occupational post-exposure prophylaxis (nPEP)

Urbina A. 1 §
Osorio G. 1
Daniel E. 2 3
Galatowitsch P. 4
Riggan B. 5
Hennessey Z. 5
Sharp V. 1
1 Center for Comprehensive Care, St Luke's Roosevelt Hospital, Medicine, New York, United States
2 Center for Comprehensive Care, St Luke's Roosevelt Hospital, Emergency Medicine, New York, United States
3 Emergency Medicine, Columbia University College of Physicians and Surgeons, New York, United States
4 HealthClear Strategies, New York, United States
5 Center for Comprehensive Care, St Luke's Roosevelt Hospital, New York, United States
§ Presenting author email: aurbina@chpnet.org
Electronic publication date: 2012 Oct 22
Publication date: 2012
Volume: 15
Issue: Suppl 3
Electronic Location ID: 18440-116
Copyright: © 2012 A. Urbina et al.
Copyright year: 2012
License: This is an open-access article distributed under the terms of the Creative Commons Attribution License, which permits unrestricted use, distribution, and reproduction in any medium, provided the original work is properly cited.
License URL: http://creativecommons.org/licenses/by/2.0/

JOURNAL INFORMATION
==============================
NLM Title Abbreviation: J Int AIDS Soc
Journal ID: J Int AIDS Soc
NLM Journal ID: 101478566
Journal ID: JIAS

Journal of the International AIDS Society

EISSN: 1758-2652
Publisher: Wiley

ARTICLE INFORMATION
==============================
Article ID: 18440-117
Subjects: C51 - Pre-exposure and post-exposure prophylaxis

TUPDC0306: Project PrEP Talkan in-depth qualitative analysis of PrEP acceptability, expectations and risk compensation beliefs among United States MSM

Underhill K. 1 §
Morrow K. 2
Operario D. 3
Ducharme R. 4
Kuo C. 3
Mayer K. 5
1 Yale University, New Haven, United States
2 Department of Psychiatry & Human Behavior, Brown University-The Miriam Hospital, Providence, United States
3 Department of Community Health, Brown University, Providence, United States
4 The Miriam Hospital, Providence, United States
5 The Fenway Institute, Boston, United States
§ Presenting author email: kristen.underhill@yale.edu
Electronic publication date: 2012 Oct 22
Publication date: 2012
Volume: 15
Issue: Suppl 3
Electronic Location ID: 18440-117
Copyright: © 2012 K. Underhill et al.
Copyright year: 2012
License: This is an open-access article distributed under the terms of the Creative Commons Attribution License, which permits unrestricted use, distribution, and reproduction in any medium, provided the original work is properly cited.
License URL: http://creativecommons.org/licenses/by/2.0/

JOURNAL INFORMATION
==============================
NLM Title Abbreviation: J Int AIDS Soc
Journal ID: J Int AIDS Soc
NLM Journal ID: 101478566
Journal ID: JIAS

Journal of the International AIDS Society

EISSN: 1758-2652
Publisher: Wiley

ARTICLE INFORMATION
==============================
Article ID: 18440-118
Subjects: C52 - Microbicides

WEPDC0101: Sustained release tenofovir and maraviroc from intravaginal ring in sheep

Smith T.J. 1 §
1 Auritec Pharmaceuticals, Santa Monica, United States
§ Presenting author email: tsmith@auritec.net
Electronic publication date: 2012 Oct 22
Publication date: 2012
Volume: 15
Issue: Suppl 3
Electronic Location ID: 18440-118
Copyright: © 2012 T.J. Smith et al.
Copyright year: 2012
License: This is an open-access article distributed under the terms of the Creative Commons Attribution License, which permits unrestricted use, distribution, and reproduction in any medium, provided the original work is properly cited.
License URL: http://creativecommons.org/licenses/by/2.0/

JOURNAL INFORMATION
==============================
NLM Title Abbreviation: J Int AIDS Soc
Journal ID: J Int AIDS Soc
NLM Journal ID: 101478566
Journal ID: JIAS

Journal of the International AIDS Society

EISSN: 1758-2652
Publisher: Wiley

ARTICLE INFORMATION
==============================
Article ID: 18440-119
Subjects: C52 - Microbicides

WEPDC0102: Sustained-release saquinavir from an intravaginal ring in sheep

Willis R.A. 1 §
Malone A.M. 1
Moss J.A. 2
Baum M.M. 2
Vincent K.L. 3
Motamedi M. 3
Kennedy S. 2
Gilman J. 1
Butkyavichene I. 1
Nguyen C. 1
Smith T.J. 1
1 Auritec Pharmaceuticals, Pasadena, United States
2 Oak Crest Institute of Science, Pasadena, United States
3 University of Texas Medical Branch, Galveston, United States
§ Presenting author email: rwillis@auritecpharma.com
Electronic publication date: 2012 Oct 22
Publication date: 2012
Volume: 15
Issue: Suppl 3
Electronic Location ID: 18440-119
Copyright: © 2012 R.A. Willis et al.
Copyright year: 2012
License: This is an open-access article distributed under the terms of the Creative Commons Attribution License, which permits unrestricted use, distribution, and reproduction in any medium, provided the original work is properly cited.
License URL: http://creativecommons.org/licenses/by/2.0/

JOURNAL INFORMATION
==============================
NLM Title Abbreviation: J Int AIDS Soc
Journal ID: J Int AIDS Soc
NLM Journal ID: 101478566
Journal ID: JIAS

Journal of the International AIDS Society

EISSN: 1758-2652
Publisher: Wiley

ARTICLE INFORMATION
==============================
Article ID: 18440-120
Subjects: C52 - Microbicides

WEPDC0104: Sustained release raltegravir from an intravaginal ring in sheep

Malone A. 1 §
Moss J. 2
Willis R. 1
Baum M. 2
Gilman J. 1
Butkyavichene I. 1
Kennedy S. 2
Nguyen C. 1
Smith T. 1 2
1 Auritec Pharmaceuticals, Santa Monica, United States
2 Oak Crest Institute of Science, Pasadena, United States
§ Presenting author email: amalone@auritecpharma.com
Electronic publication date: 2012 Oct 22
Publication date: 2012
Volume: 15
Issue: Suppl 3
Electronic Location ID: 18440-120
Copyright: © 2012 A. Malone et al.
Copyright year: 2012
License: This is an open-access article distributed under the terms of the Creative Commons Attribution License, which permits unrestricted use, distribution, and reproduction in any medium, provided the original work is properly cited.
License URL: http://creativecommons.org/licenses/by/2.0/

JOURNAL INFORMATION
==============================
NLM Title Abbreviation: J Int AIDS Soc
Journal ID: J Int AIDS Soc
NLM Journal ID: 101478566
Journal ID: JIAS

Journal of the International AIDS Society

EISSN: 1758-2652
Publisher: Wiley

ARTICLE INFORMATION
==============================
Article ID: 18440-121
Subjects: C52 - Microbicides

WEPDC0105: High maraviroc concentrations in rectal secretions after oral dosing do not prevent rectal SHIV transmission in macaques

Massud I. 1
Aung W. 1
Martin A. 1
Bachman S. 1
Mitchell J. 1
Deyounks F. 1
Kersh E. 1
Pau C.-P. 1
Heneine W. 1
Garcia-Lerma J.G. 1 §
1 Center for Disease Control and Prevention, Atlanta, United States
§ Presenting author email: jng5@cdc.gov
Electronic publication date: 2012 Oct 22
Publication date: 2012
Volume: 15
Issue: Suppl 3
Electronic Location ID: 18440-121
Copyright: © 2012 J.G. Garcia-Lerma et al.
Copyright year: 2012
License: This is an open-access article distributed under the terms of the Creative Commons Attribution License, which permits unrestricted use, distribution, and reproduction in any medium, provided the original work is properly cited.
License URL: http://creativecommons.org/licenses/by/2.0/

JOURNAL INFORMATION
==============================
NLM Title Abbreviation: J Int AIDS Soc
Journal ID: J Int AIDS Soc
NLM Journal ID: 101478566
Journal ID: JIAS

Journal of the International AIDS Society

EISSN: 1758-2652
Publisher: Wiley

ARTICLE INFORMATION
==============================
Article ID: 18440-122
Subjects: C54 - Antiretroviral treatment of people living with HIV for prevention

TUAC0103: Lack of effectiveness of antiretroviral therapy (ART) as an HIV prevention tool for serodiscordant couples in a rural ART program without viral load monitoring in Uganda

Birungi J. 1 §
Wang H. 2
Ngolobe M.H. 3
Muldoon K. 4
Khanakwa S. 3
King R. 5
Kaleebu P. 6
Shannon K. 4 7
Ochai R. 8
Montaner J. 4 7
Mills E. 9
Laurenco L. 7
Abdallar N. 8
Moore D. 4 7
1 The AIDS Support Organisation (TASO), Research and Medical Capacity Building, Kampala, Uganda
2 University of British Columbia BC, Vancouver, Canada
3 The AIDS Support Organisation (TASO), Jinja, Uganda
4 Division of AIDS, University of British Columbia, Vancouver, Canada
5 University of California at San Francisco, San Francisco, United States
6 Uganda Virus Research Institute, Entebbe, Uganda
7 British Columbia Centre for Excellence in HIV/AIDS, Vancouver, Canada
8 The AIDS Support Organisation (TASO), Kampala, Uganda
9 University of Ottawa, Ottawa, Canada
§ Presenting author email: birungijophine@yahoo.com
Electronic publication date: 2012 Oct 22
Publication date: 2012
Volume: 15
Issue: Suppl 3
Electronic Location ID: 18440-122
Copyright: © 2012 J. Birungi et al.
Copyright year: 2012
License: This is an open-access article distributed under the terms of the Creative Commons Attribution License, which permits unrestricted use, distribution, and reproduction in any medium, provided the original work is properly cited.
License URL: http://creativecommons.org/licenses/by/2.0/

JOURNAL INFORMATION
==============================
NLM Title Abbreviation: J Int AIDS Soc
Journal ID: J Int AIDS Soc
NLM Journal ID: 101478566
Journal ID: JIAS

Journal of the International AIDS Society

EISSN: 1758-2652
Publisher: Wiley

ARTICLE INFORMATION
==============================
Article ID: 18440-123
Subjects: C54 - Antiretroviral treatment of people living with HIV for prevention

MOPDC0104: Treatment as prevention in a country with high ART coveragethe Namibia example

Kamwi R. 1 §
Hamunime N. 1
Odiit M. 2
Gweshe J. 1
Muadinohamba A. 1
Shihepo E. 1
Van Renterghem H. 2
Jonas A. 1
De Klerk M. 1
Mahy M. 3
Forster N. 1
Kahuure K. 1
1 Ministry of Health and Social Services (MOHSS), Windhoek, Namibia
2 UNAIDS, Windhoek, Namibia
3 UNAIDS, Geneva, Switzerland
§ Presenting author email: rkamwi@mhss.gov.na
Electronic publication date: 2012 Oct 22
Publication date: 2012
Volume: 15
Issue: Suppl 3
Electronic Location ID: 18440-123
Copyright: © 2012 R. Kamwi et al.
Copyright year: 2012
License: This is an open-access article distributed under the terms of the Creative Commons Attribution License, which permits unrestricted use, distribution, and reproduction in any medium, provided the original work is properly cited.
License URL: http://creativecommons.org/licenses/by/2.0/

JOURNAL INFORMATION
==============================
NLM Title Abbreviation: J Int AIDS Soc
Journal ID: J Int AIDS Soc
NLM Journal ID: 101478566
Journal ID: JIAS

Journal of the International AIDS Society

EISSN: 1758-2652
Publisher: Wiley

ARTICLE INFORMATION
==============================
Article ID: 18440-124
Subjects: C54 - Antiretroviral treatment of people living with HIV for prevention

MOPDC0105: Antiretroviral therapy for prevention of HIV transmission in HIV-discordant couples: a systematic review of the observational literature

Anglemyer A. 1 §
Rutherford G. 1
Baggaley R. 2
Egger M. 3
Siegfried N. 4
1 University of California San Francisco, Global Health Sciences, San Francisco, United States
2 HIV/AIDS Department, World Health Organization, Geneva, Switzerland
3 University of Bern, Institut für Sozial- und Präventivmedizin, Bern, Switzerland
4 Department of Public Health and Primary Care, University of Cape Town, Cape Town, South Africa
§ Presenting author email: grutherford@psg.ucsf.edu
Electronic publication date: 2012 Oct 22
Publication date: 2012
Volume: 15
Issue: Suppl 3
Electronic Location ID: 18440-124
Copyright: © 2012 A. Anglemyer et al.
Copyright year: 2012
License: This is an open-access article distributed under the terms of the Creative Commons Attribution License, which permits unrestricted use, distribution, and reproduction in any medium, provided the original work is properly cited.
License URL: http://creativecommons.org/licenses/by/2.0/

JOURNAL INFORMATION
==============================
NLM Title Abbreviation: J Int AIDS Soc
Journal ID: J Int AIDS Soc
NLM Journal ID: 101478566
Journal ID: JIAS

Journal of the International AIDS Society

EISSN: 1758-2652
Publisher: Wiley

ARTICLE INFORMATION
==============================
Article ID: 18440-125
Subjects: C57 - Approaches to improving adherence to prevention interventions

MOPDC0306: Using surveillance data to identify HIV-positive persons out-of-care (OOC) in New York City (NYC) and offer linkage to care and HIV partner services

Udeagu C.-C. 1 §
Webster-León T. 1
Bocour A. 1
Michel P. 1
Shepard C. 1
1 New York City Department of Health and Mental Hygiene, Bureau of HIV/AIDS Prevention and Control, Queens, United States
§ Presenting author email: cudeagu@health.nyc.gov
Electronic publication date: 2012 Oct 22
Publication date: 2012
Volume: 15
Issue: Suppl 3
Electronic Location ID: 18440-125
Copyright: © 2012 C.-C. Udeagu et al.
Copyright year: 2012
License: This is an open-access article distributed under the terms of the Creative Commons Attribution License, which permits unrestricted use, distribution, and reproduction in any medium, provided the original work is properly cited.
License URL: http://creativecommons.org/licenses/by/2.0/

JOURNAL INFORMATION
==============================
NLM Title Abbreviation: J Int AIDS Soc
Journal ID: J Int AIDS Soc
NLM Journal ID: 101478566
Journal ID: JIAS

Journal of the International AIDS Society

EISSN: 1758-2652
Publisher: Wiley

ARTICLE INFORMATION
==============================
Article ID: 18440-126
Subjects: C58 - Combination of prevention and treatment of HIV

TUAC0301: HIV-negative and HIV-positive gay men's attitudes towards antiretroviral-based preventionsimilar attitudes to pre-exposure prophylaxis (PrEP) but greater scepticism among HIV-negative men about ‘treatment as prevention’

Holt M. 1 §
Murphy D. 1 2
Callander D. 1
Ellard J. 1
Rosengarten M. 3
Kippax S. 4
de Wit J.B.F. 1 5
1 The University of New South Wales, National Centre in HIV Social Research, Sydney, Australia
2 Australian Federation of AIDS Organisations, Sydney, Australia
3 Goldsmiths, Department of Sociology, London, United Kingdom
4 The University of New South Wales, Social Policy Research Centre, Sydney, Australia
5 Department of Social and Organizational Psychology, Utrecht University, Utrecht, Netherlands
§ Presenting author email: m.holt@unsw.edu.au
Electronic publication date: 2012 Oct 22
Publication date: 2012
Volume: 15
Issue: Suppl 3
Electronic Location ID: 18440-126
Copyright: © 2012 M. Holt et al.
Copyright year: 2012
License: This is an open-access article distributed under the terms of the Creative Commons Attribution License, which permits unrestricted use, distribution, and reproduction in any medium, provided the original work is properly cited.
License URL: http://creativecommons.org/licenses/by/2.0/

JOURNAL INFORMATION
==============================
NLM Title Abbreviation: J Int AIDS Soc
Journal ID: J Int AIDS Soc
NLM Journal ID: 101478566
Journal ID: JIAS

Journal of the International AIDS Society

EISSN: 1758-2652
Publisher: Wiley

ARTICLE INFORMATION
==============================
Article ID: 18440-127
Subjects: C59 - HIV counselling, testing and diagnostic strategies

TUAC0105: The critical role of social cohesion on uptake of HIV testing and ART in Zambia

Gari S. 1 §
Musheke M. 1
Ntalasha H. 2
Malungo J.R.S. 2
Merten S. 1
1 Department of Epidemiology and Public Health, Swiss Tropical and Public Health Institute, Basel, Switzerland
2 Department of Humanities and Social Sciences, University of Zambia, Lusaka, Zambia
§ Presenting author email: sara.gari@unibas.ch
Electronic publication date: 2012 Oct 22
Publication date: 2012
Volume: 15
Issue: Suppl 3
Electronic Location ID: 18440-127
Copyright: © 2012 S. Gari et al.
Copyright year: 2012
License: This is an open-access article distributed under the terms of the Creative Commons Attribution License, which permits unrestricted use, distribution, and reproduction in any medium, provided the original work is properly cited.
License URL: http://creativecommons.org/licenses/by/2.0/

JOURNAL INFORMATION
==============================
NLM Title Abbreviation: J Int AIDS Soc
Journal ID: J Int AIDS Soc
NLM Journal ID: 101478566
Journal ID: JIAS

Journal of the International AIDS Society

EISSN: 1758-2652
Publisher: Wiley

ARTICLE INFORMATION
==============================
Article ID: 18440-128
Subjects: C59 - HIV counselling, testing and diagnostic strategies

THAC0405: Peer-delivered HIV testing and counseling among people who inject drugs in Bangkok, Thailand

Ti L. 1 §
Hayashi K. 1 2
Kaplan K. 3
Suwannawong P. 3
Montaner J. 1 4
Wood E. 1 4
Kerr T. 1 4
1 BC Centre for Excellence in HIV/AIDS, Vancouver, Canada
2 University of British Columbia, Interdisciplinary Studies Graduate Program, Vancouver, Canada
3 Thai AIDS Treatment Action Group, Bangkok, Thailand
4 Department of Medicine, University of British Columbia, Vancouver, Canada
§ Presenting author email: lianping.ti@gmail.com
Electronic publication date: 2012 Oct 22
Publication date: 2012
Volume: 15
Issue: Suppl 3
Electronic Location ID: 18440-128
Copyright: © 2012 L. Ti et al.
Copyright year: 2012
License: This is an open-access article distributed under the terms of the Creative Commons Attribution License, which permits unrestricted use, distribution, and reproduction in any medium, provided the original work is properly cited.
License URL: http://creativecommons.org/licenses/by/2.0/

JOURNAL INFORMATION
==============================
NLM Title Abbreviation: J Int AIDS Soc
Journal ID: J Int AIDS Soc
NLM Journal ID: 101478566
Journal ID: JIAS

Journal of the International AIDS Society

EISSN: 1758-2652
Publisher: Wiley

ARTICLE INFORMATION
==============================
Article ID: 18440-129
Subjects: C61 - Prevention for the general population

WEPDC0203: Changes in HIV testing and condom use in Malawi: longitudinal findings at midterm from the Malawi BRIDGE II Project

Rimal R.N. 1
Mkandawire G. 2
Dothi W. 2
Roberts P. 2
Brown J. 3
Limaye R. 3
1 Johns Hopkins Bloomberg School of Public Health, Health, Behavior & Society, Baltimore, United States
2 Johns Hopkins Bloomberg School of Public Health Center for Communication Programs, Lilongwe, Malawi
3 Johns Hopkins Bloomberg School of Public Health Center for Communication Programs, Baltimore, United States
Electronic publication date: 2012 Oct 22
Publication date: 2012
Volume: 15
Issue: Suppl 3
Electronic Location ID: 18440-129
Copyright: © 2012 et al.
Copyright year: 2012
License: This is an open-access article distributed under the terms of the Creative Commons Attribution License, which permits unrestricted use, distribution, and reproduction in any medium, provided the original work is properly cited.
License URL: http://creativecommons.org/licenses/by/2.0/

JOURNAL INFORMATION
==============================
NLM Title Abbreviation: J Int AIDS Soc
Journal ID: J Int AIDS Soc
NLM Journal ID: 101478566
Journal ID: JIAS

Journal of the International AIDS Society

EISSN: 1758-2652
Publisher: Wiley

ARTICLE INFORMATION
==============================
Article ID: 18440-130
Subjects: C62 - Prevention for youth and adolescents

WEAC0102: Transactional sex in South African youth predicted by primary caregiver HIV/AIDS and extreme socio-economic vulnerability: multisite studies

Cluver L. 1 2 3 §
Orkin M. 4
Boyes M. 5
Kuo C. 6
Gardner F. 5
Kganakga M. 7
Limba J. 8
1 Oxford University and University of Cape Town, Social Policy and Intervention, Cape Town, South Africa
2 University of KwaZulu-Natal, HEARD, Durban, South Africa
3 Department of Psychiatry, University of Cape Town, Cape Town, South Africa
4 University of WitwatersrandSchool of Public and Development Management, Johannesburg, South Africa
5 Oxford University, Social Policy and Intervention, Oxford, United Kingdom
6 Department of Psychiatry and Human Behavior, Brown University, Providence, United States
7 Department of Social Development, Government of South Africa, Pretoria, South Africa
8 Cape Town Child Welfare, Cape Town, South Africa
§ Presenting author email: lucie.cluver@spi.ox.ac.uk
Electronic publication date: 2012 Oct 22
Publication date: 2012
Volume: 15
Issue: Suppl 3
Electronic Location ID: 18440-130
Copyright: © 2012 L. Cluver et al.
Copyright year: 2012
License: This is an open-access article distributed under the terms of the Creative Commons Attribution License, which permits unrestricted use, distribution, and reproduction in any medium, provided the original work is properly cited.
License URL: http://creativecommons.org/licenses/by/2.0/

JOURNAL INFORMATION
==============================
NLM Title Abbreviation: J Int AIDS Soc
Journal ID: J Int AIDS Soc
NLM Journal ID: 101478566
Journal ID: JIAS

Journal of the International AIDS Society

EISSN: 1758-2652
Publisher: Wiley

ARTICLE INFORMATION
==============================
Article ID: 18440-131
Subjects: C62 - Prevention for youth and adolescents

WEAC0105: Education and HIV/AIDS in western Kenya: results from a randomized trial assessing the long-term biological and behavioural impact of two school-based interventions

Dupas P. 1
Sharma V. 2 §
Makana G. 3
Nekesa C. 3
Duflo E. 2
1 Stanford University, Palo Alto, United States
2 Massachusetts Institute of Technology, Cambridge, United States
3 Innovations for Poverty Action, Busia, Kenya
§ Presenting author email: vsharma@povertyactionlab.org
Electronic publication date: 2012 Oct 22
Publication date: 2012
Volume: 15
Issue: Suppl 3
Electronic Location ID: 18440-131
Copyright: © 2012 V. Sharma et al.
Copyright year: 2012
License: This is an open-access article distributed under the terms of the Creative Commons Attribution License, which permits unrestricted use, distribution, and reproduction in any medium, provided the original work is properly cited.
License URL: http://creativecommons.org/licenses/by/2.0/

JOURNAL INFORMATION
==============================
NLM Title Abbreviation: J Int AIDS Soc
Journal ID: J Int AIDS Soc
NLM Journal ID: 101478566
Journal ID: JIAS

Journal of the International AIDS Society

EISSN: 1758-2652
Publisher: Wiley

ARTICLE INFORMATION
==============================
Article ID: 18440-132
Subjects: C62 - Prevention for youth and adolescents

WEAE0404: Active program participation and HIV risk reduction among urban youth: findings from the Complementary Strengths Research Partnership

Tiffany J. 1 §
Eckenrode J. 1 2
Exner-Cortens D. 2
Birnel-Henderson S. 1
1 Cornell University, College of Human Ecology, Bronfenbrenner Center for Translational Research, Ithaca, United States
2 Department of Human Development, Cornell University, College of Human Ecology, Ithaca, United States
§ Presenting author email: jst5@cornell.edu
Electronic publication date: 2012 Oct 22
Publication date: 2012
Volume: 15
Issue: Suppl 3
Electronic Location ID: 18440-132
Copyright: © 2012 J. Tiffany et al.
Copyright year: 2012
License: This is an open-access article distributed under the terms of the Creative Commons Attribution License, which permits unrestricted use, distribution, and reproduction in any medium, provided the original work is properly cited.
License URL: http://creativecommons.org/licenses/by/2.0/

JOURNAL INFORMATION
==============================
NLM Title Abbreviation: J Int AIDS Soc
Journal ID: J Int AIDS Soc
NLM Journal ID: 101478566
Journal ID: JIAS

Journal of the International AIDS Society

EISSN: 1758-2652
Publisher: Wiley

ARTICLE INFORMATION
==============================
Article ID: 18440-133
Subjects: C63 - Prevention addressing gender inequalities

WEAD0105: Response to conditional cash transfers: prevention of HIV infection in wives in Pakistan

Khan A. 1 §
Qazi R. 2
Nazim N. 3
Khan A. 1
1 Research and Development Solutions, Islamabad, Pakistan
2 Pakistan Institute of Medical Sciences Hospital, Islamabad, Pakistan
3 Health Services Academy, Islamabad, Pakistan
§ Presenting author email: ayakhan@gmail.com
Electronic publication date: 2012 Oct 22
Publication date: 2012
Volume: 15
Issue: Suppl 3
Electronic Location ID: 18440-133
Copyright: © 2012 A. Khan et al.
Copyright year: 2012
License: This is an open-access article distributed under the terms of the Creative Commons Attribution License, which permits unrestricted use, distribution, and reproduction in any medium, provided the original work is properly cited.
License URL: http://creativecommons.org/licenses/by/2.0/

JOURNAL INFORMATION
==============================
NLM Title Abbreviation: J Int AIDS Soc
Journal ID: J Int AIDS Soc
NLM Journal ID: 101478566
Journal ID: JIAS

Journal of the International AIDS Society

EISSN: 1758-2652
Publisher: Wiley

ARTICLE INFORMATION
==============================
Article ID: 18440-134
Subjects: C64 - Prevention for people who use drugs

THAC0401: The cost-effectiveness of needle-syringe exchange programs in Eastern Europe and Central Asia: costing, data synthesis, modeling and economics for eight case study countries

Wilson D. 1 §
Zhang L. 1
Kerr C. 1
Kwon A. 1
Hoare A. 1
Williams-Sherlock M. 2
Avila C. 3
EECA NSEP evaluation working group
1 University of New South Wales, Sydney, Australia
2 UNAIDS, Moscow, Russian Federation
3 UNAIDS Office, Geneva, Switzerland
§ Presenting author email: dwilson@kirby.unsw.edu.au
Electronic publication date: 2012 Oct 22
Publication date: 2012
Volume: 15
Issue: Suppl 3
Electronic Location ID: 18440-134
Copyright: © 2012 D. Wilson et al.
Copyright year: 2012
License: This is an open-access article distributed under the terms of the Creative Commons Attribution License, which permits unrestricted use, distribution, and reproduction in any medium, provided the original work is properly cited.
License URL: http://creativecommons.org/licenses/by/2.0/

JOURNAL INFORMATION
==============================
NLM Title Abbreviation: J Int AIDS Soc
Journal ID: J Int AIDS Soc
NLM Journal ID: 101478566
Journal ID: JIAS

Journal of the International AIDS Society

EISSN: 1758-2652
Publisher: Wiley

ARTICLE INFORMATION
==============================
Article ID: 18440-135
Subjects: C64 - Prevention for people who use drugs

THAC0402: Switching people who inject drugs from high dead space to low dead space syringes as a structural intervention to prevent injection-related HIV epidemics

Zule W. 1 §
Cross H. 2
1 RTI International, Research Triangle Park, United States
2 RTI International, Global Health Group, Research Triangle Park, United States
§ Presenting author email: zule@rti.org
Electronic publication date: 2012 Oct 22
Publication date: 2012
Volume: 15
Issue: Suppl 3
Electronic Location ID: 18440-135
Copyright: © 2012 W. Zule et al.
Copyright year: 2012
License: This is an open-access article distributed under the terms of the Creative Commons Attribution License, which permits unrestricted use, distribution, and reproduction in any medium, provided the original work is properly cited.
License URL: http://creativecommons.org/licenses/by/2.0/

JOURNAL INFORMATION
==============================
NLM Title Abbreviation: J Int AIDS Soc
Journal ID: J Int AIDS Soc
NLM Journal ID: 101478566
Journal ID: JIAS

Journal of the International AIDS Society

EISSN: 1758-2652
Publisher: Wiley

ARTICLE INFORMATION
==============================
Article ID: 18440-136
Subjects: C64 - Prevention for people who use drugs

THAC0404: Effects of an HIV/AIDS peer prevention intervention on sexual and injecting risk behaviours among injecting drug users (IDU) and their risk partners in Thai Nguyen, Vietnam: a randomized controlled trial

Go V. 1 §
Frangakis C. 2
Nguyen Le M. 3
Tran Viet H. 4
Latkin C. 5
Tran Thi M. 4
Sripaipan T. 1
Davis W. 1
Pham The V. 3
Vu Minh Q. 1
1 Johns Hopkins University, Bloomberg School of Public Health, Epidemiology, Baltimore, United States
2 Johns Hopkins University, Bloomberg School of Public Health, Biostatistics, Baltimore, United States
3 Center for Preventive Medicine, Thai Nguyen, Viet Nam
4 Johns Hopkins University, Bloomberg School of Public Health, Epidemiology, Hanoi, Viet Nam
5 Johns Hopkins University, Bloomberg School of Public Health, Health Behavior and Society, Baltimore, United States
§ Presenting author email: vgo@jhsph.edu
Electronic publication date: 2012 Oct 22
Publication date: 2012
Volume: 15
Issue: Suppl 3
Electronic Location ID: 18440-136
Copyright: © 2012 V. Go et al.
Copyright year: 2012
License: This is an open-access article distributed under the terms of the Creative Commons Attribution License, which permits unrestricted use, distribution, and reproduction in any medium, provided the original work is properly cited.
License URL: http://creativecommons.org/licenses/by/2.0/

JOURNAL INFORMATION
==============================
NLM Title Abbreviation: J Int AIDS Soc
Journal ID: J Int AIDS Soc
NLM Journal ID: 101478566
Journal ID: JIAS

Journal of the International AIDS Society

EISSN: 1758-2652
Publisher: Wiley

ARTICLE INFORMATION
==============================
Article ID: 18440-137
Subjects: C64 - Prevention for people who use drugs

FRLBC06: Heroin scarcity in coastal Kenya: consequences for persons who inject drugs (PWID) and national and provincial response to the crisis

Njenga F. 1
Kiima D. 2
Siminyu M. 3
Munyi E. 4
Abdool R. 5
Muthui M. 6
Lambdin B. 7
Mbwambo J. 8
Mwamburi E. 9
McCurdy S. 10
Pick B. 11
Anderson G. 12
Mital S. 13
Needle R. 14 §
1 National Campaign Against Drug Abuse Authority, Nairobi, Kenya
2 Ministry of Medical Services, Division of Mental Health Services, Nairobi, Kenya
3 Office of the Provincial Director of Medical Services, Coastal Province, Coastal Province, Kenya
4 Office of the Provincial Commissioner, Coastal Province, Coastal Province, Kenya
5 UN Office on Drugs and Crime, Nairobi, Kenya
6 US Centers for Disease Control and Prevention-Kenya, Nairobi, Kenya
7 Pangaea Global AIDS Foundation, Oakland, United States
8 Muhimbili University of Health and Allied Sciences (MUHAS), Dar es Salaam, United Republic of Tanzania
9 United States Agencey for International Development-Kenya, Nairobi, Kenya
10 University of Texas at Austin, School of Public Health, Austin, United States
11 USAID, Washington, United States
12 Centers for Disease Control & Prevention (CDC), Division of Global HIV/AIDS, Atlanta, United States
13 Centers for Disease Control and Prevention (CDC), Atlanta, United States
14 US Office of Global AIDS Coordinator, Washington, United States
§ Presenting author email: fnjenga@africaonline.co.ke
Electronic publication date: 2012 Oct 22
Publication date: 2012
Volume: 15
Issue: Suppl 3
Electronic Location ID: 18440-137
Copyright: © 2012 R. Needle et al.
Copyright year: 2012
License: This is an open-access article distributed under the terms of the Creative Commons Attribution License, which permits unrestricted use, distribution, and reproduction in any medium, provided the original work is properly cited.
License URL: http://creativecommons.org/licenses/by/2.0/

JOURNAL INFORMATION
==============================
NLM Title Abbreviation: J Int AIDS Soc
Journal ID: J Int AIDS Soc
NLM Journal ID: 101478566
Journal ID: JIAS

Journal of the International AIDS Society

EISSN: 1758-2652
Publisher: Wiley

ARTICLE INFORMATION
==============================
Article ID: 18440-138
Subjects: C66 - Prevention for men who have sex with men (MSM)

TUPDC0304: Use of a rapid HIV home test to screen potential sexual partners prevents HIV exposure in a high-risk sample of MSM

Carballo-Diéguez A. 1
Balan I. 1
Frasca T. 1
Dolezal C. 1
Valladares J. 1 §
1 HIV Center for Clinical and Behavioral Studies, NYS Psychiatric Institute and Columbia University, New York, United States
§ Presenting author email: ac72@columbia.edu
Electronic publication date: 2012 Oct 22
Publication date: 2012
Volume: 15
Issue: Suppl 3
Electronic Location ID: 18440-138
Copyright: © 2012 J. Valladares et al.
Copyright year: 2012
License: This is an open-access article distributed under the terms of the Creative Commons Attribution License, which permits unrestricted use, distribution, and reproduction in any medium, provided the original work is properly cited.
License URL: http://creativecommons.org/licenses/by/2.0/

JOURNAL INFORMATION
==============================
NLM Title Abbreviation: J Int AIDS Soc
Journal ID: J Int AIDS Soc
NLM Journal ID: 101478566
Journal ID: JIAS

Journal of the International AIDS Society

EISSN: 1758-2652
Publisher: Wiley

ARTICLE INFORMATION
==============================
Article ID: 18440-139
Subjects: C66 - Prevention for men who have sex with men (MSM)

THPDC0105: Switzerland has developed a new strategy to break the chains of new HIV infections among gay men and other MSM

Derendinger S. 1
Staub R. 1 §
1 Swiss Federal Office of Public Health, Communicable Diseases, Bern, Switzerland
§ Presenting author email: steven.derendinger@bag.admin.ch
Electronic publication date: 2012 Oct 22
Publication date: 2012
Volume: 15
Issue: Suppl 3
Electronic Location ID: 18440-139
Copyright: © 2012 R. Staub et al.
Copyright year: 2012
License: This is an open-access article distributed under the terms of the Creative Commons Attribution License, which permits unrestricted use, distribution, and reproduction in any medium, provided the original work is properly cited.
License URL: http://creativecommons.org/licenses/by/2.0/

JOURNAL INFORMATION
==============================
NLM Title Abbreviation: J Int AIDS Soc
Journal ID: J Int AIDS Soc
NLM Journal ID: 101478566
Journal ID: JIAS

Journal of the International AIDS Society

EISSN: 1758-2652
Publisher: Wiley

ARTICLE INFORMATION
==============================
Article ID: 18440-140
Subjects: C67 - Prevention for transgenders

THPDC0203: HIV testing among transgender persons funded by the Centers for Disease Control and Prevention in the United States, Puerto Rico and U.S. Virgin Islands, 2008–2009

Habarta N. 1
Wang G. 1
Mulatu M. 1 §
1 Centers for Disease Control and Prevention, Division of HIV/AIDS Prevention, Atlanta, United States
§ Presenting author email: nhabarta@cdc.gov
Electronic publication date: 2012 Oct 22
Publication date: 2012
Volume: 15
Issue: Suppl 3
Electronic Location ID: 18440-140
Copyright: © 2012 M. Mulatu et al.
Copyright year: 2012
License: This is an open-access article distributed under the terms of the Creative Commons Attribution License, which permits unrestricted use, distribution, and reproduction in any medium, provided the original work is properly cited.
License URL: http://creativecommons.org/licenses/by/2.0/

JOURNAL INFORMATION
==============================
NLM Title Abbreviation: J Int AIDS Soc
Journal ID: J Int AIDS Soc
NLM Journal ID: 101478566
Journal ID: JIAS

Journal of the International AIDS Society

EISSN: 1758-2652
Publisher: Wiley

ARTICLE INFORMATION
==============================
Article ID: 18440-141
Subjects: C67 - Prevention for transgenders

THPDC0204: Transgender in Tamil Nadu are still highly vulnerable to HIV and STIs: findings from bio-behavioral surveys

Ramakrishnan L. 1
Goswami P. 1
Subramaniam T. 2
Mathew S. 1
Ramanathan S. 1
George B. 1
Adhikary R. 3
Mainkar M.K. 4
Paranjape R.S. 4 §
1 FHI 360, India, New Delhi, India
2 National Institute of Epidemiology (NIE), ICMR, Chennai, India
3 FHI 360, Washington, Washington, United States
4 National AIDS Research Institute-ICMR, Pune, India
§ Presenting author email: lramakrishnan@fhiindia.org
Electronic publication date: 2012 Oct 22
Publication date: 2012
Volume: 15
Issue: Suppl 3
Electronic Location ID: 18440-141
Copyright: © 2012 R.S. Paranjape et al.
Copyright year: 2012
License: This is an open-access article distributed under the terms of the Creative Commons Attribution License, which permits unrestricted use, distribution, and reproduction in any medium, provided the original work is properly cited.
License URL: http://creativecommons.org/licenses/by/2.0/

JOURNAL INFORMATION
==============================
NLM Title Abbreviation: J Int AIDS Soc
Journal ID: J Int AIDS Soc
NLM Journal ID: 101478566
Journal ID: JIAS

Journal of the International AIDS Society

EISSN: 1758-2652
Publisher: Wiley

ARTICLE INFORMATION
==============================
Article ID: 18440-142
Subjects: C70 - Preventing transmission from people living with HIV

MOAC0201: Predictors for high vireamia among a treatment-naïve national HIV cohort in the United Kingdom

Brown A. 1
Aghaizu A. 1
Murphy G. 1
Delpech V. 1 §
1 Heatlh Protection Agency, London, United Kingdom
§ Presenting author email: alison.brown@hpa.org.uk
Electronic publication date: 2012 Oct 22
Publication date: 2012
Volume: 15
Issue: Suppl 3
Electronic Location ID: 18440-142
Copyright: © 2012 V. Delpech et al.
Copyright year: 2012
License: This is an open-access article distributed under the terms of the Creative Commons Attribution License, which permits unrestricted use, distribution, and reproduction in any medium, provided the original work is properly cited.
License URL: http://creativecommons.org/licenses/by/2.0/

JOURNAL INFORMATION
==============================
NLM Title Abbreviation: J Int AIDS Soc
Journal ID: J Int AIDS Soc
NLM Journal ID: 101478566
Journal ID: JIAS

Journal of the International AIDS Society

EISSN: 1758-2652
Publisher: Wiley

ARTICLE INFORMATION
==============================
Article ID: 18440-143
Subjects: C70 - Preventing transmission from people living with HIV

THAC0201: The threshold for an ART secondary prevention effect on HIV transmission among men who have sex with men (MSM) has not been reached in the UK despite high treatment uptake

Brown A. 1
Gill O.N. 1
Presanis A. 2
Delpech V. 1
Murphy G. 1 §
1 Heatlh Protection Agency, London, United Kingdom
2 Institute of Public Health, MRC Biostatistics Unit, Cambridge, United Kingdom
§ Presenting author email: alison.brown@hpa.org.uk
Electronic publication date: 2012 Oct 22
Publication date: 2012
Volume: 15
Issue: Suppl 3
Electronic Location ID: 18440-143
Copyright: © 2012 G. Murphy et al.
Copyright year: 2012
License: This is an open-access article distributed under the terms of the Creative Commons Attribution License, which permits unrestricted use, distribution, and reproduction in any medium, provided the original work is properly cited.
License URL: http://creativecommons.org/licenses/by/2.0/

JOURNAL INFORMATION
==============================
NLM Title Abbreviation: J Int AIDS Soc
Journal ID: J Int AIDS Soc
NLM Journal ID: 101478566
Journal ID: JIAS

Journal of the International AIDS Society

EISSN: 1758-2652
Publisher: Wiley

ARTICLE INFORMATION
==============================
Article ID: 18440-144
Subjects: C70 - Preventing transmission from people living with HIV

TUPDC0203: Four years after the Swiss Statement: transmission risk decision making in sero-different stable couples

Nicca D. 1 §
Rasi M. 1
Stoelzl S. 1
Schmid P. 1
Kahlert C. 1
Vernazza P. 1
Swiss HIV Cohort Study 1
1 Cantonal Hospital, St. Gallen, Infectious Diseases and Hospital Hygiene, St. Gallen, Switzerland
§ Presenting author email: dunja.nicca@kssg.ch
Electronic publication date: 2012 Oct 22
Publication date: 2012
Volume: 15
Issue: Suppl 3
Electronic Location ID: 18440-144
Copyright: © 2012 D. Nicca et al.
Copyright year: 2012
License: This is an open-access article distributed under the terms of the Creative Commons Attribution License, which permits unrestricted use, distribution, and reproduction in any medium, provided the original work is properly cited.
License URL: http://creativecommons.org/licenses/by/2.0/

JOURNAL INFORMATION
==============================
NLM Title Abbreviation: J Int AIDS Soc
Journal ID: J Int AIDS Soc
NLM Journal ID: 101478566
Journal ID: JIAS

Journal of the International AIDS Society

EISSN: 1758-2652
Publisher: Wiley

ARTICLE INFORMATION
==============================
Article ID: 18440-145
Subjects: C70 - Preventing transmission from people living with HIV

WEPDC0205: Effectiveness of behavioral interventions on unprotected sex among people living with HIV: a system review and meta-analysis

Yin L. 1
Wang N. 2
Ruan Y. 2
Shao Y. 2
Vermund S. 1
Qian H.-Z. 1 §
1 Vanderbilt Institute for Global Health, Nashville, United States
2 National Center for AIDS/STD Control and Prevention, China CDC, Division of Virology and Immunology, Beijing, China
§ Presenting author email: lu.yin@vanderbilt.edu
Electronic publication date: 2012 Oct 22
Publication date: 2012
Volume: 15
Issue: Suppl 3
Electronic Location ID: 18440-145
Copyright: © 2012 H.-Z. Qian et al.
Copyright year: 2012
License: This is an open-access article distributed under the terms of the Creative Commons Attribution License, which permits unrestricted use, distribution, and reproduction in any medium, provided the original work is properly cited.
License URL: http://creativecommons.org/licenses/by/2.0/

JOURNAL INFORMATION
==============================
NLM Title Abbreviation: J Int AIDS Soc
Journal ID: J Int AIDS Soc
NLM Journal ID: 101478566
Journal ID: JIAS

Journal of the International AIDS Society

EISSN: 1758-2652
Publisher: Wiley

ARTICLE INFORMATION
==============================
Article ID: 18440-146
Subjects: C72 - Use of the internet and mobile phones for prevention

WEAC0104: Soap opera video episodes streamed to smartphones in a randomized controlled trial to reduce HIV sex risk in young urban African American/black women

Jones R. 1
Lacroix L. 1
Hoover D. 2 §
1 Rutgers University, College of Nursing, Newark, United States
2 Rutgers University, Department of Statistics, Piscataway, United States
§ Presenting author email: racjones@rutgers.edu
Electronic publication date: 2012 Oct 22
Publication date: 2012
Volume: 15
Issue: Suppl 3
Electronic Location ID: 18440-146
Copyright: © 2012 D. Hoover et al.
Copyright year: 2012
License: This is an open-access article distributed under the terms of the Creative Commons Attribution License, which permits unrestricted use, distribution, and reproduction in any medium, provided the original work is properly cited.
License URL: http://creativecommons.org/licenses/by/2.0/

JOURNAL INFORMATION
==============================
NLM Title Abbreviation: J Int AIDS Soc
Journal ID: J Int AIDS Soc
NLM Journal ID: 101478566
Journal ID: JIAS

Journal of the International AIDS Society

EISSN: 1758-2652
Publisher: Wiley

ARTICLE INFORMATION
==============================
Article ID: 18440-147
Subjects: C75 - Integration of HIV prevention and other health programmes

WEPDC0204: Integration of HIV prevention with health and nutrition program involving self help group women in Andhra Pradesh, India

Sivalenka S. 1
Nitrahally Mallachar C. 2
Chava L.D. 3
Ravi S.P. 2 §
1 Centers for Disease Control and Prevention (CDC), Global AIDS Program, Hyderabad, India
2 Lepra Society, Secunderabad, India
3 Society for Elimination of Rural Poverty, Hyderabad, India
§ Presenting author email: sivalenkas@in.cdc.gov
Electronic publication date: 2012 Oct 22
Publication date: 2012
Volume: 15
Issue: Suppl 3
Electronic Location ID: 18440-147
Copyright: © 2012 S.P. Ravi et al.
Copyright year: 2012
License: This is an open-access article distributed under the terms of the Creative Commons Attribution License, which permits unrestricted use, distribution, and reproduction in any medium, provided the original work is properly cited.
License URL: http://creativecommons.org/licenses/by/2.0/

JOURNAL INFORMATION
==============================
NLM Title Abbreviation: J Int AIDS Soc
Journal ID: J Int AIDS Soc
NLM Journal ID: 101478566
Journal ID: JIAS

Journal of the International AIDS Society

EISSN: 1758-2652
Publisher: Wiley

ARTICLE INFORMATION
==============================
Article ID: 18440-148
Subjects: C76 - Structural interventions for HIV prevention

THAC0301: Structural determinants in MSM HIV preventionenvironmental and structural factors predict internalised homonegativity in men who have sex with men (MSM): findings from the European MSM internet survey (EMIS) in 38 countries

Berg R.C. 1 2
Ross M.W. 2 3
Schmidt A.J. 4
Weatherburn P. 4
EMIS Network §
1 Norwegian Knowledge Centre for the Health Services, Dept of Evidence-Based Health Services, Oslo, Norway
2 University of Texas Health Sciences Center, Houston, School of Public Health, Houston, United States
3 Malmö University, Faculty of Health and Society, Malmö, Sweden
4 London School of Hygiene and Tropical Medicine, London, United Kingdom
§
Electronic publication date: 2012 Oct 22
Publication date: 2012
Volume: 15
Issue: Suppl 3
Electronic Location ID: 18440-148
Copyright: © 2012 R.C. Berg et al.
Copyright year: 2012
License: This is an open-access article distributed under the terms of the Creative Commons Attribution License, which permits unrestricted use, distribution, and reproduction in any medium, provided the original work is properly cited.
License URL: http://creativecommons.org/licenses/by/2.0/

JOURNAL INFORMATION
==============================
NLM Title Abbreviation: J Int AIDS Soc
Journal ID: J Int AIDS Soc
NLM Journal ID: 101478566
Journal ID: JIAS

Journal of the International AIDS Society

EISSN: 1758-2652
Publisher: Wiley

ARTICLE INFORMATION
==============================
Article ID: 18440-149
Subjects: C76 - Structural interventions for HIV prevention

TUPDE0102: Conditional cash transfers improve birth registration and school attendance amongst orphans and vulnerable children in Manicaland, Zimbabwe

Robertson L. 1
Mushati P. 2
Eaton J.W. 1
Dumba L. 3
Mavise G. 3
Makoni J.C. 4
Schumacher C. 1
Crea T. 5
Monasch R. 6
Sherr L. 7
Garnett G.P. 1
Nyamukapa C. 1
Gregson S. 1 §
1 Imperial College London, Infectious Disease Epidemiology, London, United Kingdom
2 Biomedical Research & Training Institute, Harare, Zimbabwe
3 Catholic Relief Services, Harare, Zimbabwe
4 DOMCCP, Mutasa, Zimbabwe
5 Boston College, Graduate School of Social Work, Boston, United States
6 UNICEF, Harare, Zimbabwe
7 University College London, Infection and Population Health, London, United Kingdom
§ Presenting author email: l.robertson06@imperial.ac.uk
Electronic publication date: 2012 Oct 22
Publication date: 2012
Volume: 15
Issue: Suppl 3
Electronic Location ID: 18440-149
Copyright: © 2012 S. Gregson et al.
Copyright year: 2012
License: This is an open-access article distributed under the terms of the Creative Commons Attribution License, which permits unrestricted use, distribution, and reproduction in any medium, provided the original work is properly cited.
License URL: http://creativecommons.org/licenses/by/2.0/

JOURNAL INFORMATION
==============================
NLM Title Abbreviation: J Int AIDS Soc
Journal ID: J Int AIDS Soc
NLM Journal ID: 101478566
Journal ID: JIAS

Journal of the International AIDS Society

EISSN: 1758-2652
Publisher: Wiley

ARTICLE INFORMATION
==============================
Article ID: 18440-150
Subjects: C83 - Reproductive choices and maternal health

WEAC0103: The next generation: perinatally infected adolescents and their reproductive health

Tepper V. 1
Kriebs J. 2
Lovelace S. 1
Nair P. 1 §
1 University of Maryland School of Medicine, Pediatrics, Baltimore, United States
2 University of Maryland School of Medicine, Department of Obstetrics, Gynecology and Reproductive Sciences, Baltimore, United States
§ Presenting author email: vtepper@peds.umaryland.edu
Electronic publication date: 2012 Oct 22
Publication date: 2012
Volume: 15
Issue: Suppl 3
Electronic Location ID: 18440-150
Copyright: © 2012 P. Nair et al.
Copyright year: 2012
License: This is an open-access article distributed under the terms of the Creative Commons Attribution License, which permits unrestricted use, distribution, and reproduction in any medium, provided the original work is properly cited.
License URL: http://creativecommons.org/licenses/by/2.0/

JOURNAL INFORMATION
==============================
NLM Title Abbreviation: J Int AIDS Soc
Journal ID: J Int AIDS Soc
NLM Journal ID: 101478566
Journal ID: JIAS

Journal of the International AIDS Society

EISSN: 1758-2652
Publisher: Wiley

ARTICLE INFORMATION
==============================
Article ID: 18440-151
Subjects: C83 - Reproductive choices and maternal health

THAC0101: Unplanned pregnancy in the 2011 Botswana Antenatal Clinic Sentinel Surveillance

Voetsch A.C. 1
Anderson M.G. 2
Machakaire E. 1
Bodika S. 1
Jimbo W. 1
Yadav B.P. 2
Schaan M. 3
Madidimalo T. 2
Lebelonyane R. 2 §
1 U.S. Centers for Disease Control and Prevention, Division of Global HIV/AIDS, Gaborone, Botswana
2 Ministry of Health, Department of HIV/AIDS Prevention and Care, Gaborone, Botswana
3 Botswana-Harvard Partnership for HIV Research and Education, Gaborone, Botswana
§ Presenting author email: voetschd@bw.cdc.gov
Electronic publication date: 2012 Oct 22
Publication date: 2012
Volume: 15
Issue: Suppl 3
Electronic Location ID: 18440-151
Copyright: © 2012 R. Lebelonyane et al.
Copyright year: 2012
License: This is an open-access article distributed under the terms of the Creative Commons Attribution License, which permits unrestricted use, distribution, and reproduction in any medium, provided the original work is properly cited.
License URL: http://creativecommons.org/licenses/by/2.0/

JOURNAL INFORMATION
==============================
NLM Title Abbreviation: J Int AIDS Soc
Journal ID: J Int AIDS Soc
NLM Journal ID: 101478566
Journal ID: JIAS

Journal of the International AIDS Society

EISSN: 1758-2652
Publisher: Wiley

ARTICLE INFORMATION
==============================
Article ID: 18440-152
Subjects: C83 - Reproductive choices and maternal health

THAC0106: Where do women who deliver at home fall through cracks in the PMTCT continuum of care services? A retrospective study of service uptake among mothers who delivered at home in Zimbabwe

Webb K.A. 1
Patel D. 1
Mujaranji G. 1
Engelsmann B. 1 §
1 Organisation for Public Health Interventions and Development (OPHID) Trust, Harare, Zimbabwe
§ Presenting author email: karen.myllynen.webb@gmail.com
Electronic publication date: 2012 Oct 22
Publication date: 2012
Volume: 15
Issue: Suppl 3
Electronic Location ID: 18440-152
Copyright: © 2012 B. Engelsmann et al.
Copyright year: 2012
License: This is an open-access article distributed under the terms of the Creative Commons Attribution License, which permits unrestricted use, distribution, and reproduction in any medium, provided the original work is properly cited.
License URL: http://creativecommons.org/licenses/by/2.0/

JOURNAL INFORMATION
==============================
NLM Title Abbreviation: J Int AIDS Soc
Journal ID: J Int AIDS Soc
NLM Journal ID: 101478566
Journal ID: JIAS

Journal of the International AIDS Society

EISSN: 1758-2652
Publisher: Wiley

ARTICLE INFORMATION
==============================
Article ID: 18440-153
Subjects: C83 - Reproductive choices and maternal health

TUPDC0202: Safer conception options for HIV serodiscordant couples in the United States: experience of the National Perinatal HIV Hotline and Clinicians' Network

Weber S. 1
Waldura J. 1
Cohan D. 2 §
1 UCSF, Family & Community Medicine, San Francisco, United States
2 UCSF, Obstetrics & Gynecology, San Francisco, United States
§ Presenting author email: sweber@nccc.ucsf.edu
Electronic publication date: 2012 Oct 22
Publication date: 2012
Volume: 15
Issue: Suppl 3
Electronic Location ID: 18440-153
Copyright: © 2012 D. Cohan et al.
Copyright year: 2012
License: This is an open-access article distributed under the terms of the Creative Commons Attribution License, which permits unrestricted use, distribution, and reproduction in any medium, provided the original work is properly cited.
License URL: http://creativecommons.org/licenses/by/2.0/

